# Author Correction: Spatial covariance analysis reveals the residue-by-residue thermodynamic contribution of variation to the CFTR fold

**DOI:** 10.1038/s42003-022-03457-y

**Published:** 2022-05-18

**Authors:** Frédéric Anglès, Chao Wang, William E. Balch

**Affiliations:** Scripps Research, Department of Molecular Medicine, 10550 North Torrey Pines Rd, La Jolla, CA 92037 USA

**Keywords:** Mechanisms of disease, Protein folding, Mutation, Endoplasmic reticulum, Protein transport

Correction to: *Communications Biology* 10.1038/s42003-022-03302-2, published online 13 April 2022.

The wrong Supplementary file was originally published with this article; it has now been replaced with the correct. The original article has been corrected.

## Supplementary information

Updated Supplementary Information

