# Supplementary material for: Spatial covariance analysis reveals the residue-by-residue thermodynamic contribution of variation to the CFTR fold
Source: Commun Biol. 2022 Apr 13;5:356. doi: 10.1038/s42003-022-03302-2 (PMC9008016; doi:10.1038/s42003-022-03302-2)
Supplement: Supplementary file 2 — Supplementary Information (new) [file 42003_2022_3302_MOESM2_ESM.pdf]

Supplementary Figure 1

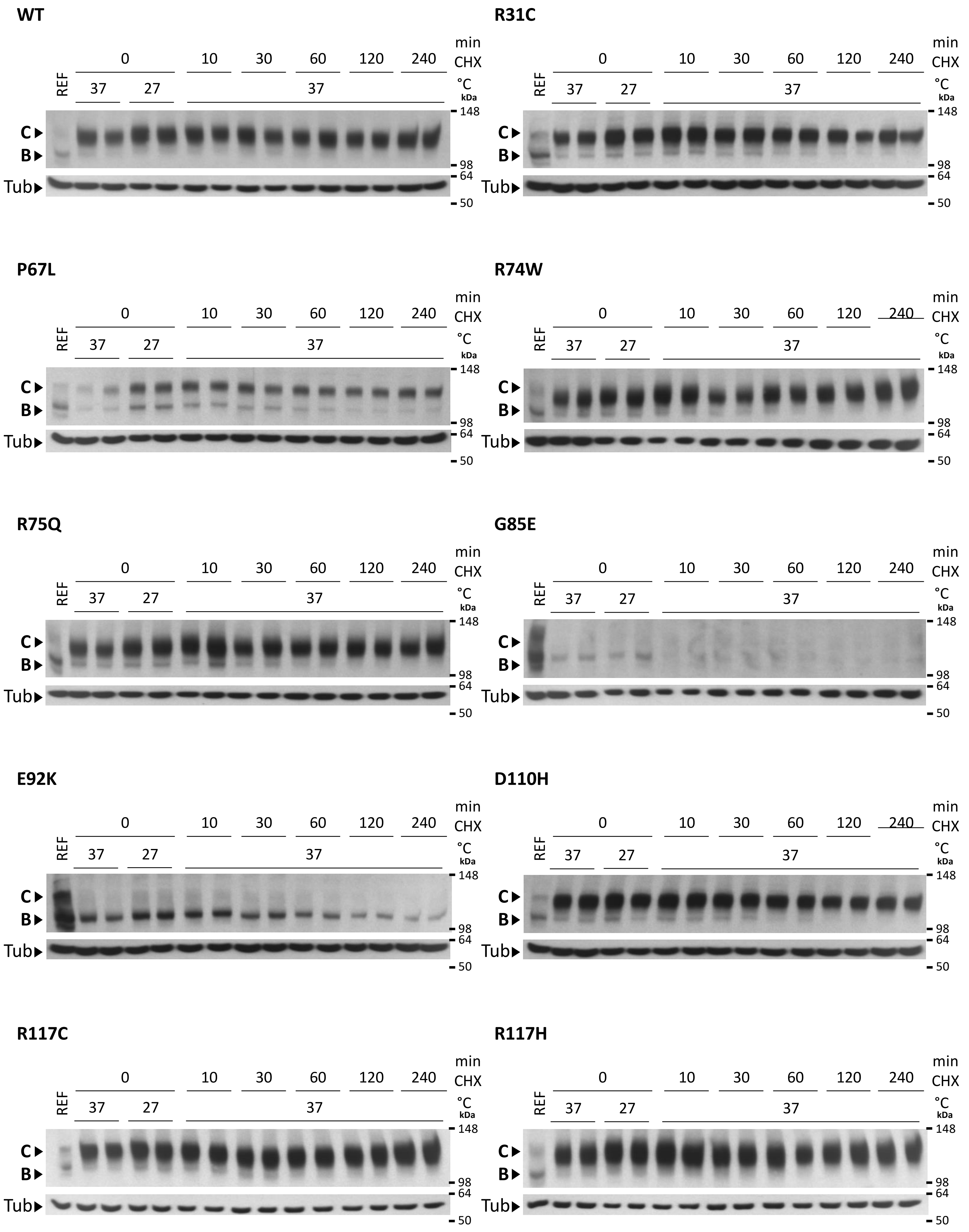

Supplementary Figure 2

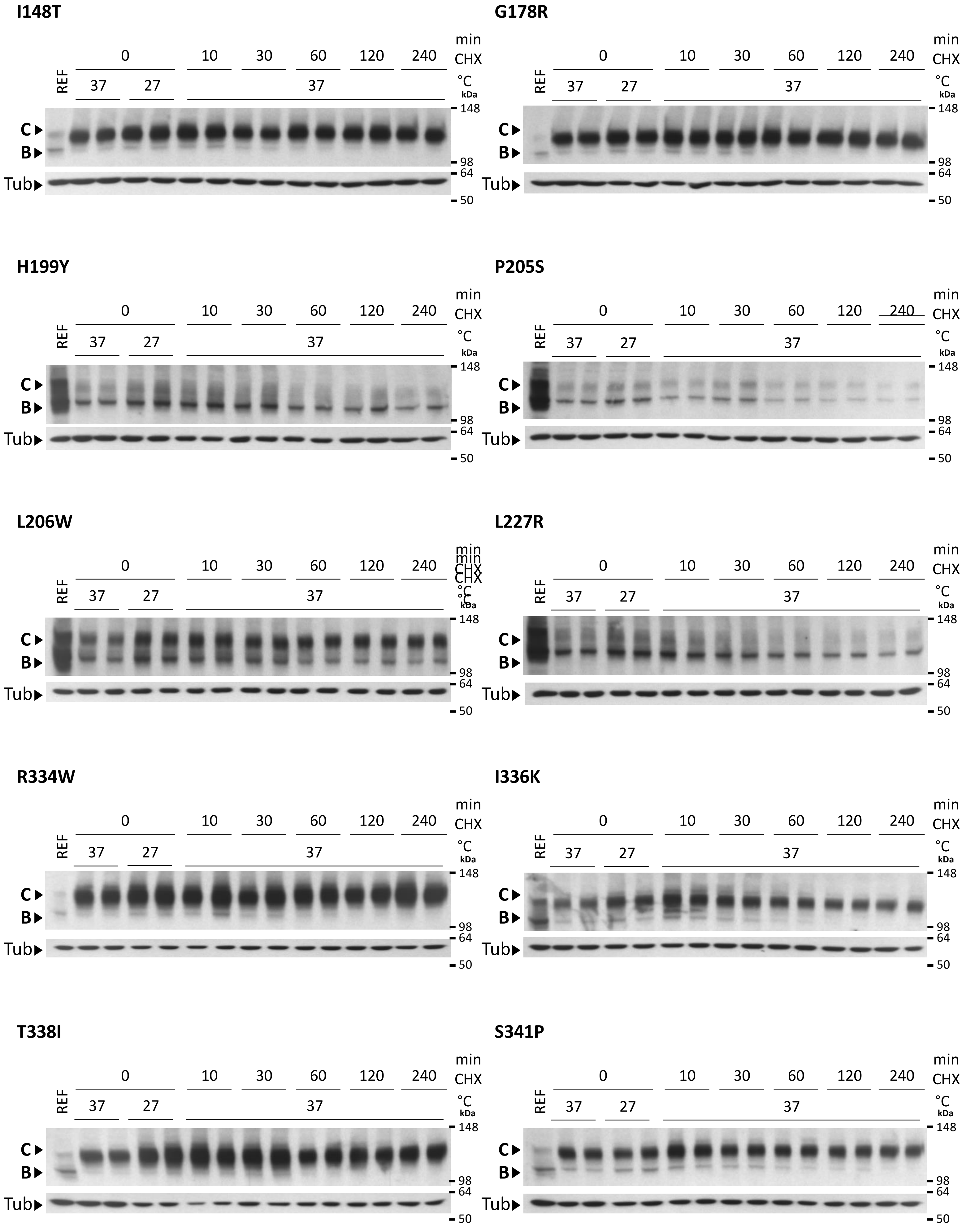

Supplementary Figure 3

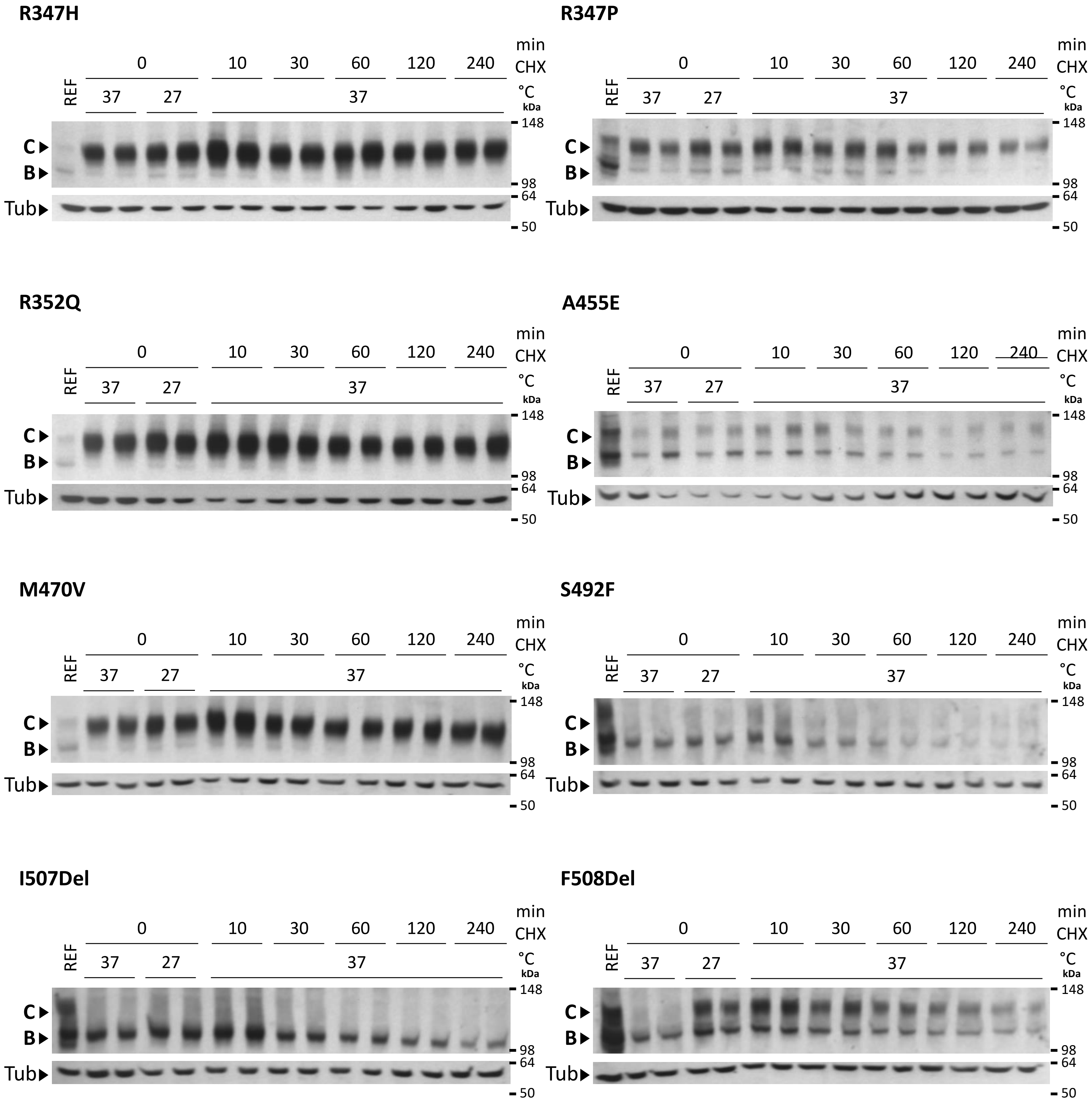

Supplementary Figure 4

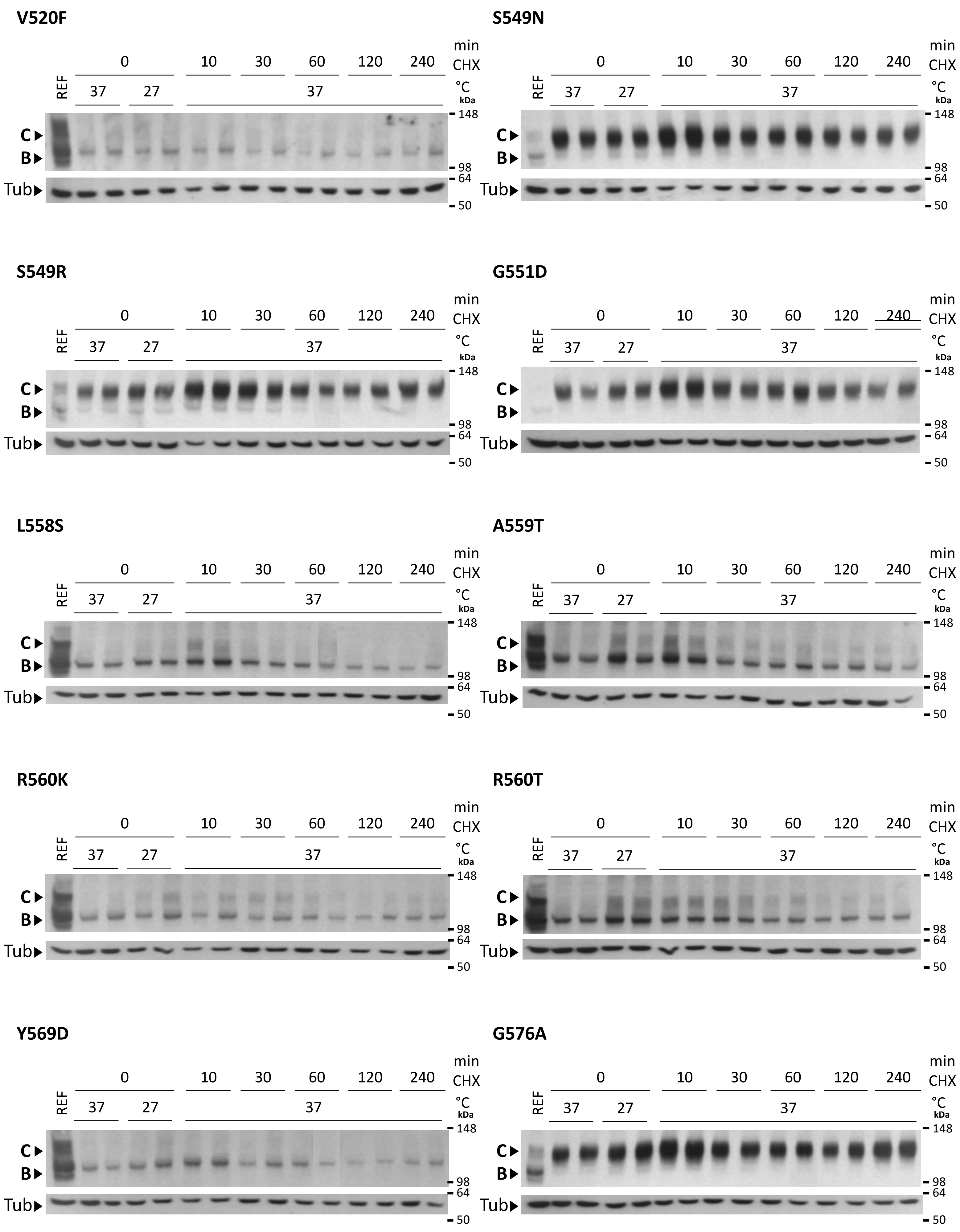

Supplementary Figure 5

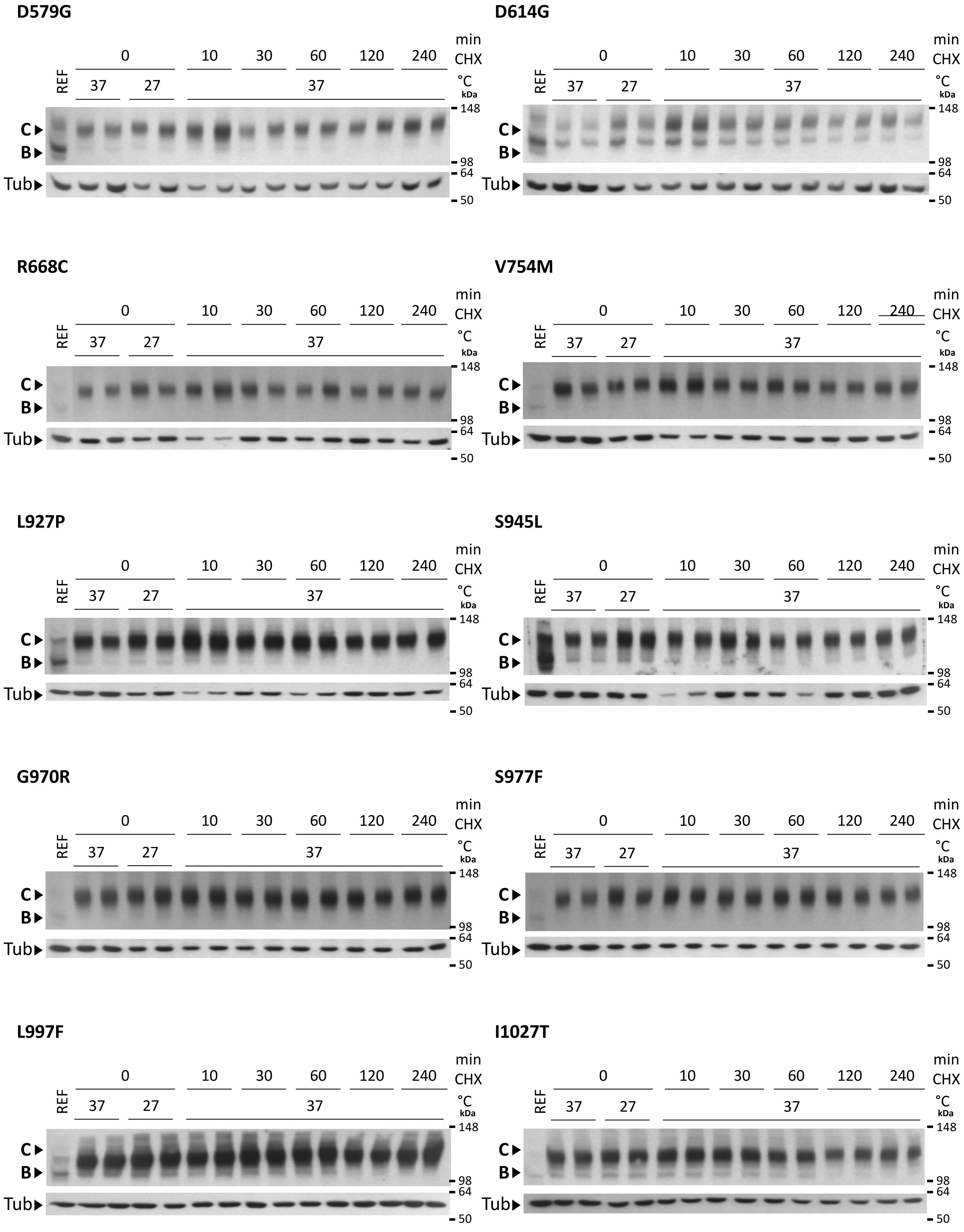

Supplementary Figure 6

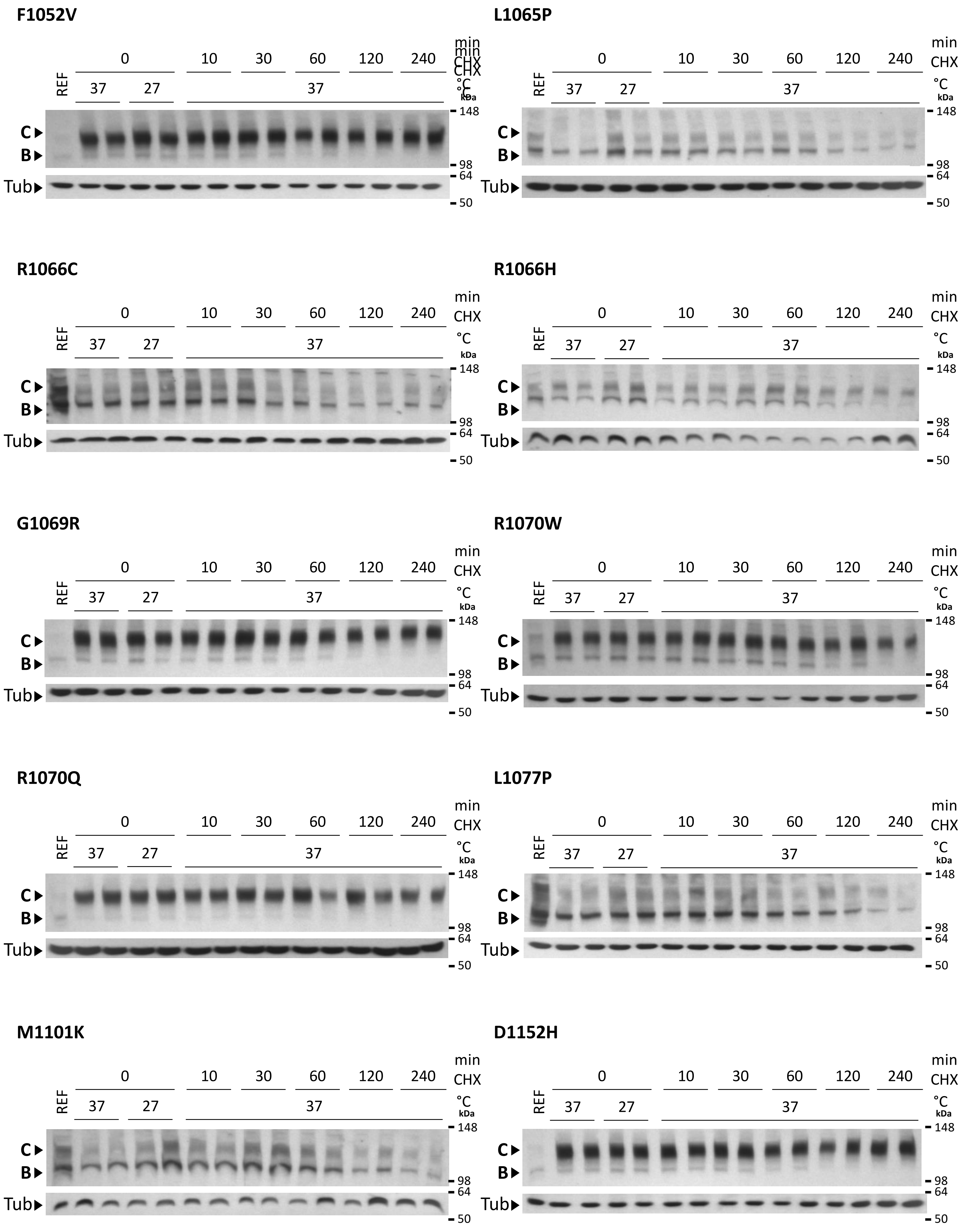

Supplementary Figure 7

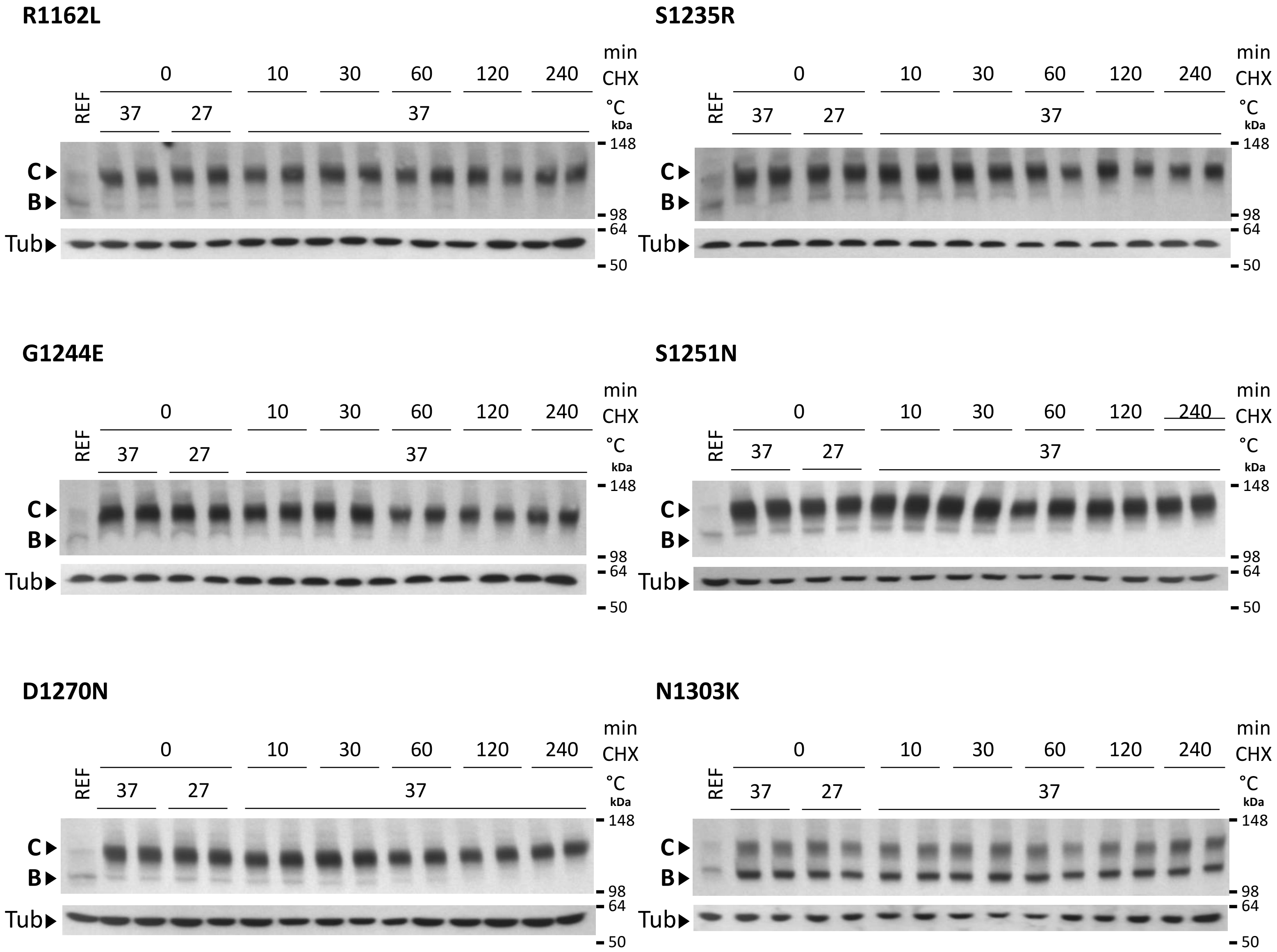

Supplementary Figure 8

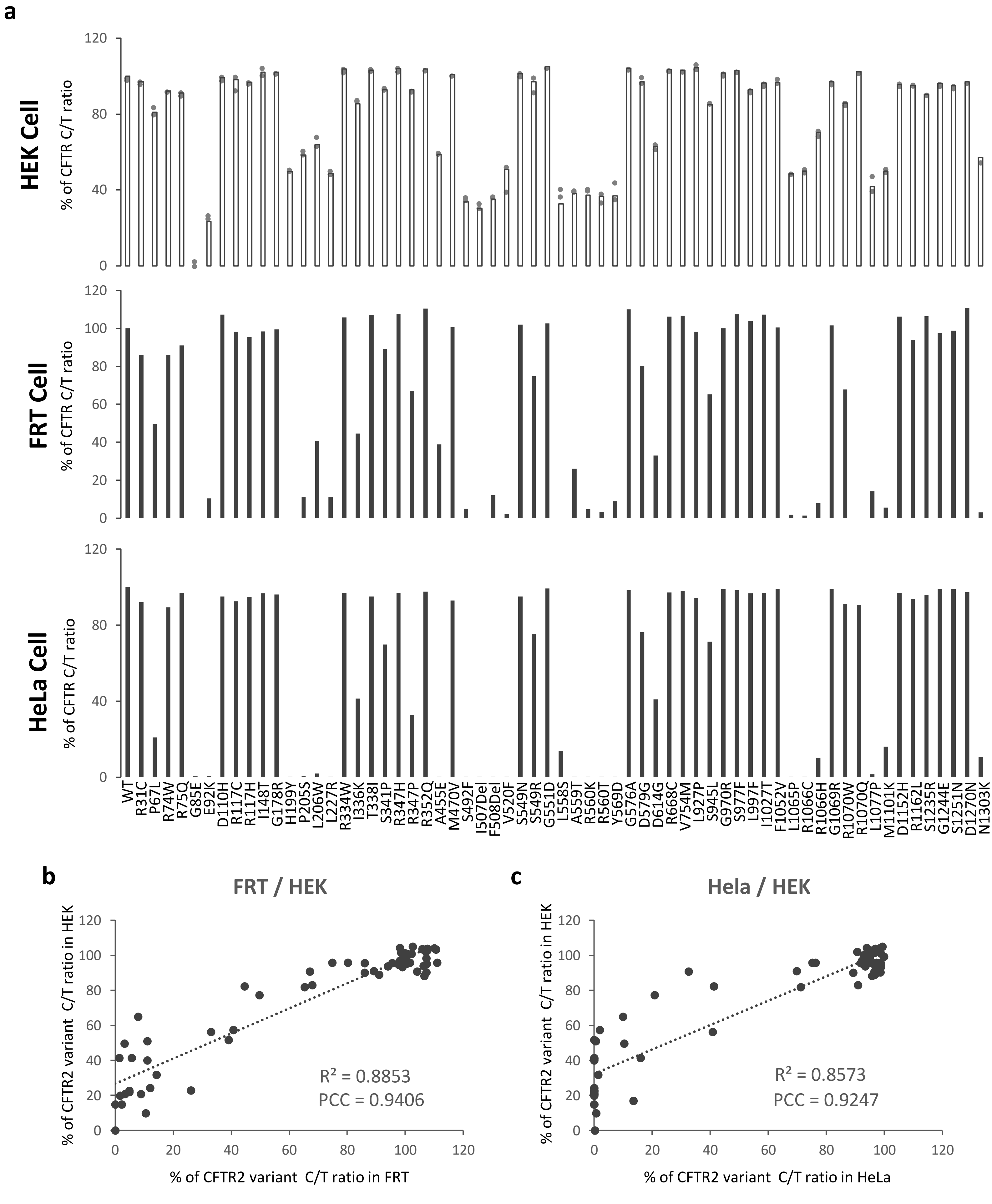

Supplementary Figure 9

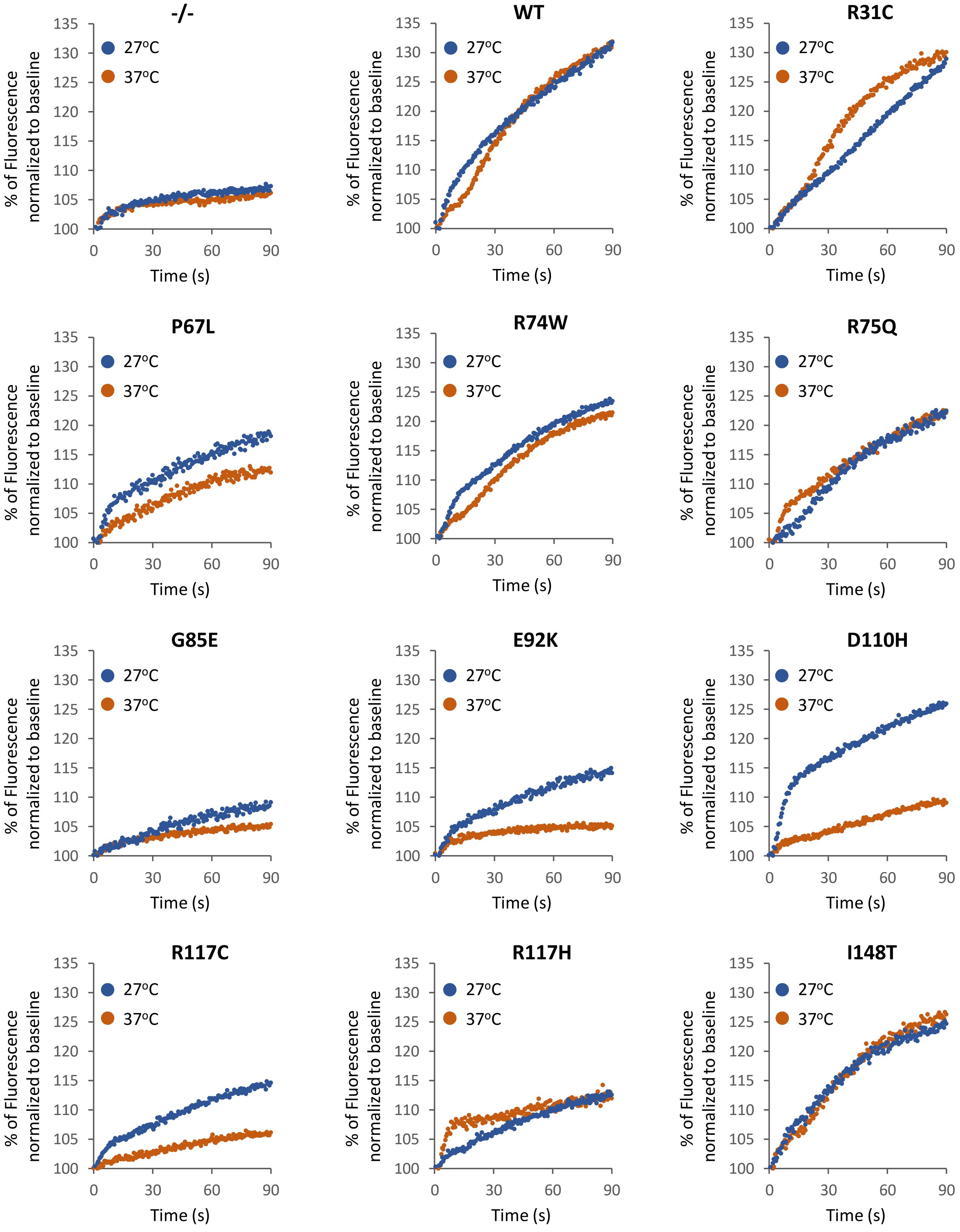

Supplementary Figure 10

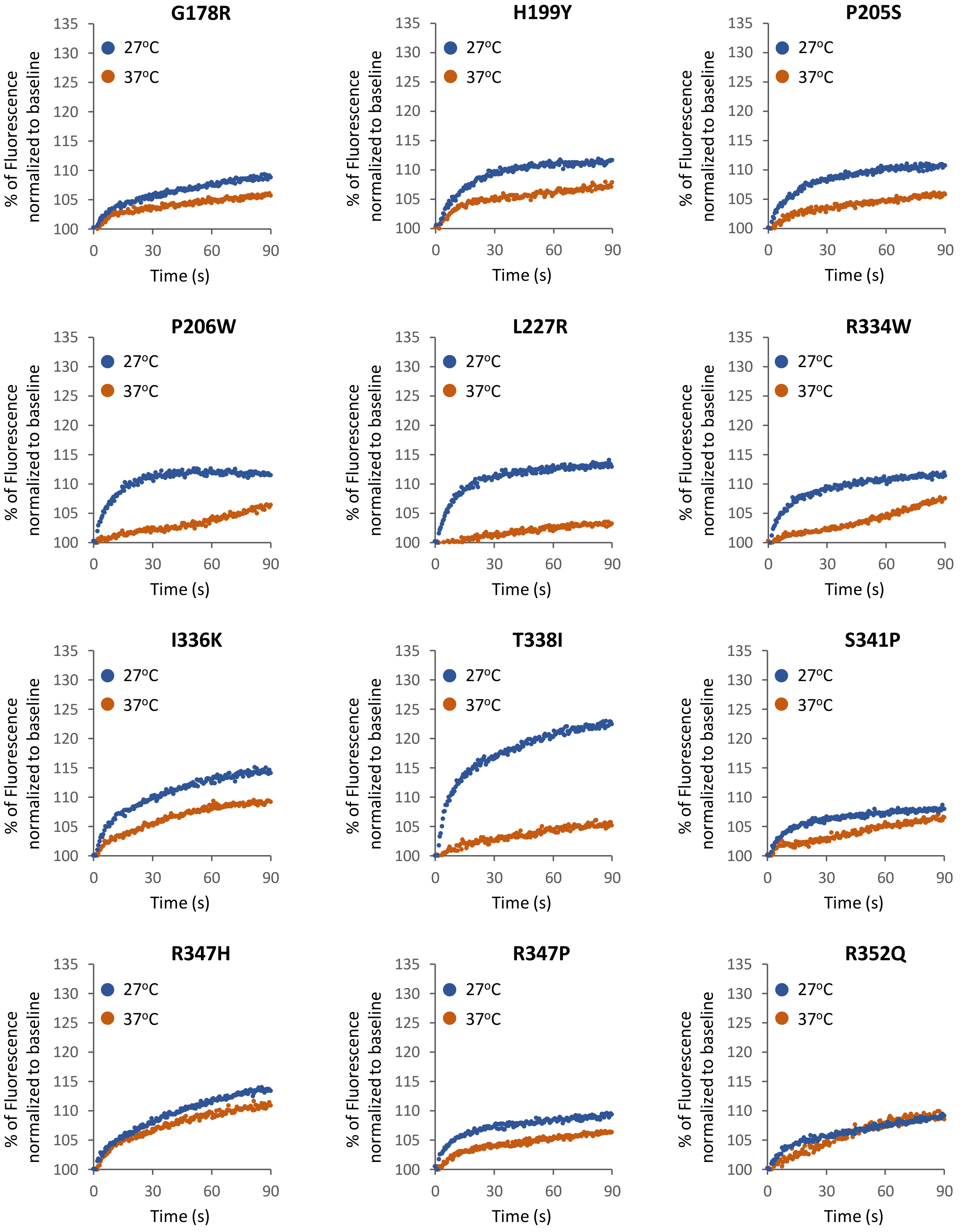

Supplementary Figure 11

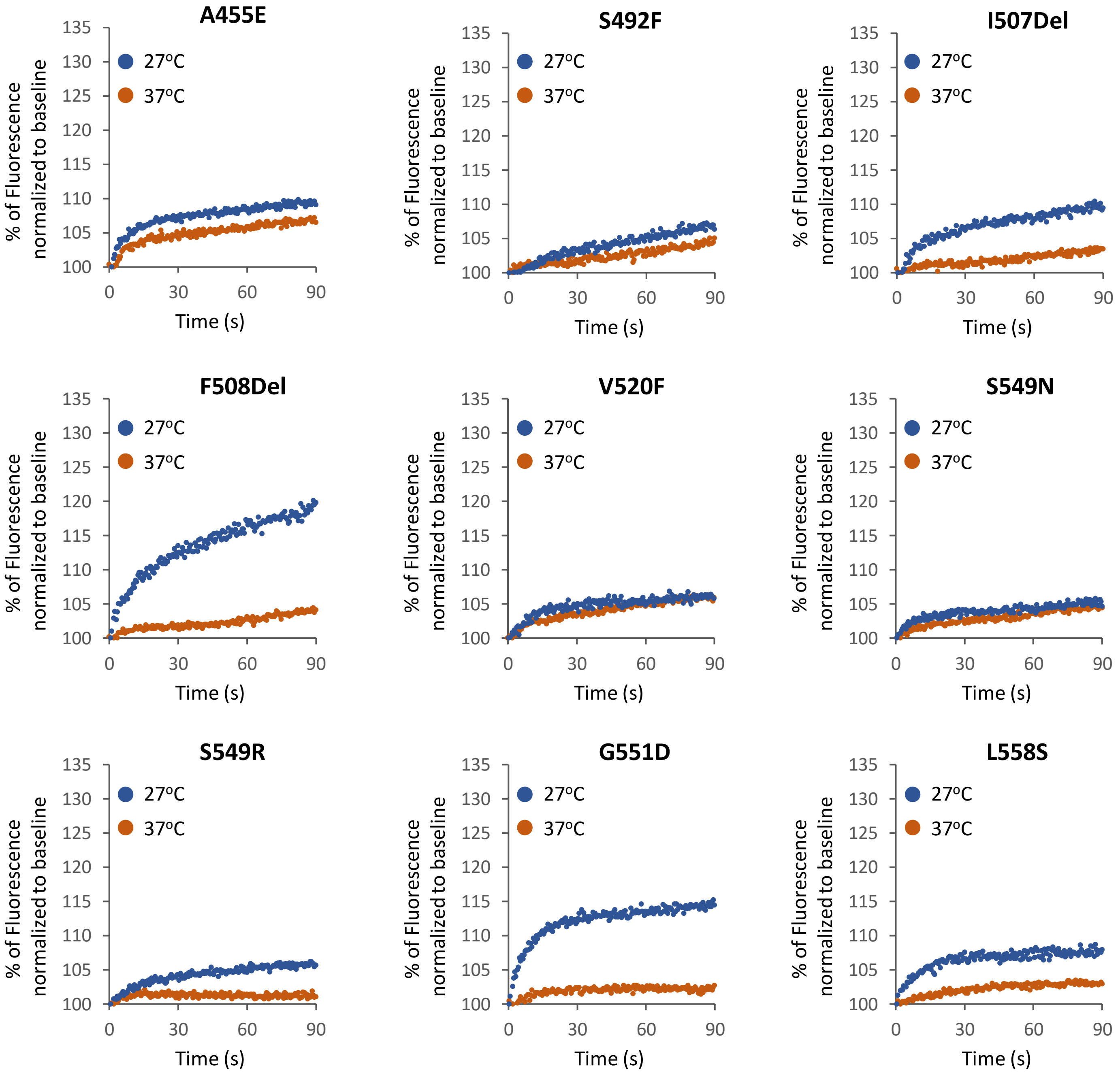

Supplementary Figure 12

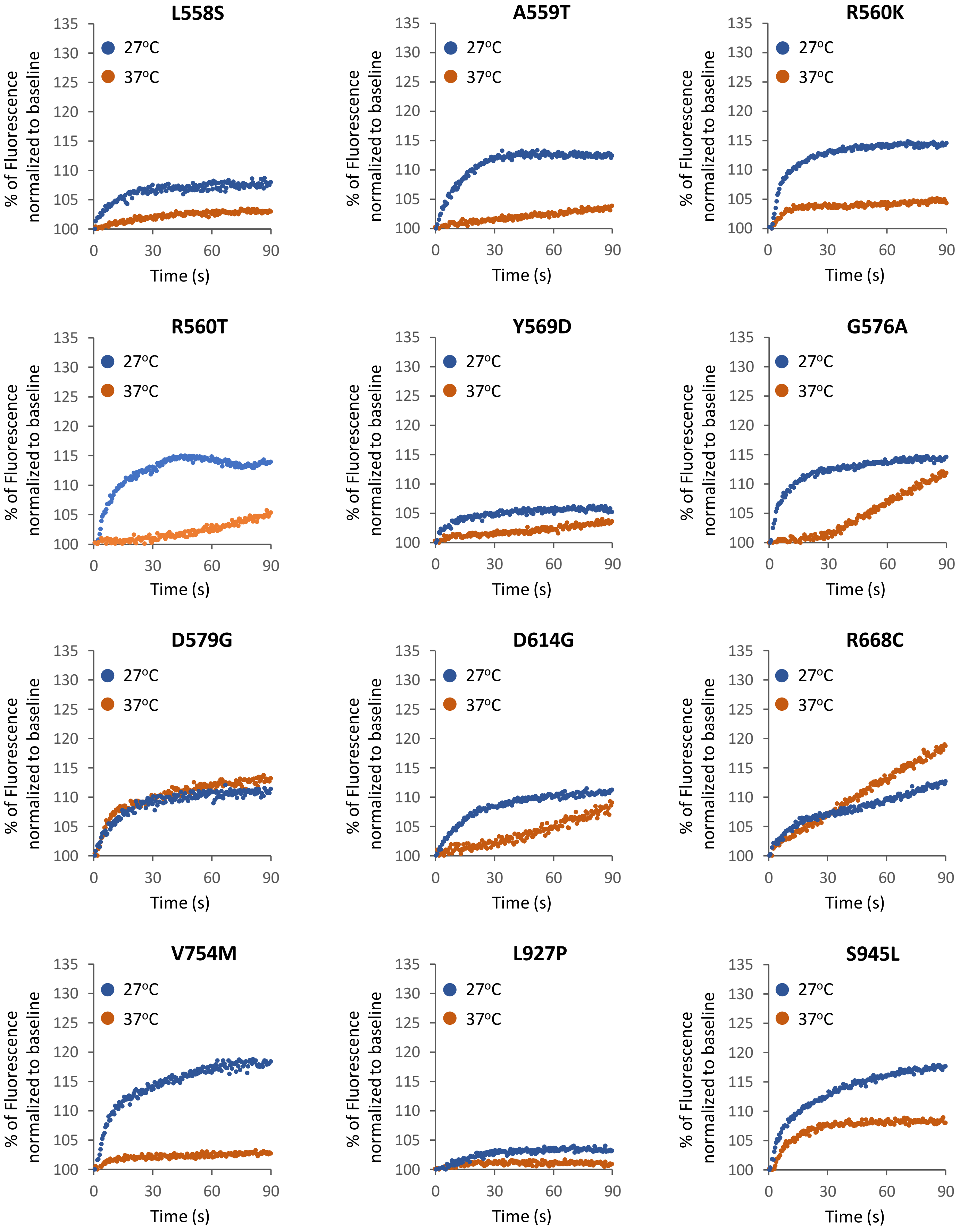

Supplementary Figure 13

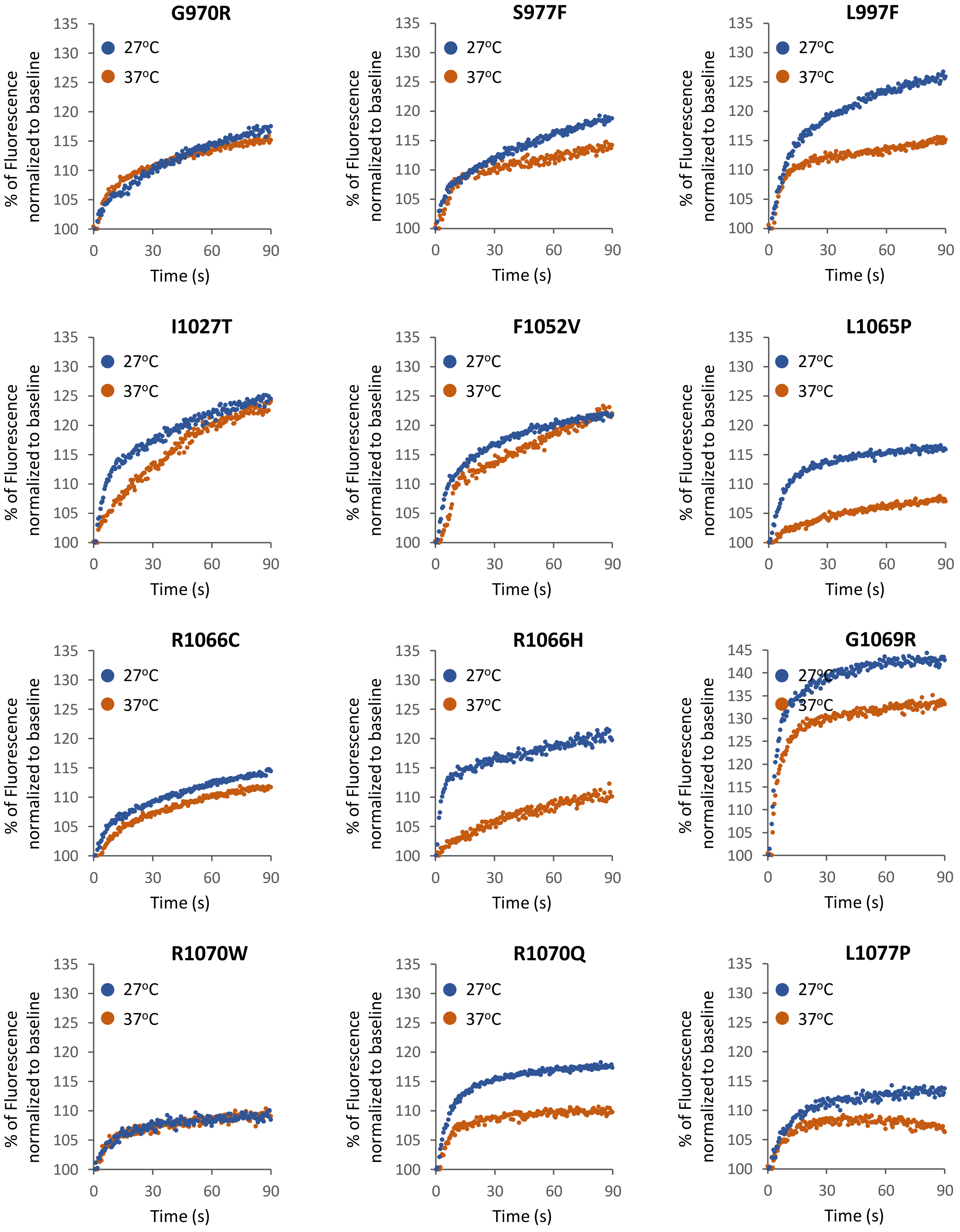

Supplementary Figure 14

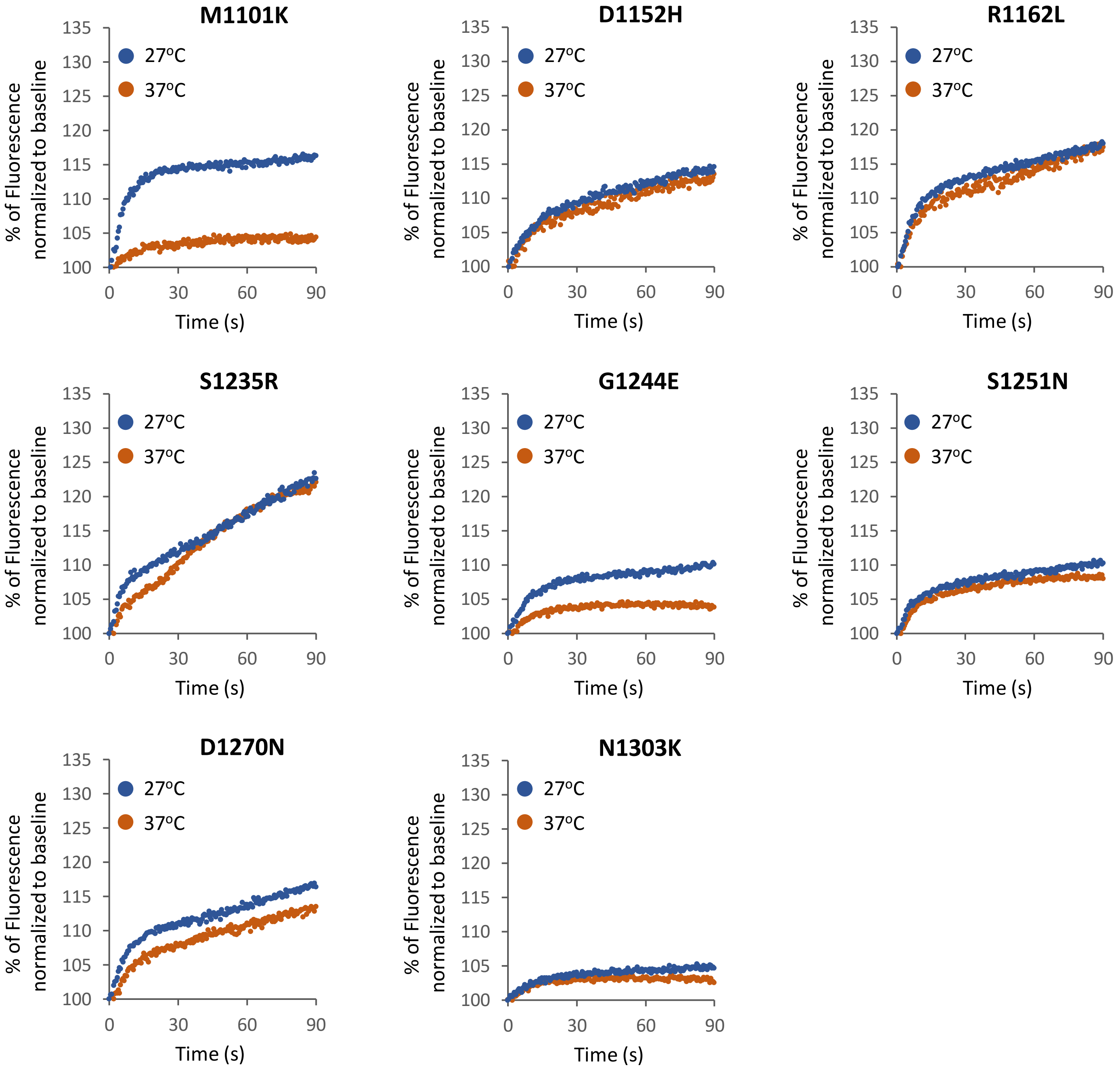

Supplementary Figure 15

a

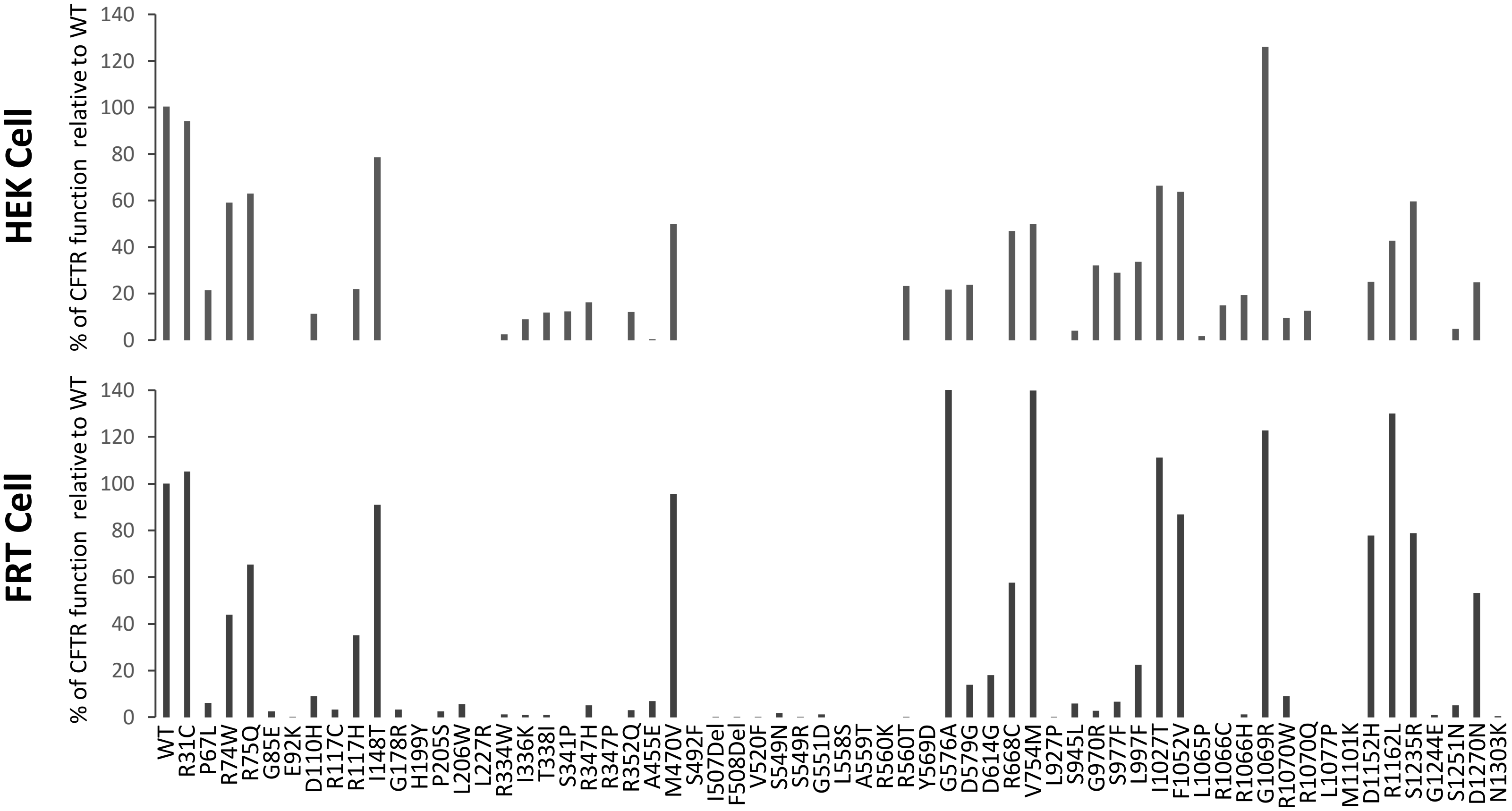

b

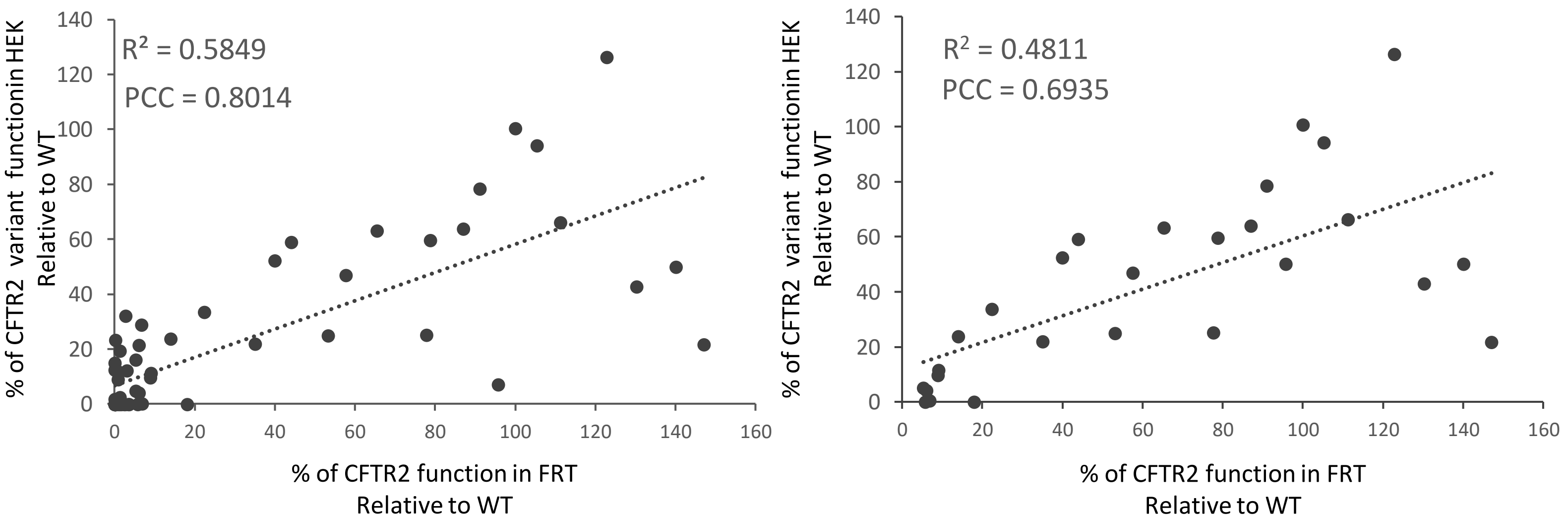

Supplementary Figure 16

a

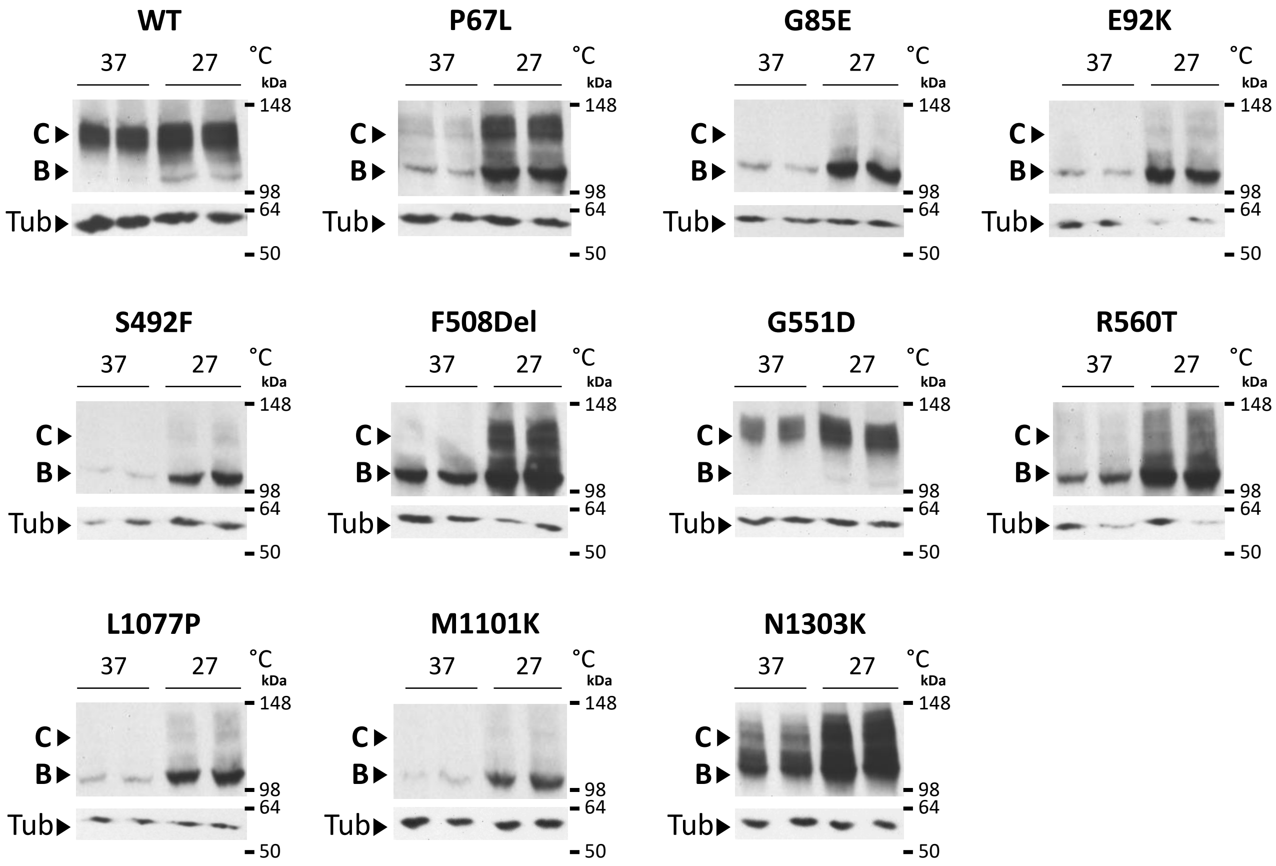

b

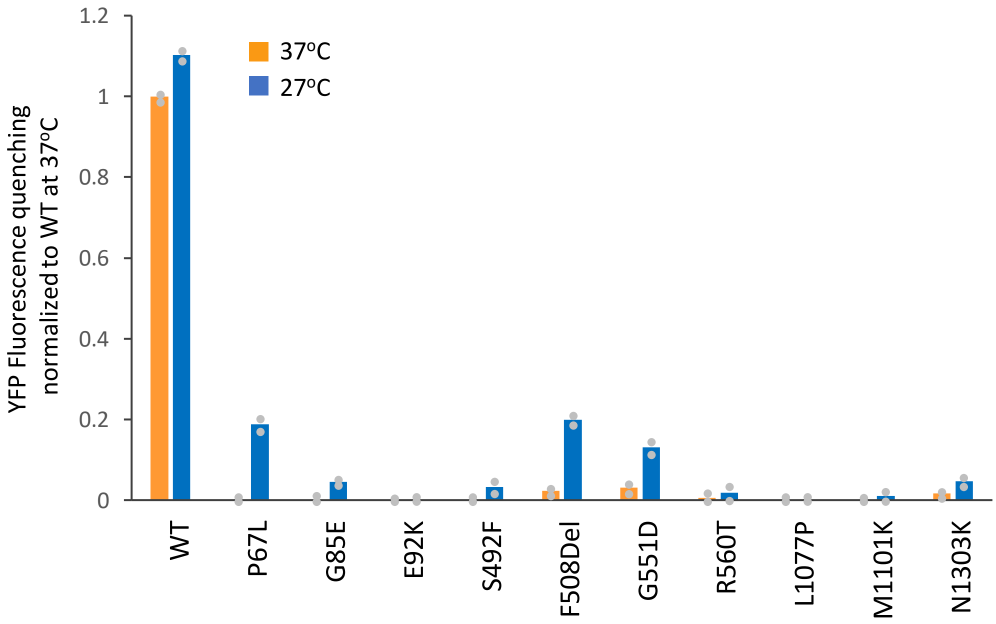

Supplementary Figure 17

a

Variants	position	VarSeq	ClCon		TrIdx	
			Measured	Predicted	Measured	Predicted
M1V	1	0.000676	0.00	0.0600	0.06	0.2898
A46D	46	0.031081	0.00	0.0780	0.21	0.1434
E56K	56	0.037838	0.02	0.0820	0.29	0.3128
D110E	110	0.074324	0.11	0.3477	0.92	0.9111
E193K	193	0.130405	0.31	0.1991	0.84	0.8962
L467P	467	0.315541	0.04	0.0177	0.10	0.2801
S549N	549	0.370946	0.00	0.0443	0.90	0.9393
S549R	549	0.370946	0.01	0.0530	0.70	0.5923
G551S	551	0.372297	0.03	0.0490	0.90	0.7881
R560S	560	0.378378	0.00	0.0000	0.00	0.2749
A561E	561	0.379054	0.00	0.0000	0.08	0.5122
L927P	927	0.626351	0.00	0.0429	0.89	0.8047
H1054D	1054	0.712162	0.04	0.0295	0.09	0.2851
K1060T	1060	0.716216	0.45	0.4125	0.87	0.8894
R1066M	1066	0.72027	0.04	0.0000	0.02	0.1987
A1067T	1067	0.720946	0.30	0.0700	0.78	0.9239
F1074L	1074	0.725676	0.09	0.0489	0.52	0.4887
H1085R	1085	0.733108	0.04	0.0747	0.06	0.3074
S1251N	1251	0.84527	0.01	0.1637	0.90	0.8777
S1255P	1255	0.847973	0.05	0.1384	0.90	0.8575
G1349D	1349	0.911486	0.05	0.1445	0.90	0.6884

b

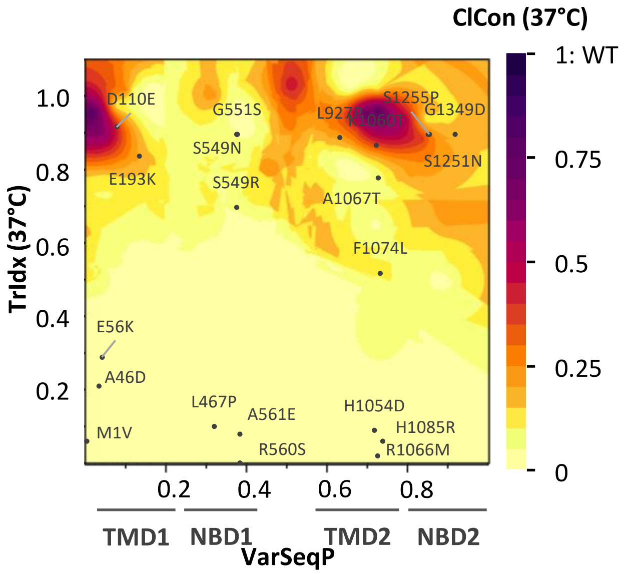

c

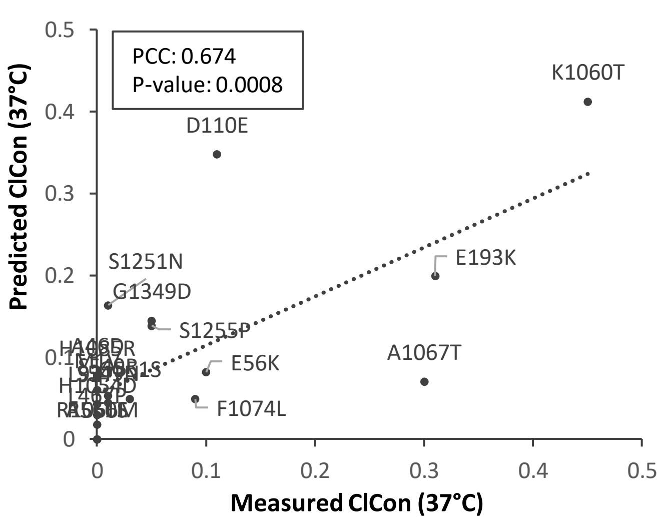

d

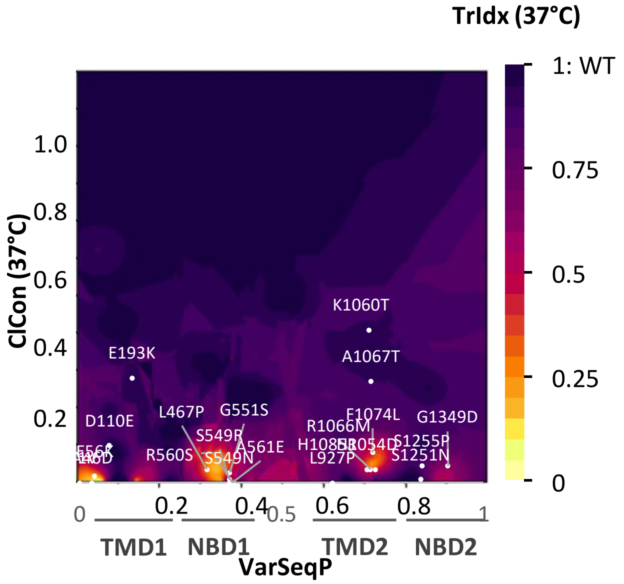

e

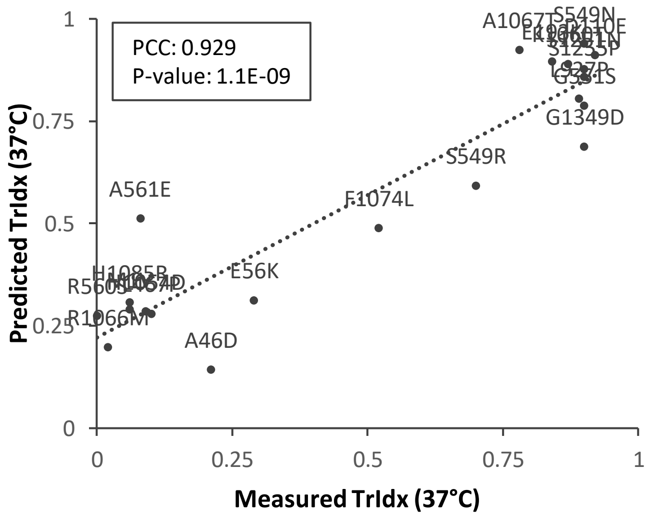

Supplementary Figure 18

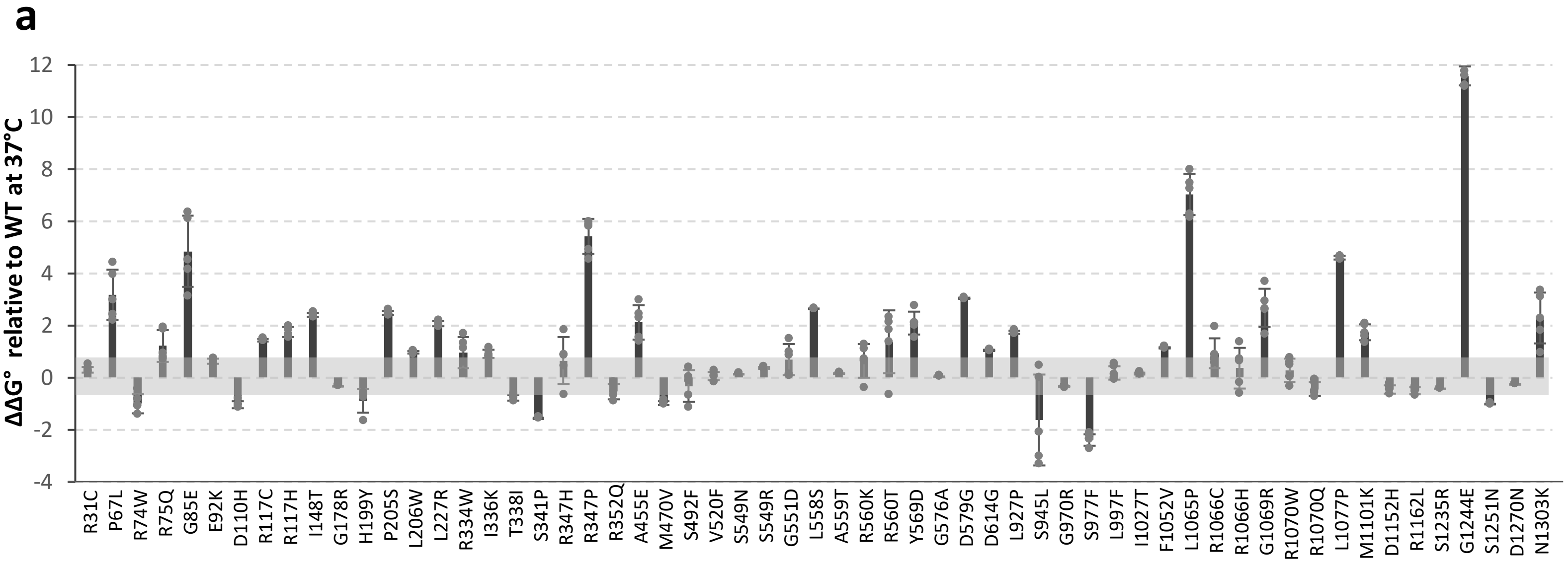

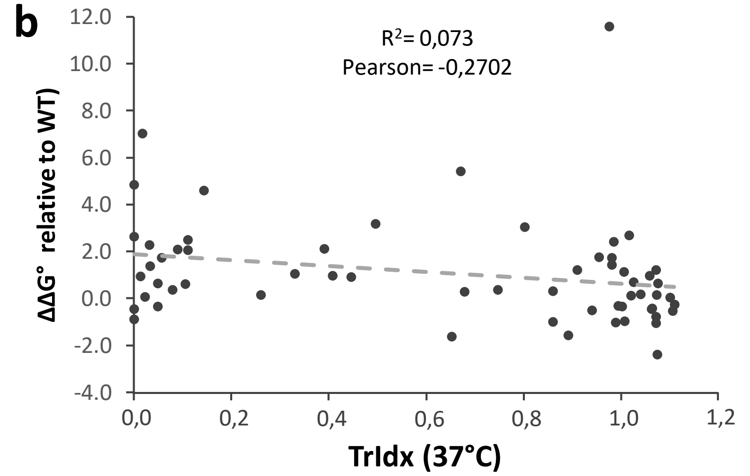

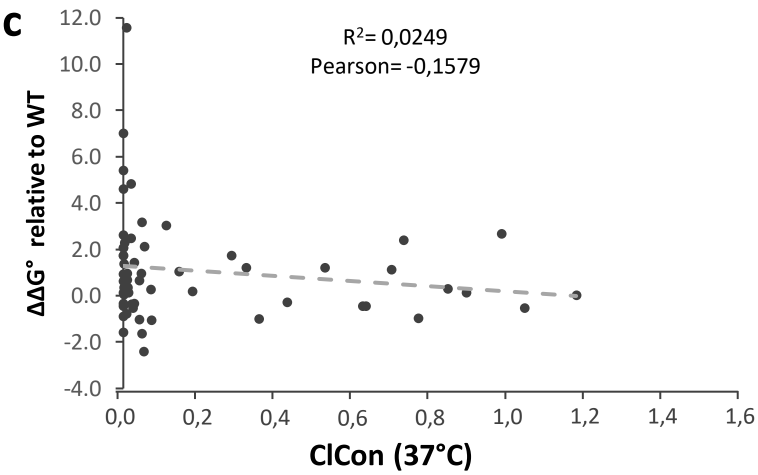

Supplementary Figure 19

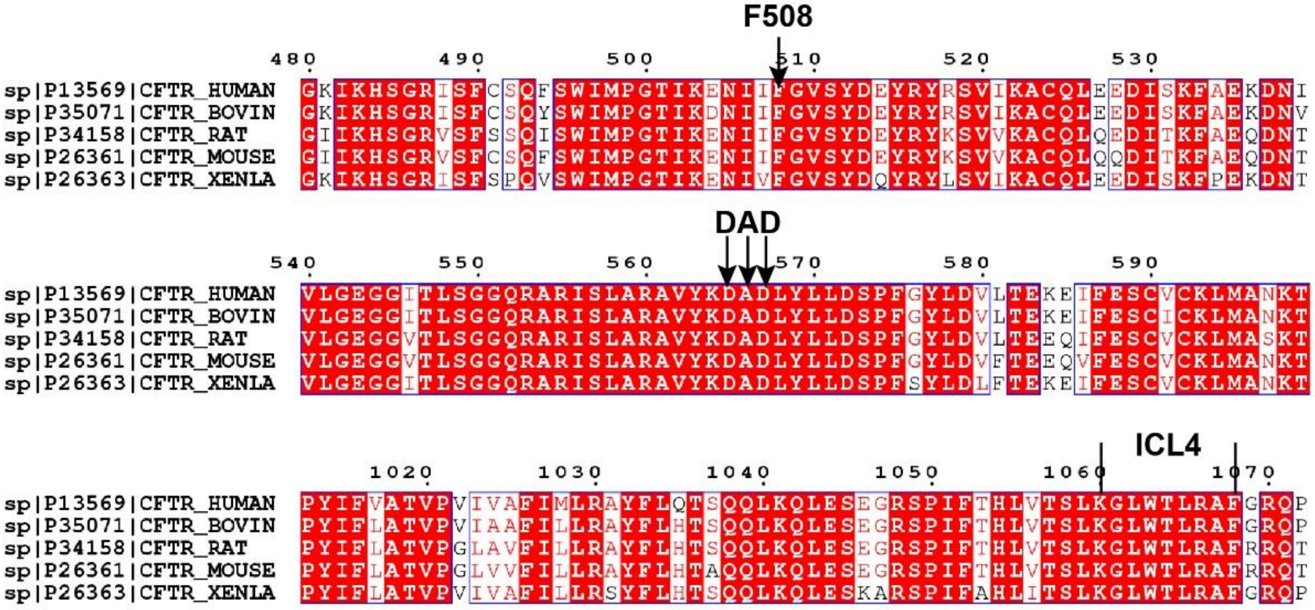

Supplementary Figure 20

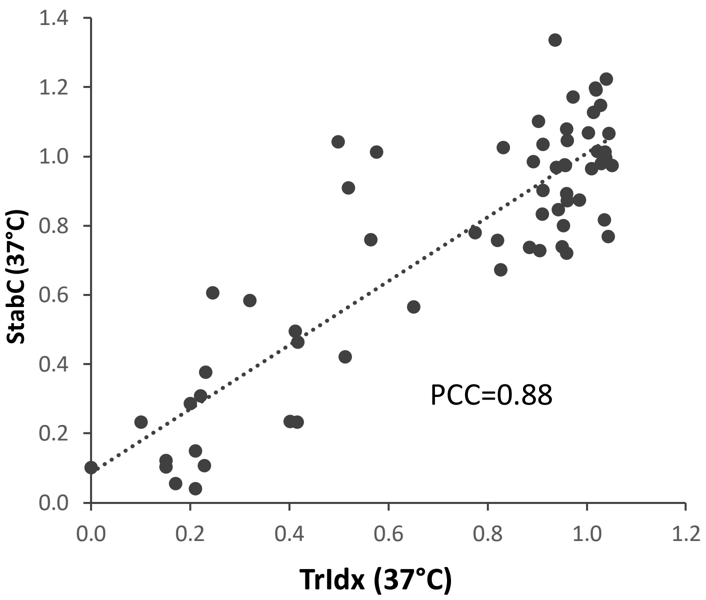

Supplementary Figure 21

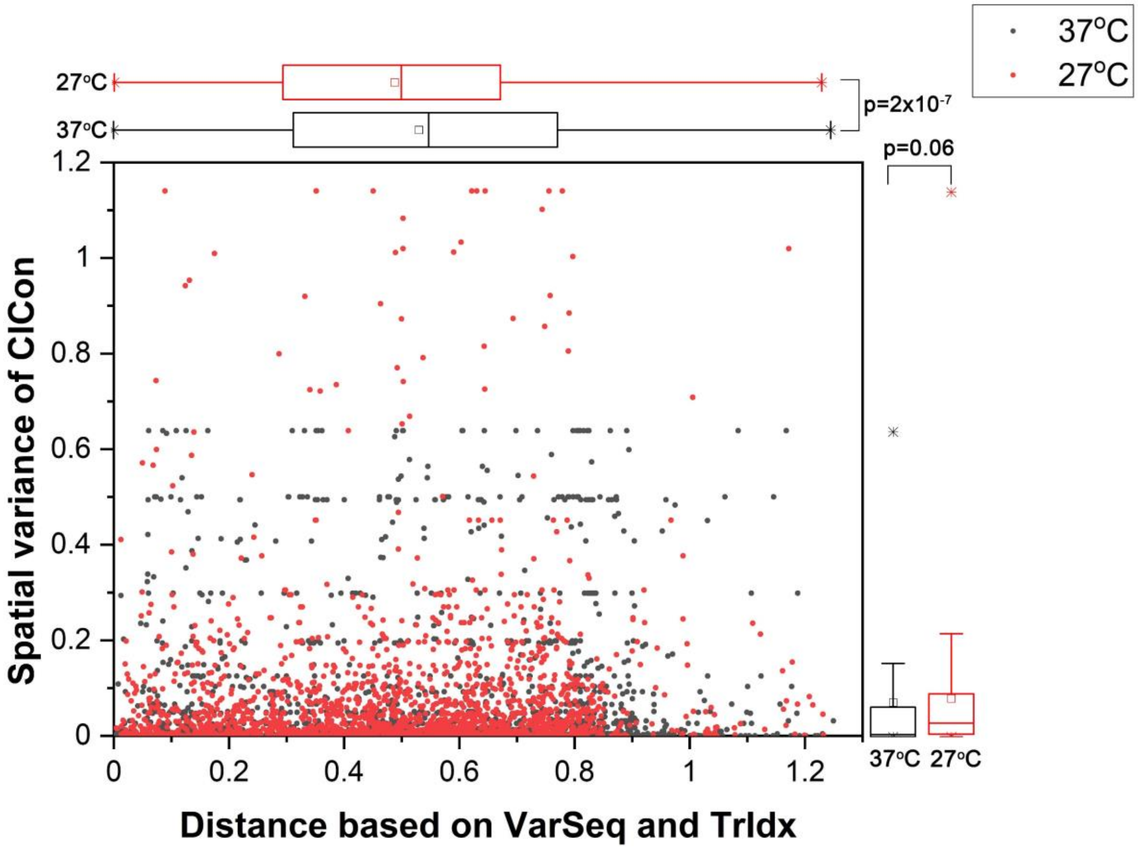

Supplementary Figure 22

a

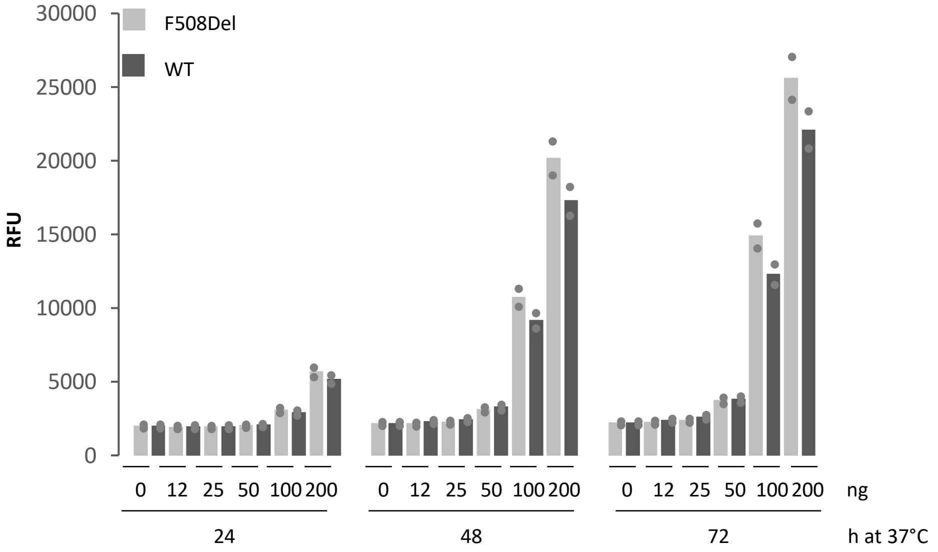

b

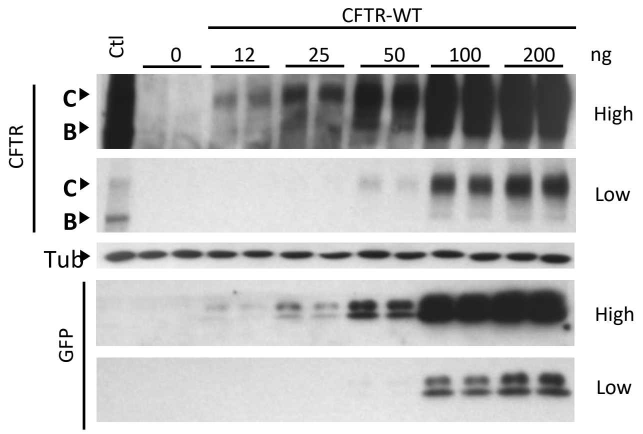

c

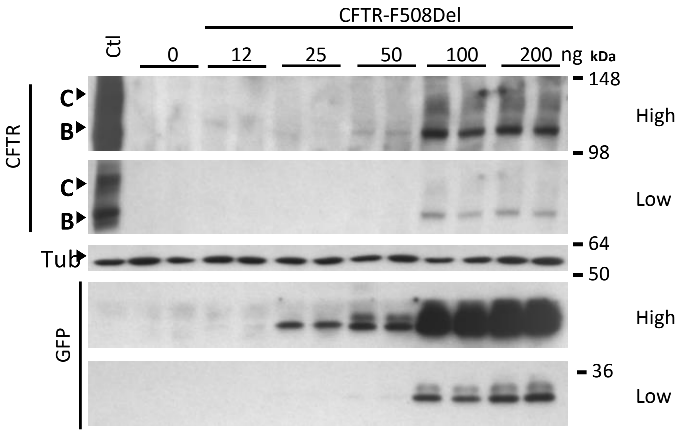

Supplementary Figure 23

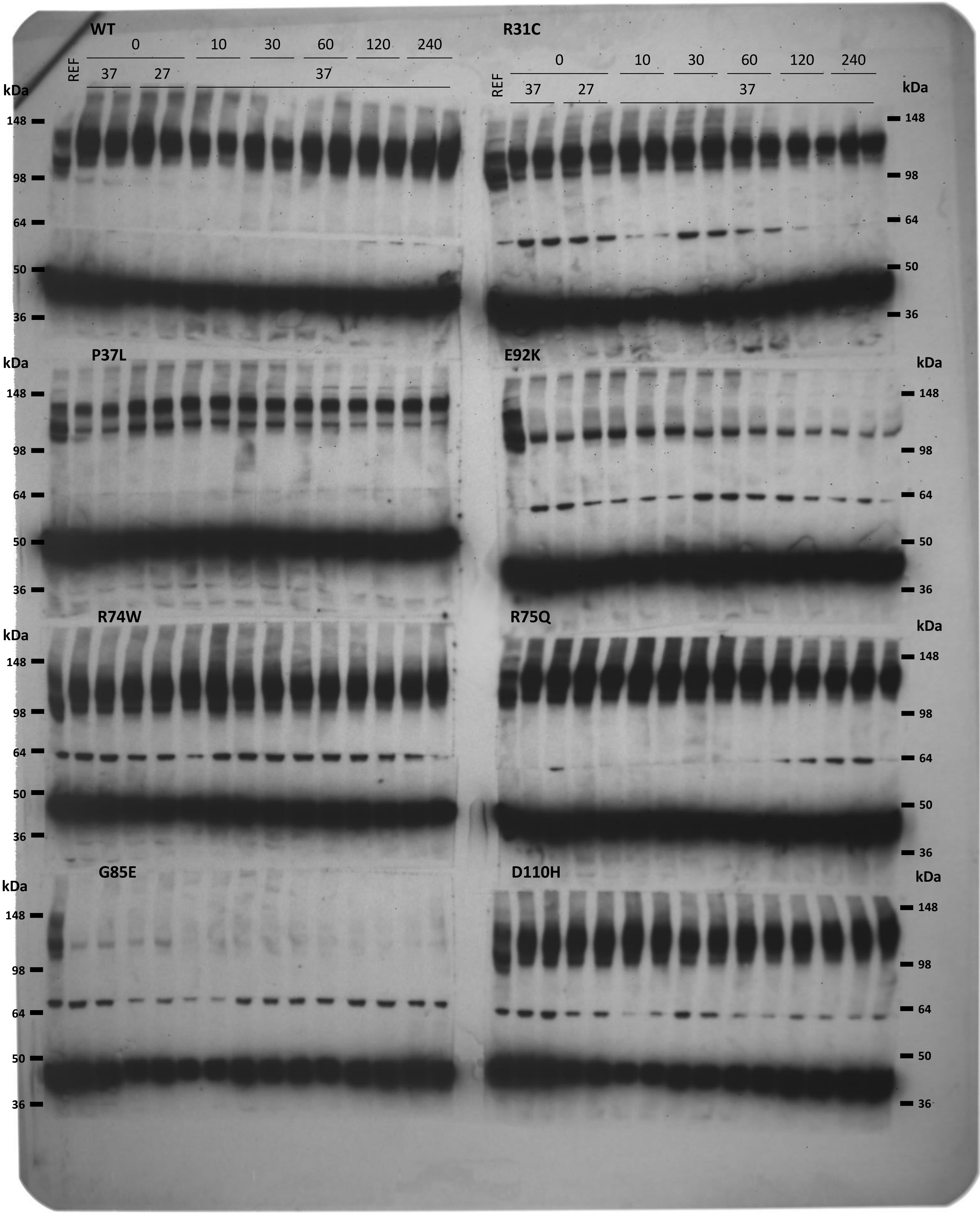

Supplementary Figure 24

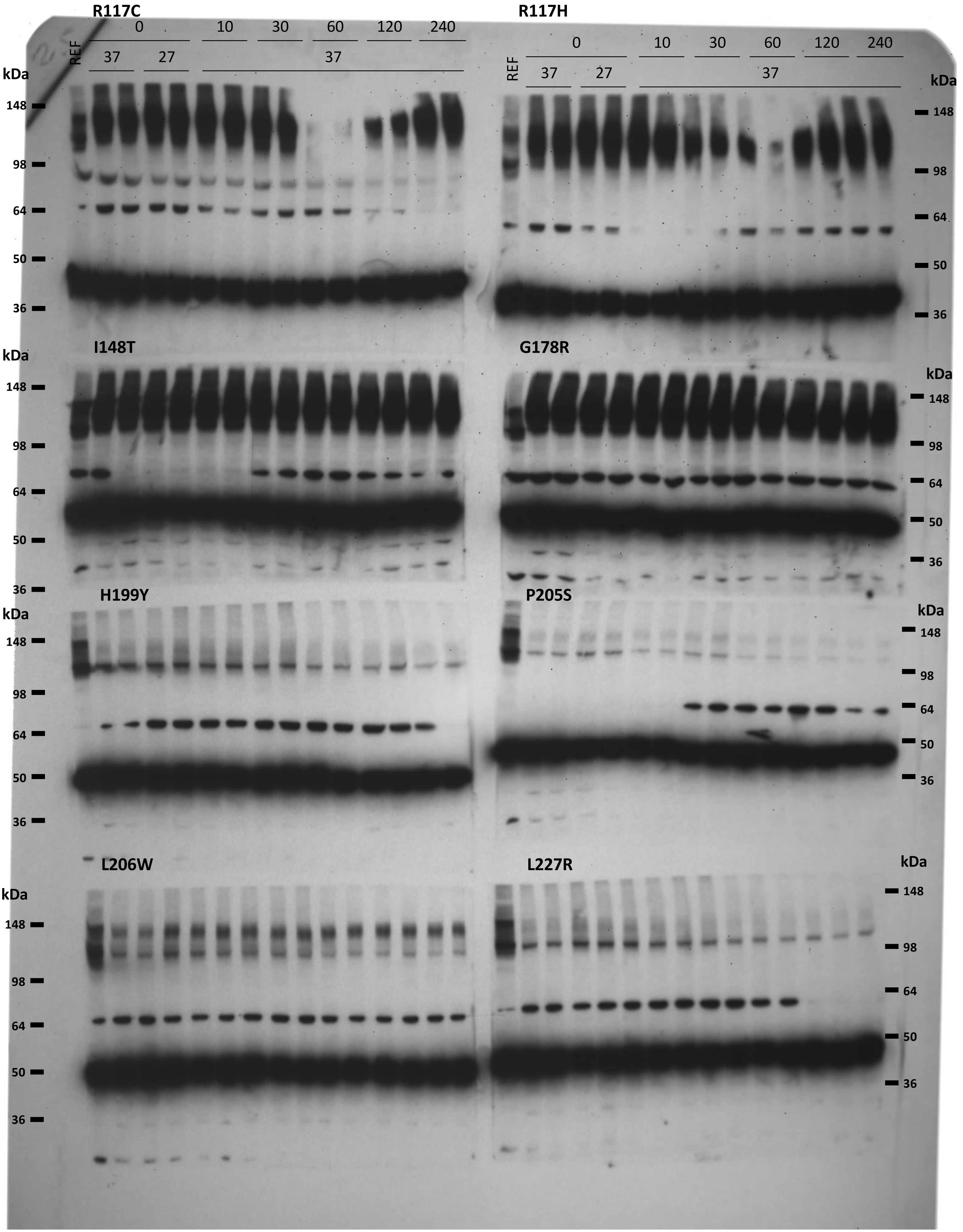

Supplementary Figure 25

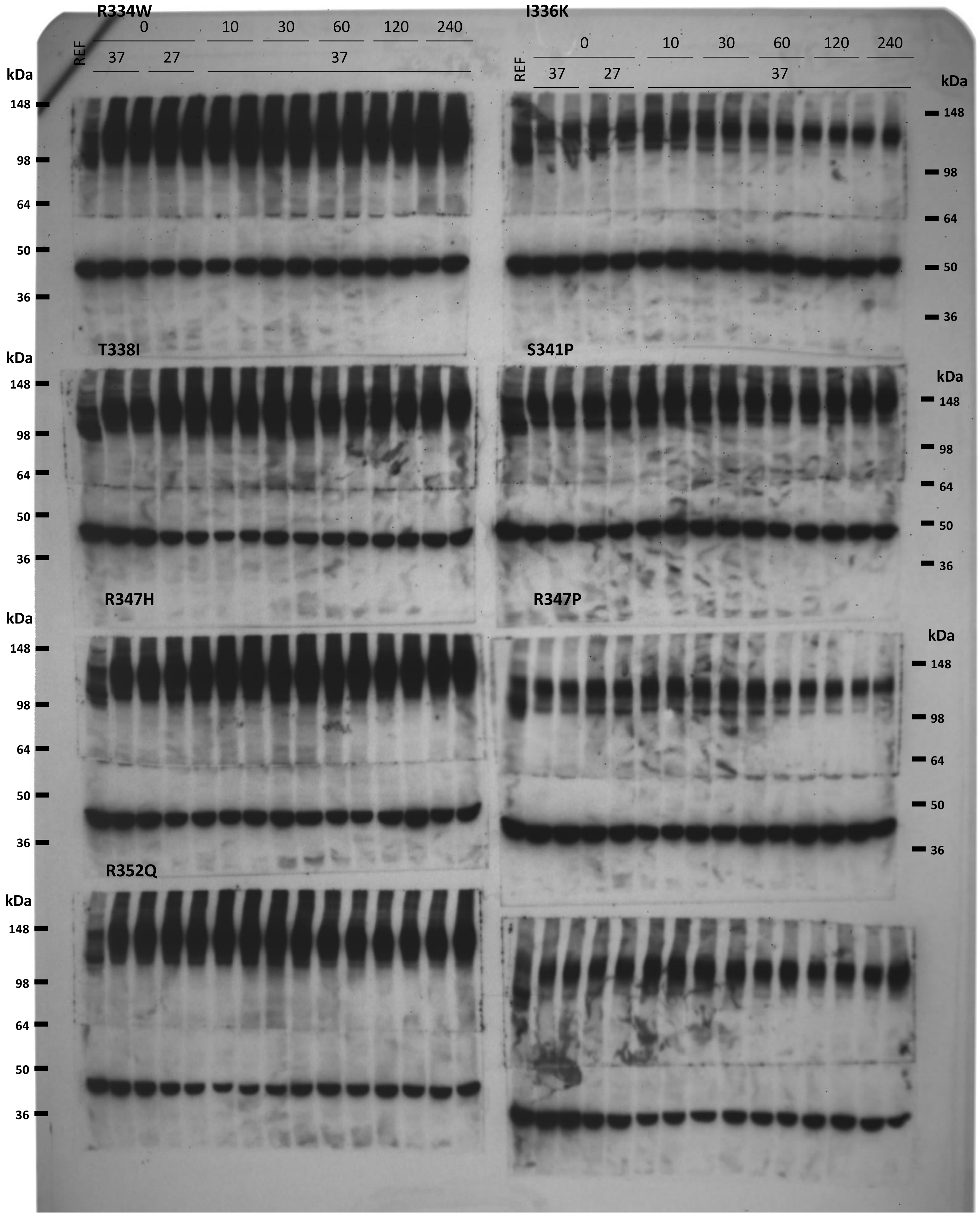

Supplementary Figure 26

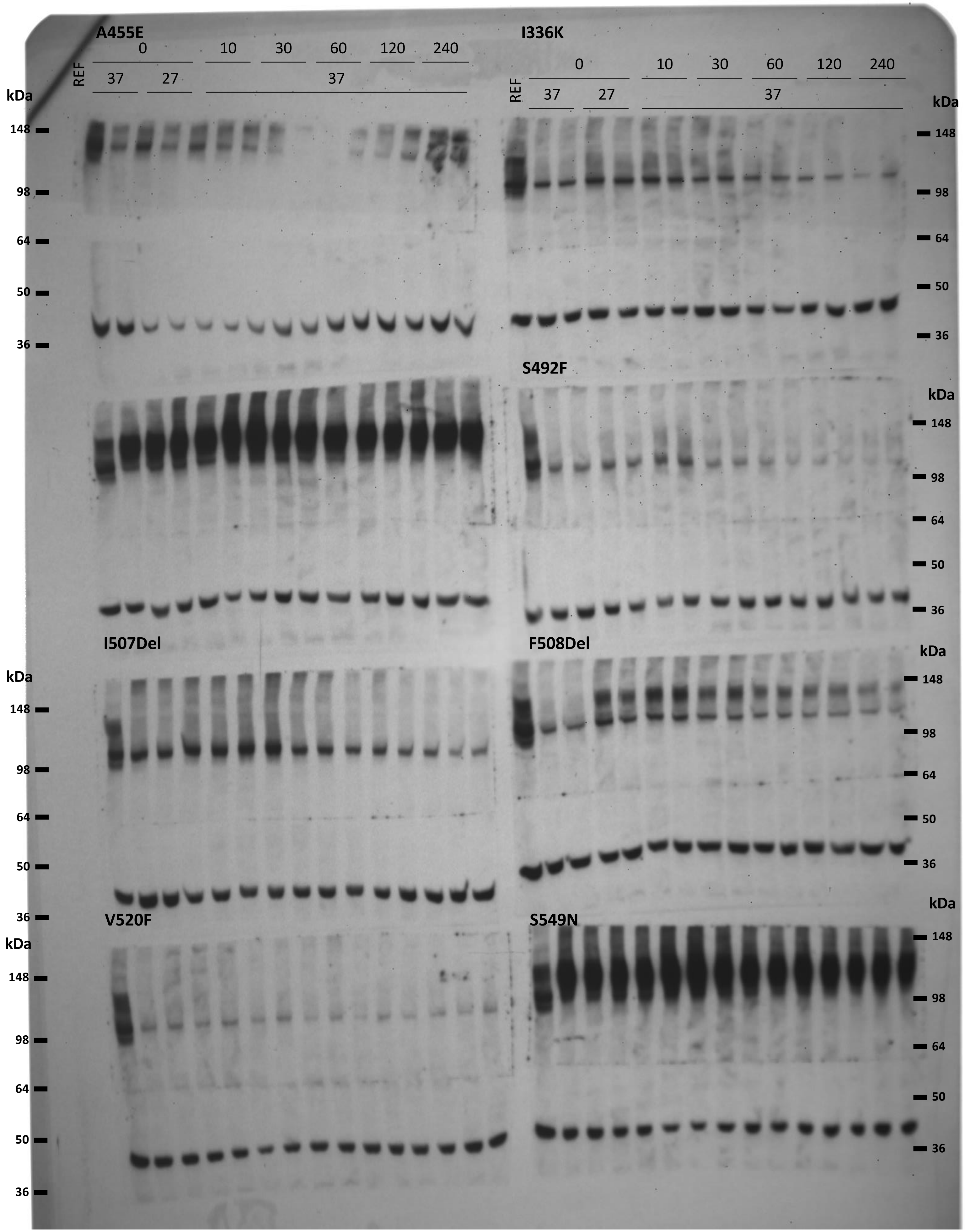

Supplementary Figure 27

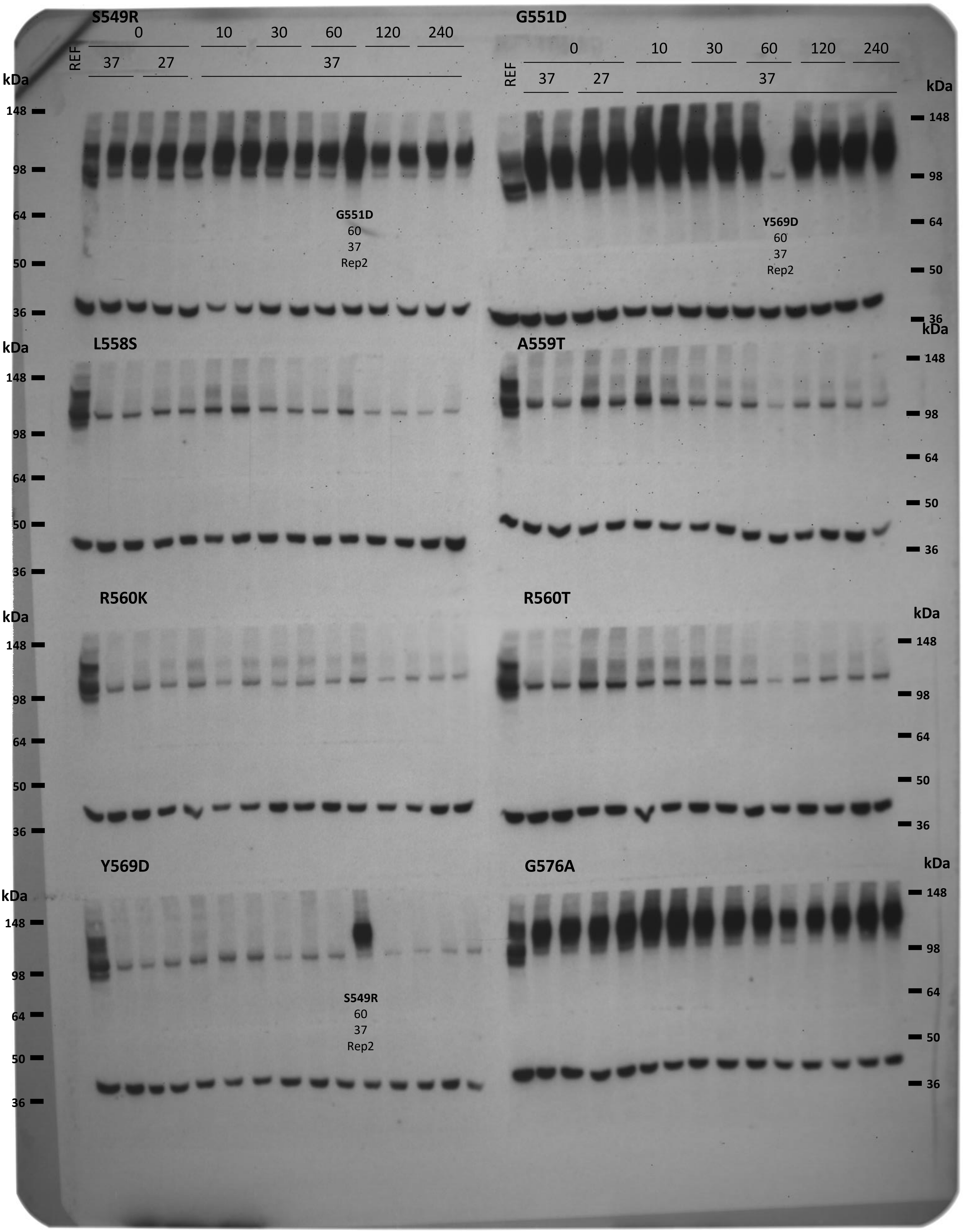

Supplementary Figure 28

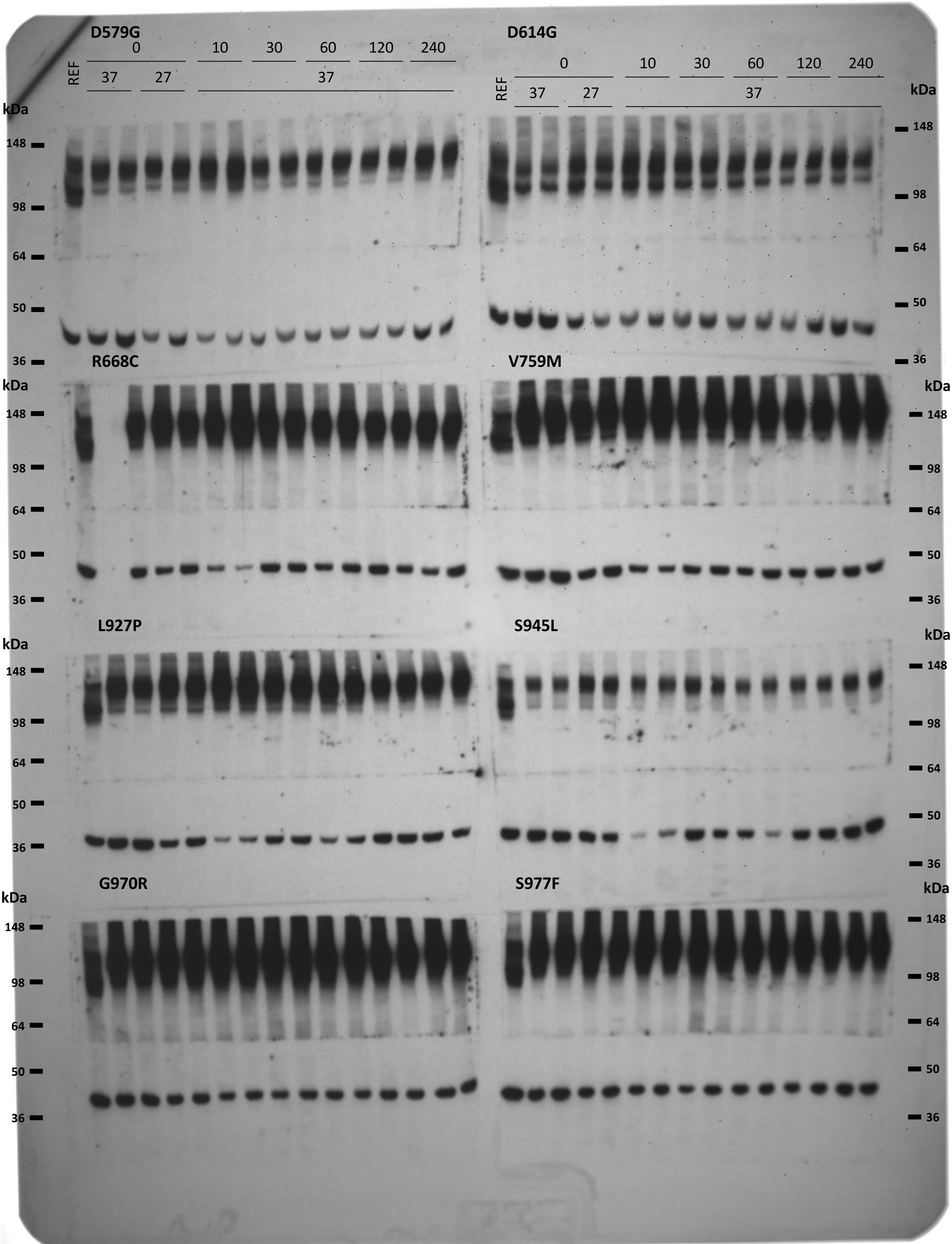

Supplementary Figure 29

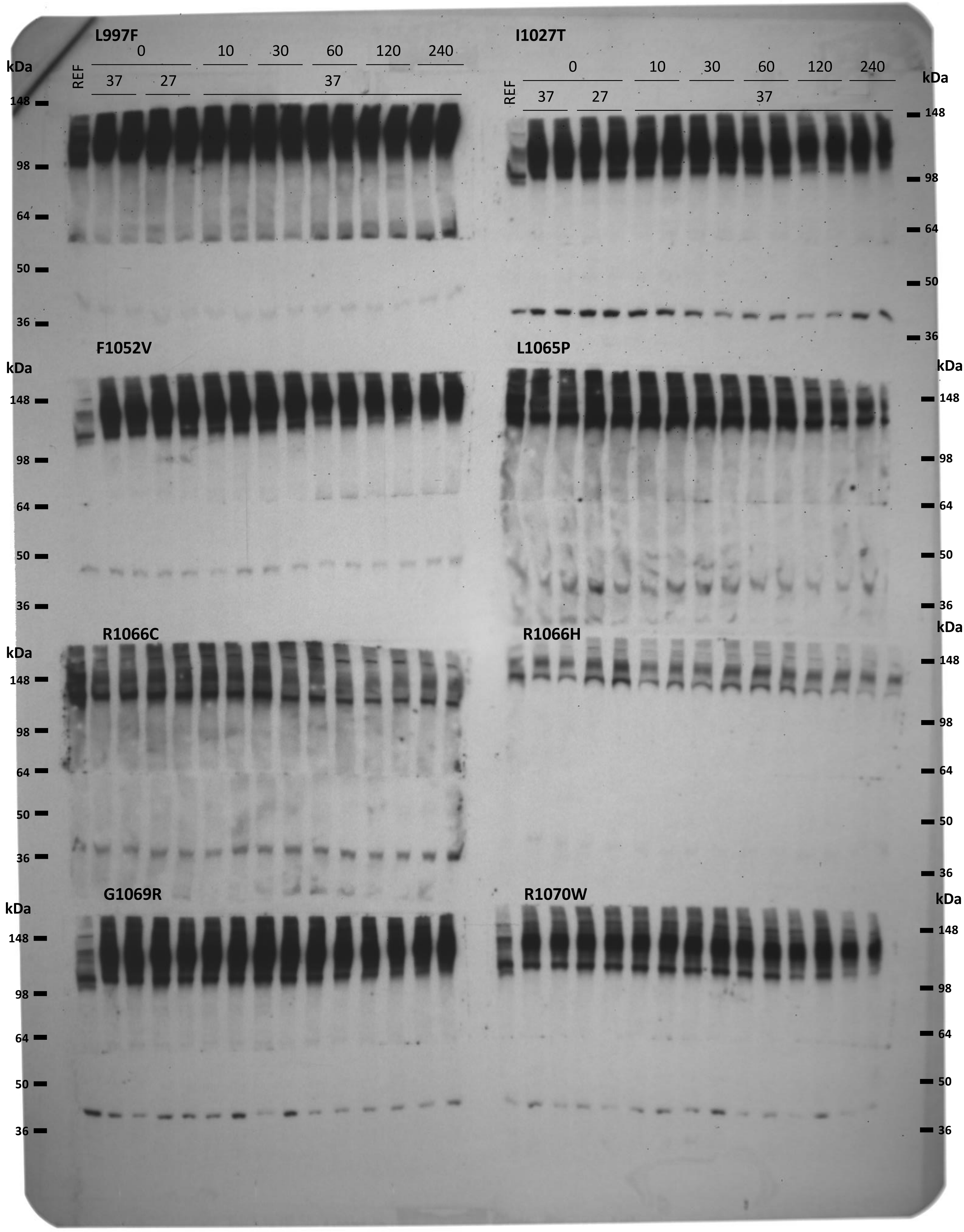

Supplementary Figure 30

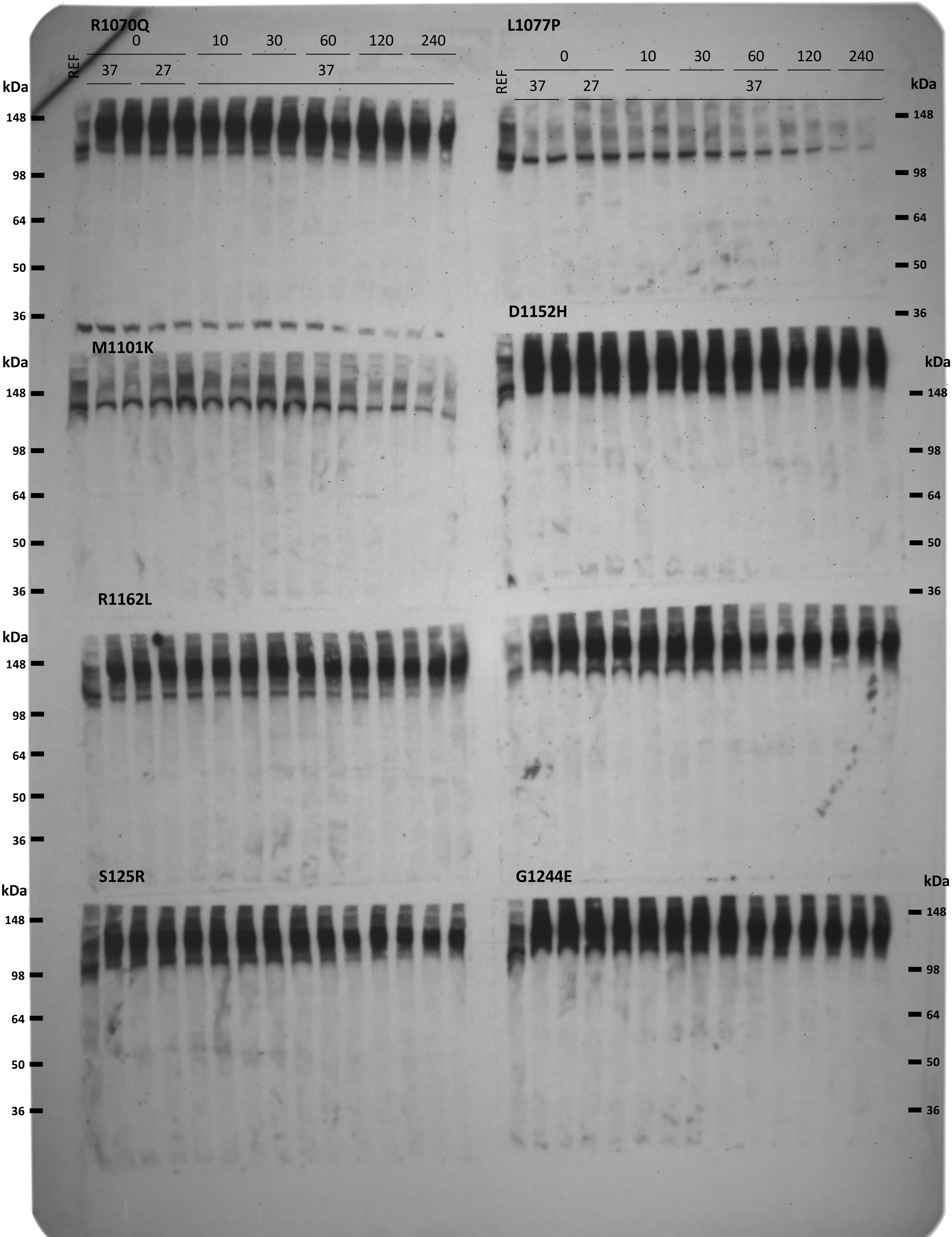

Supplementary Figure 31

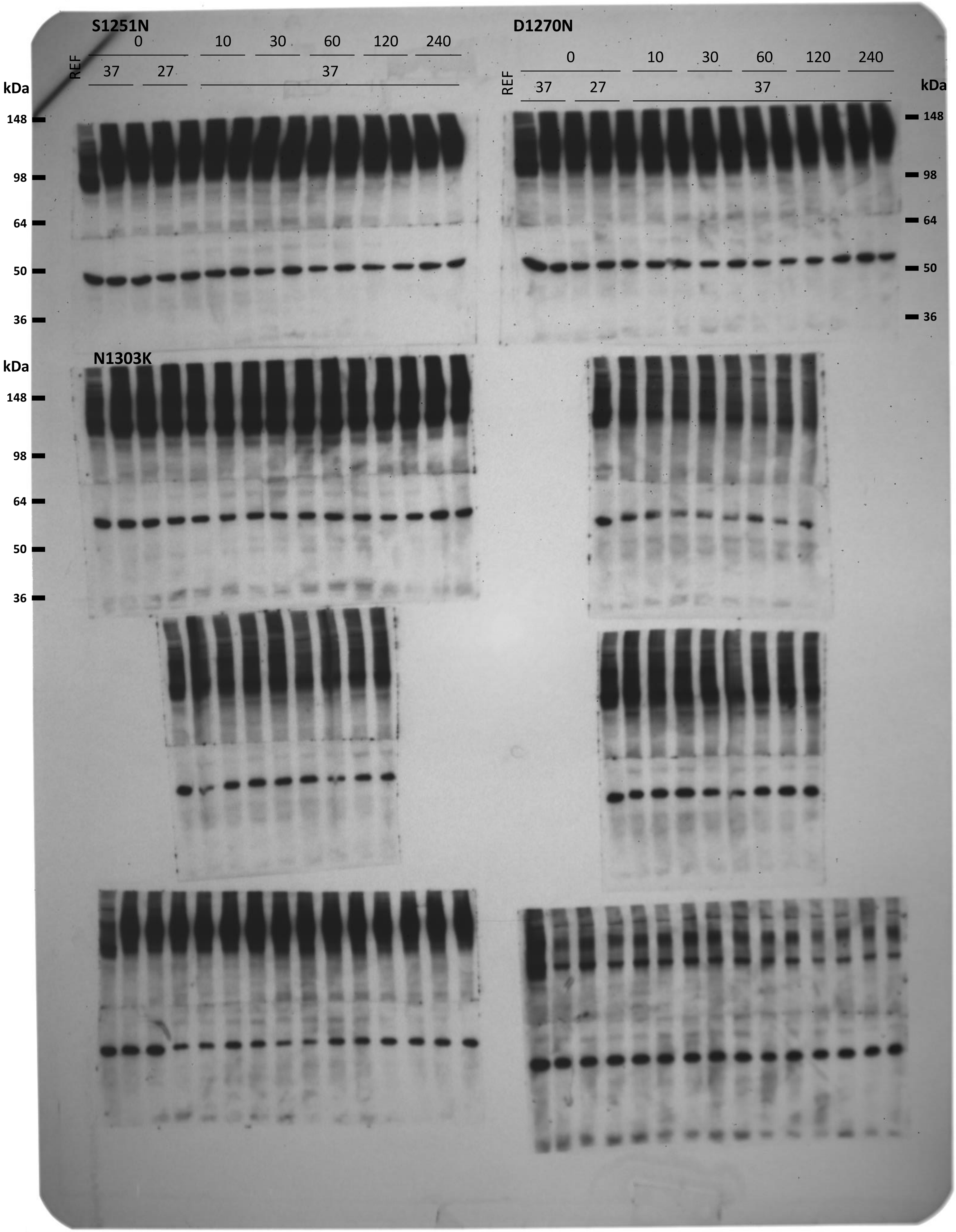

Supplementary Figure 32

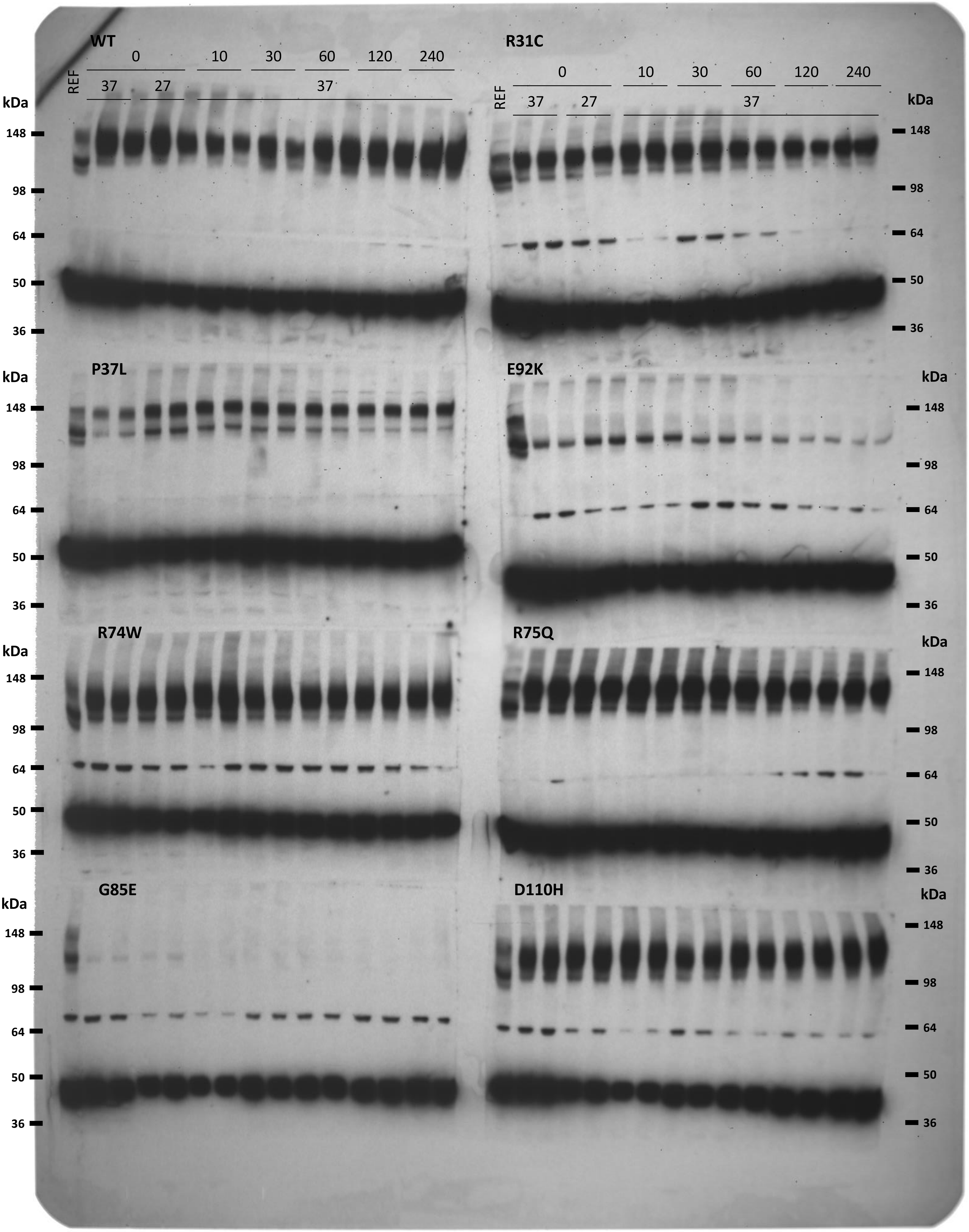

Supplementary Figure 33

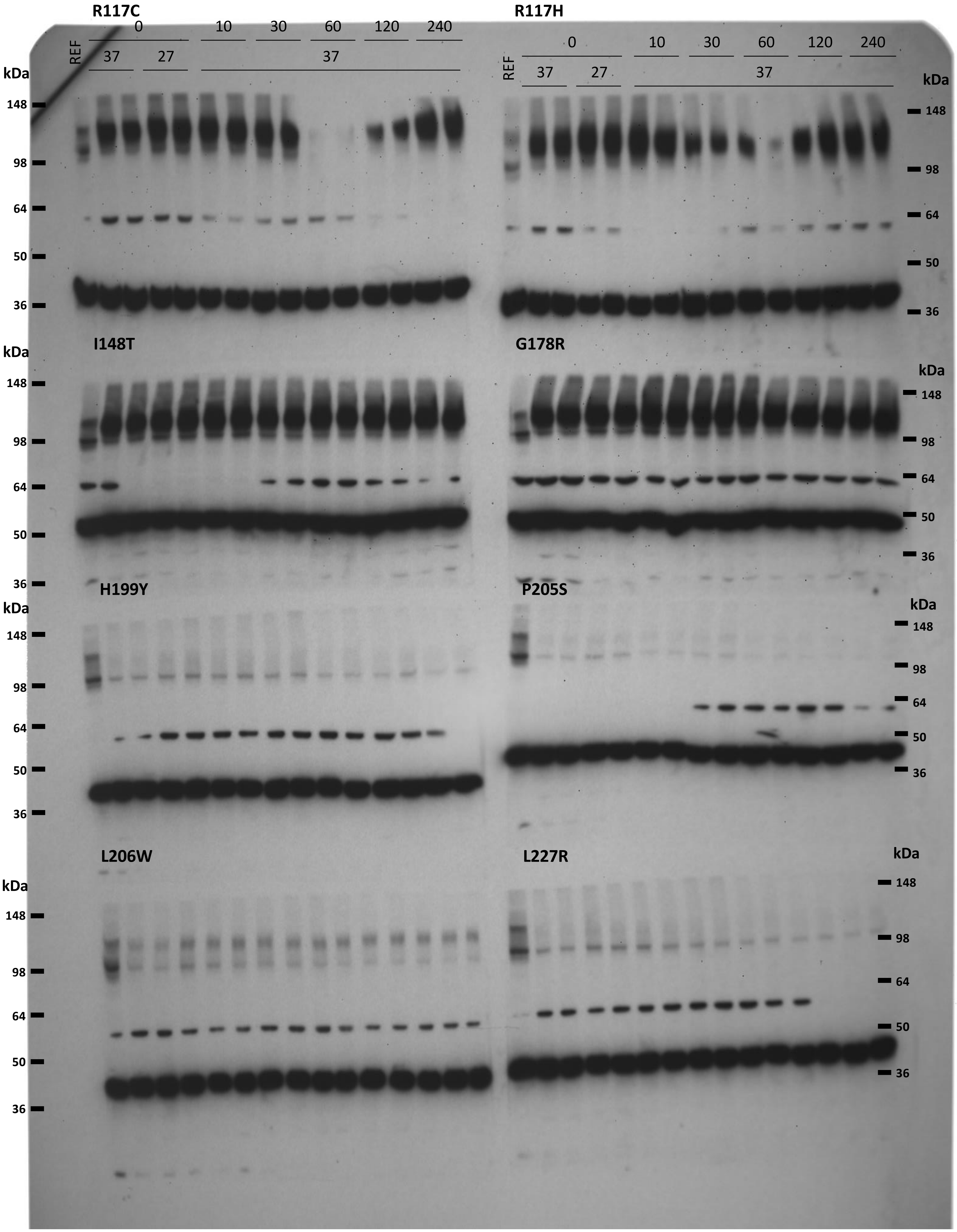

Supplementary Figure 34

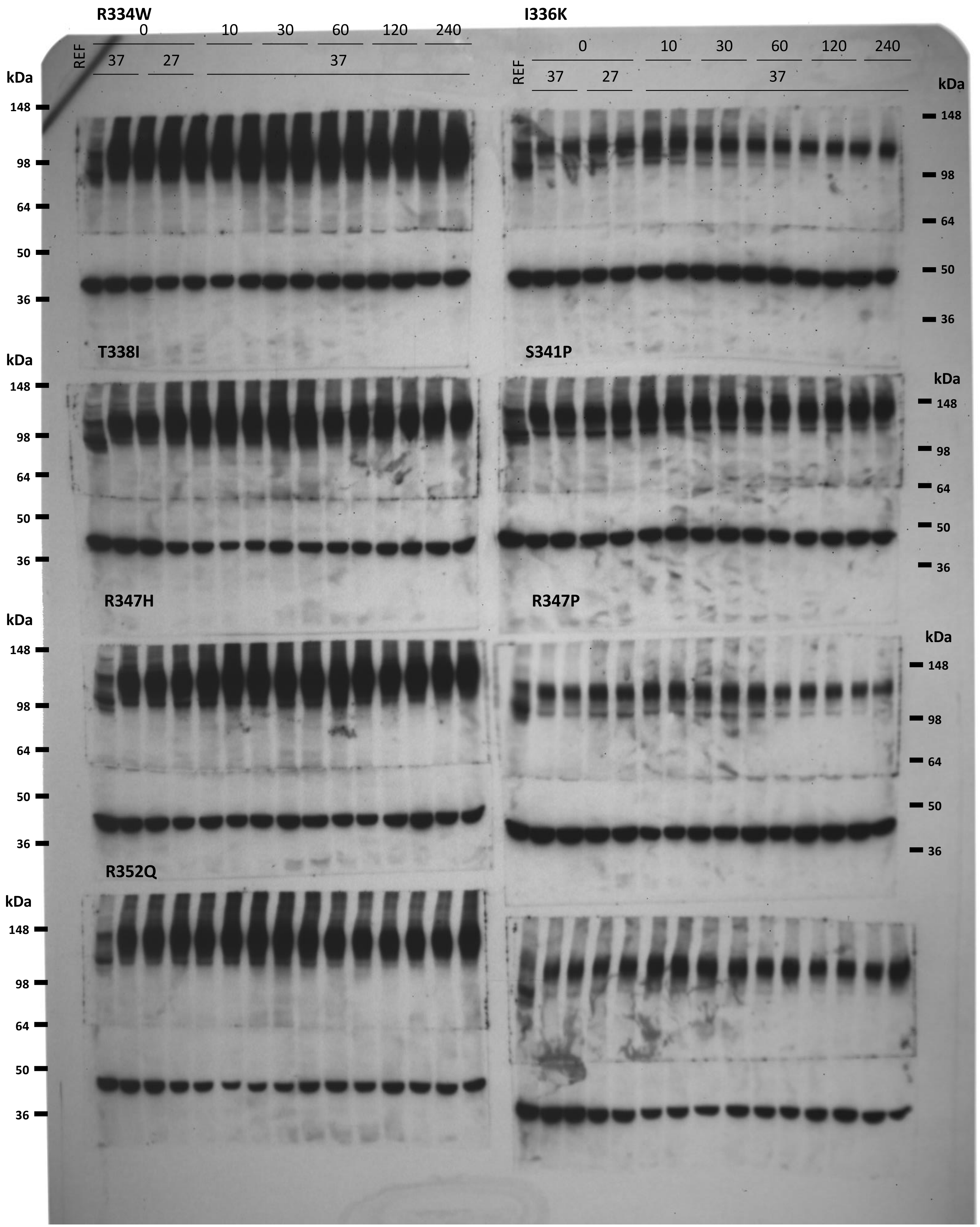

Supplementary Figure 35

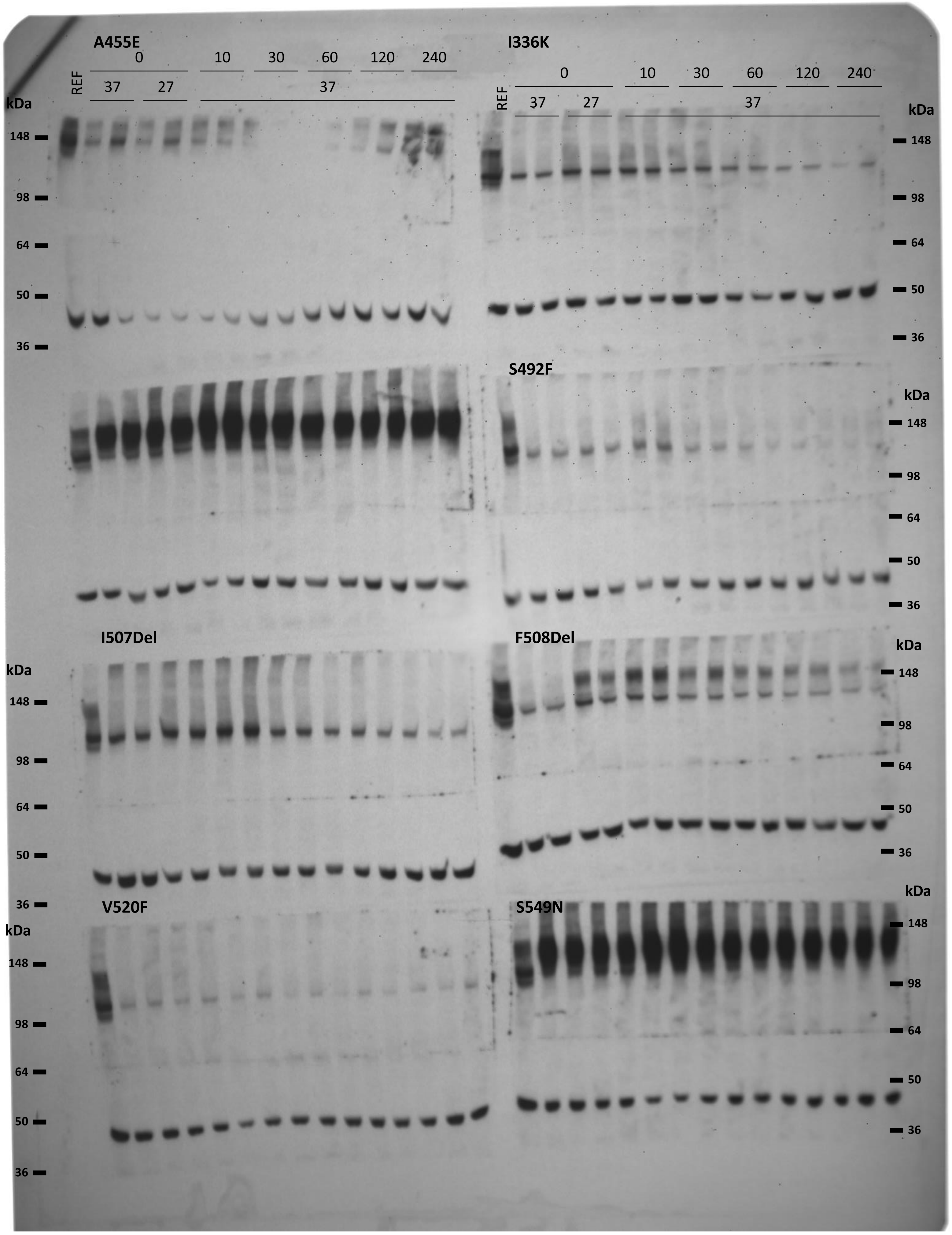

Supplementary Figure 36

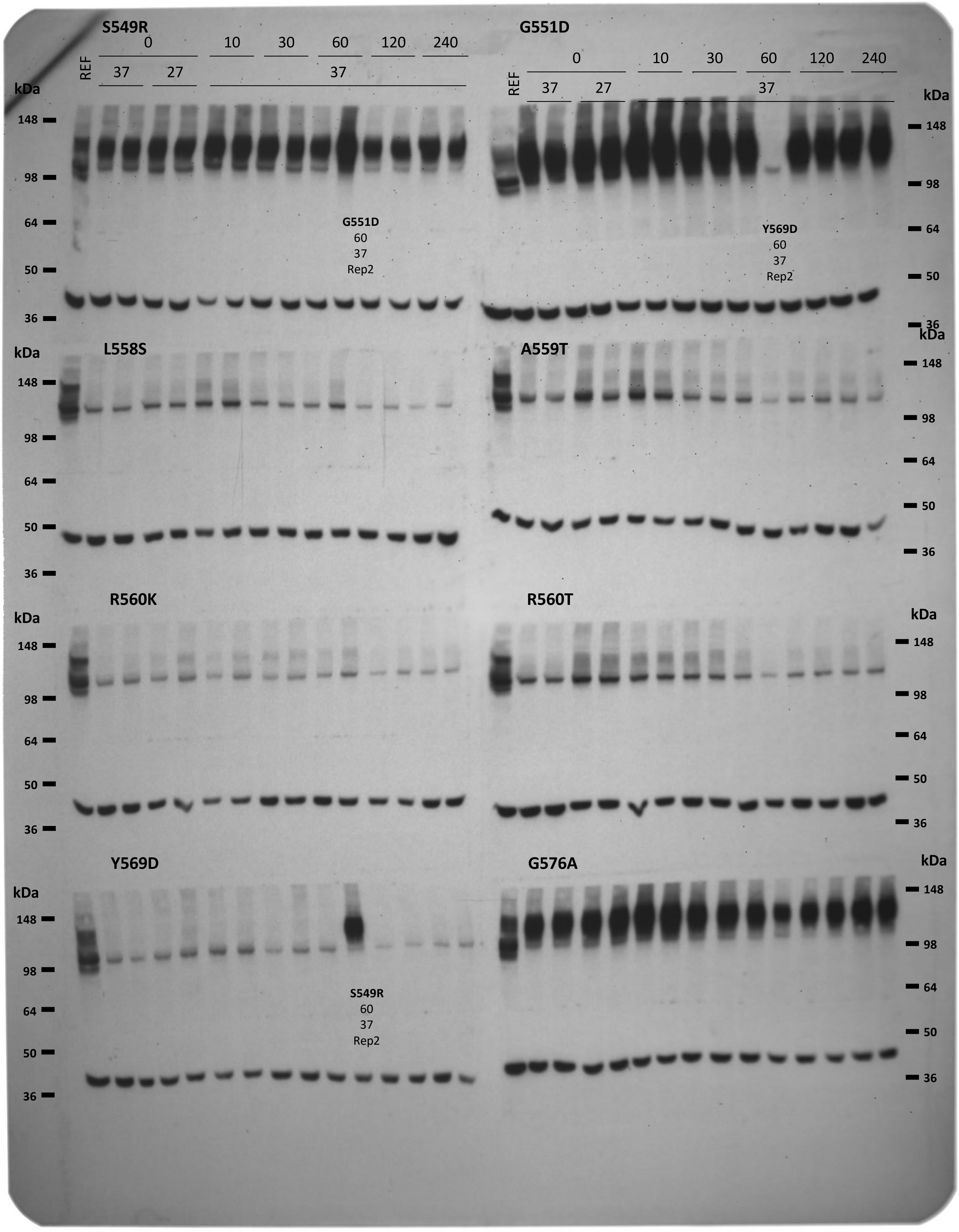

Supplementary Figure 37

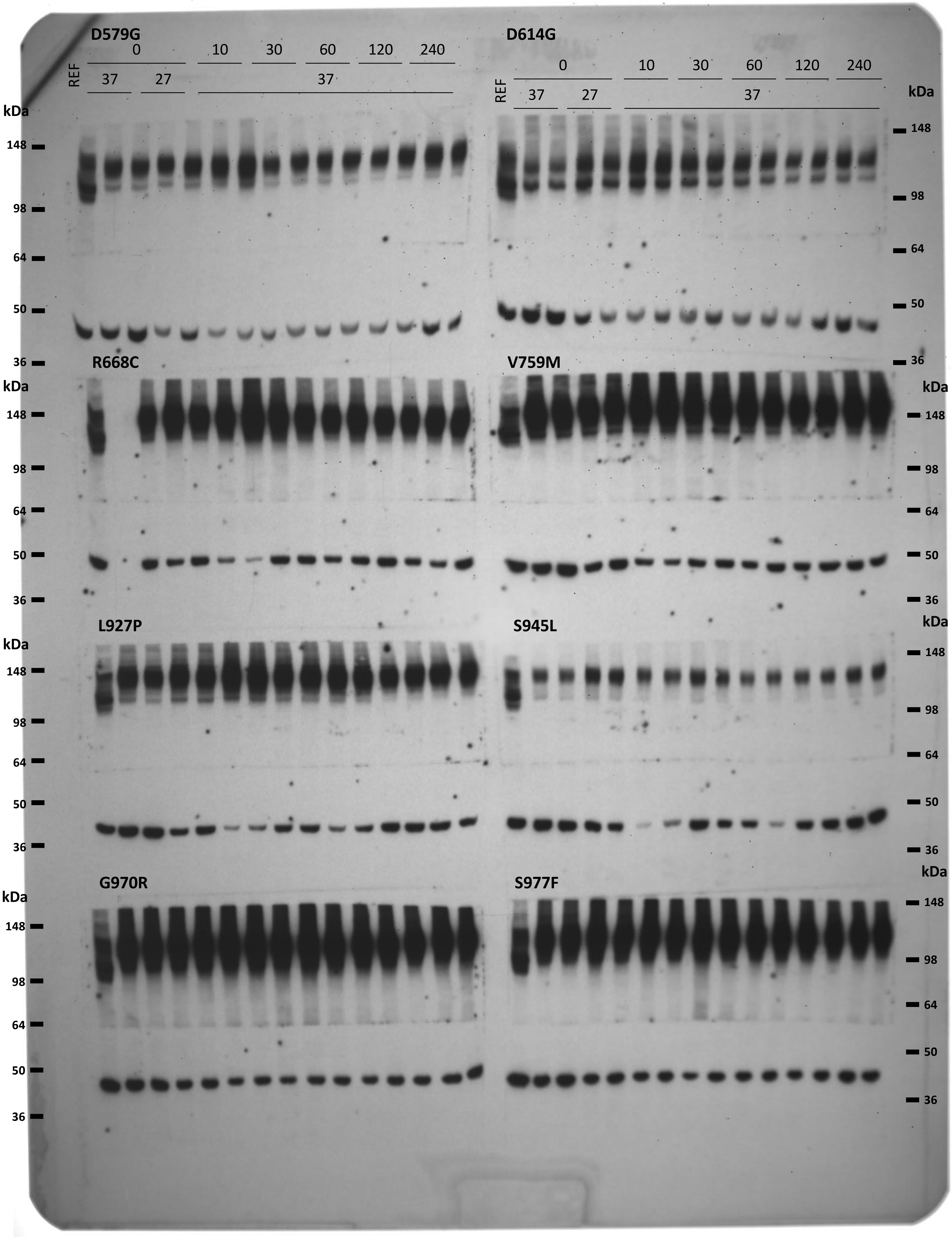

Supplementary Figure 38

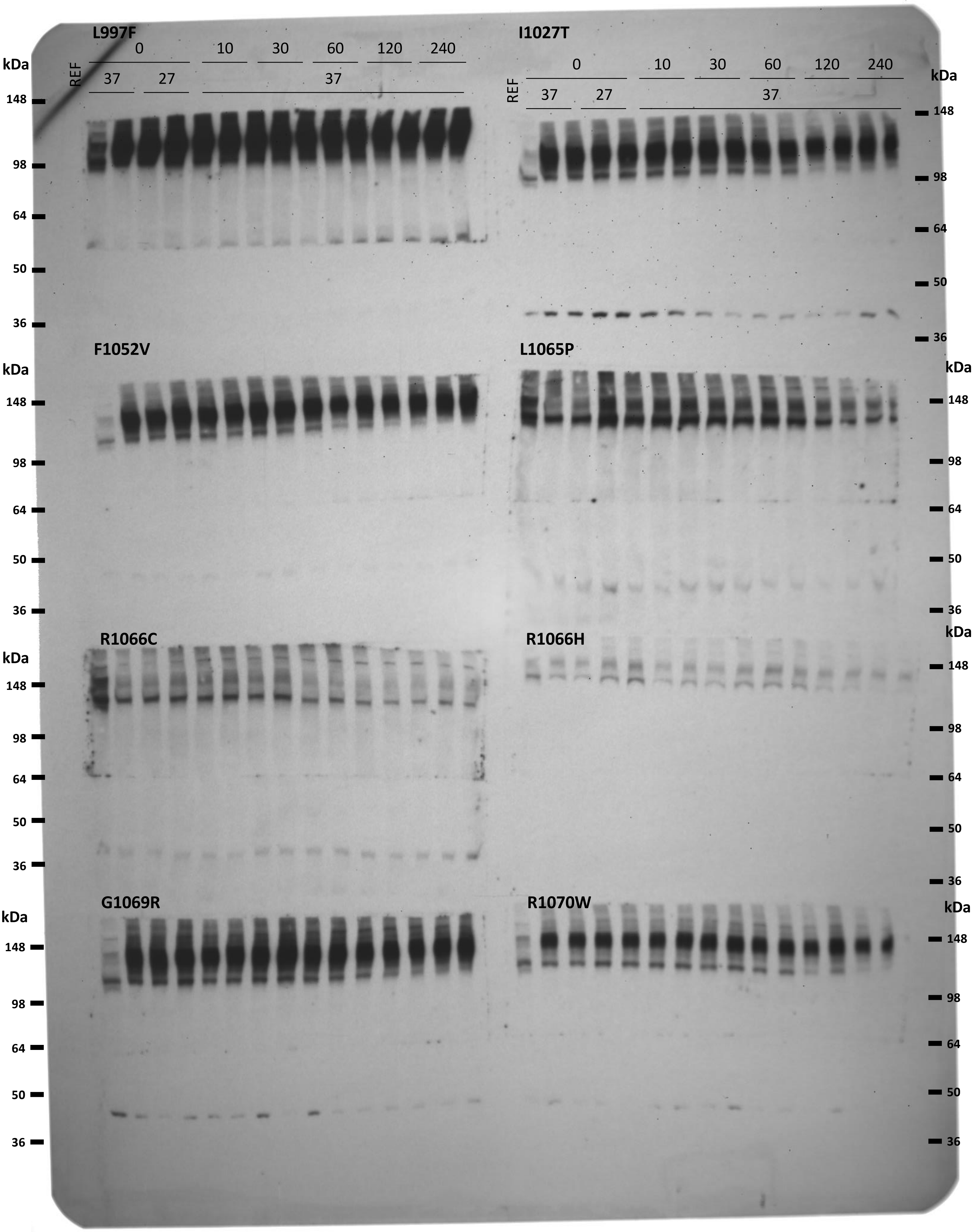

Supplementary Figure 39

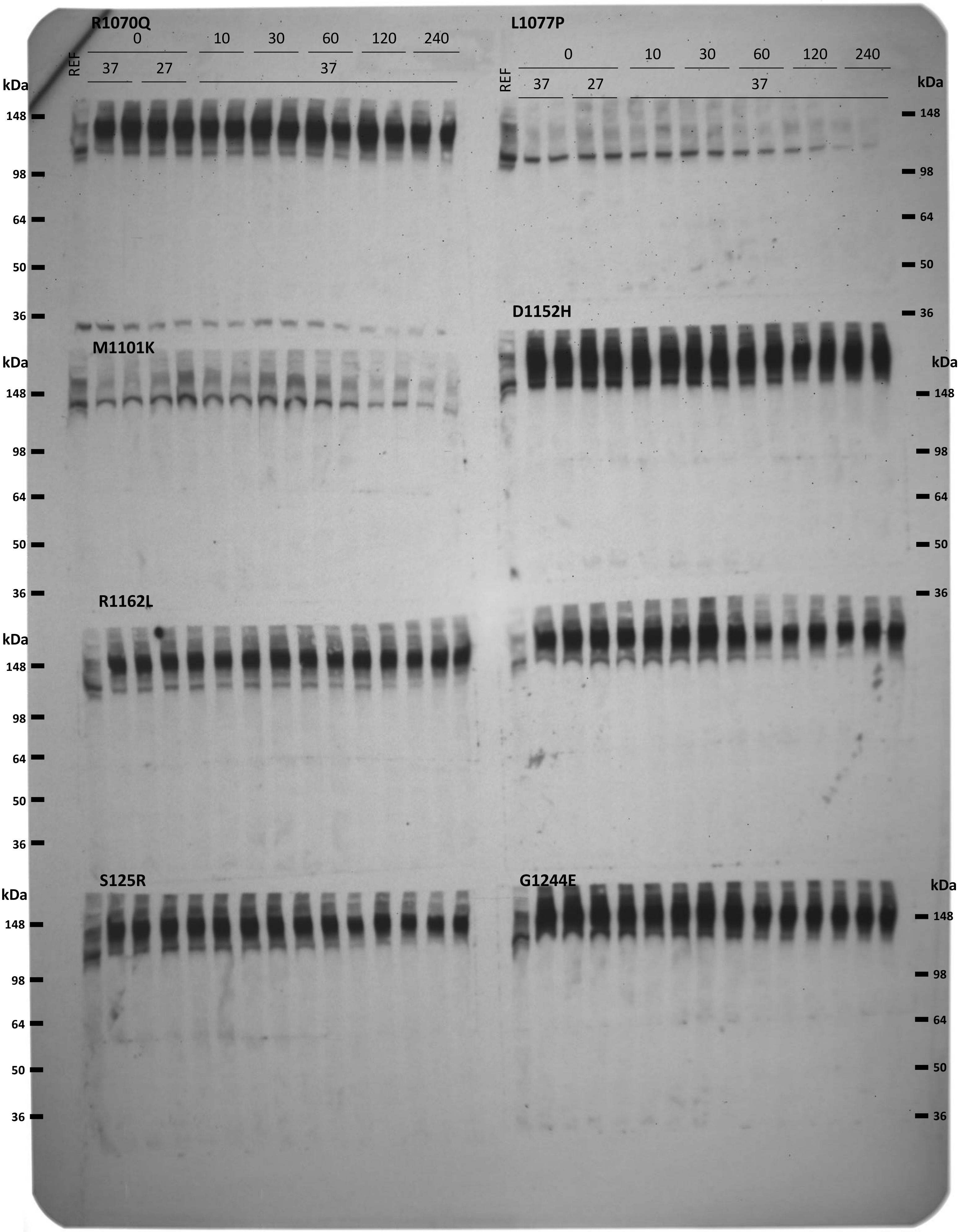

Supplementary Figure 40

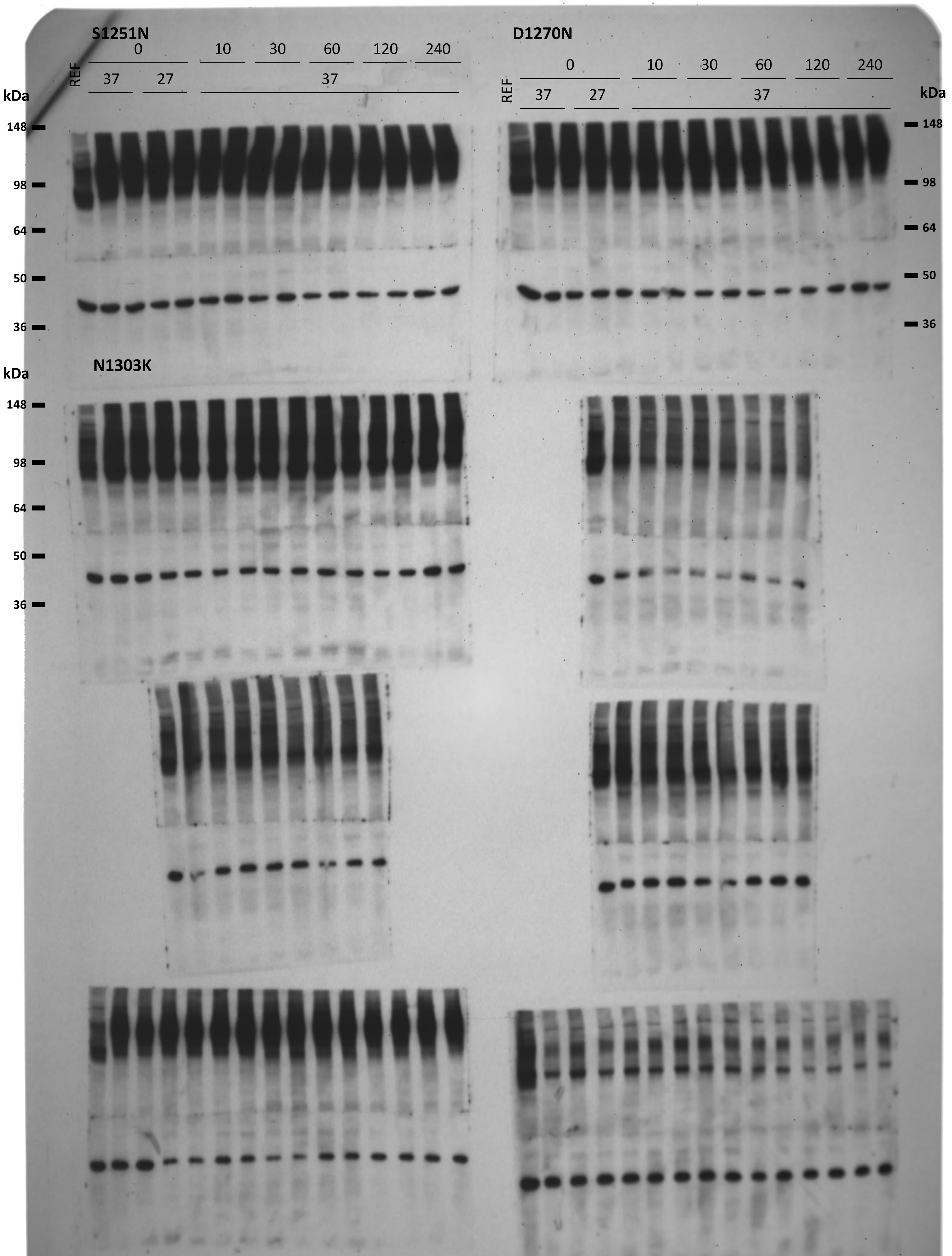

Supplementary Figure 41

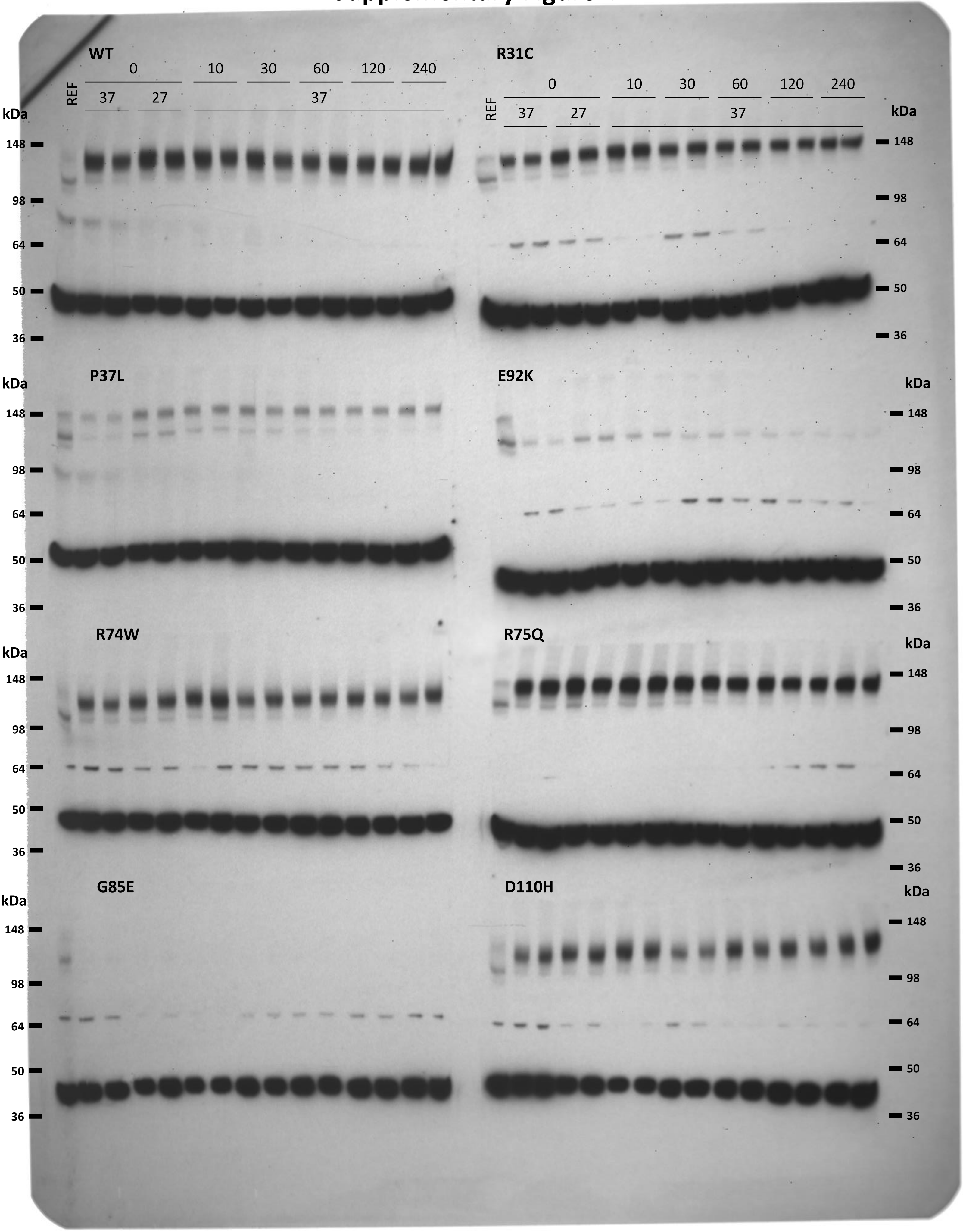

Supplementary Figure 42

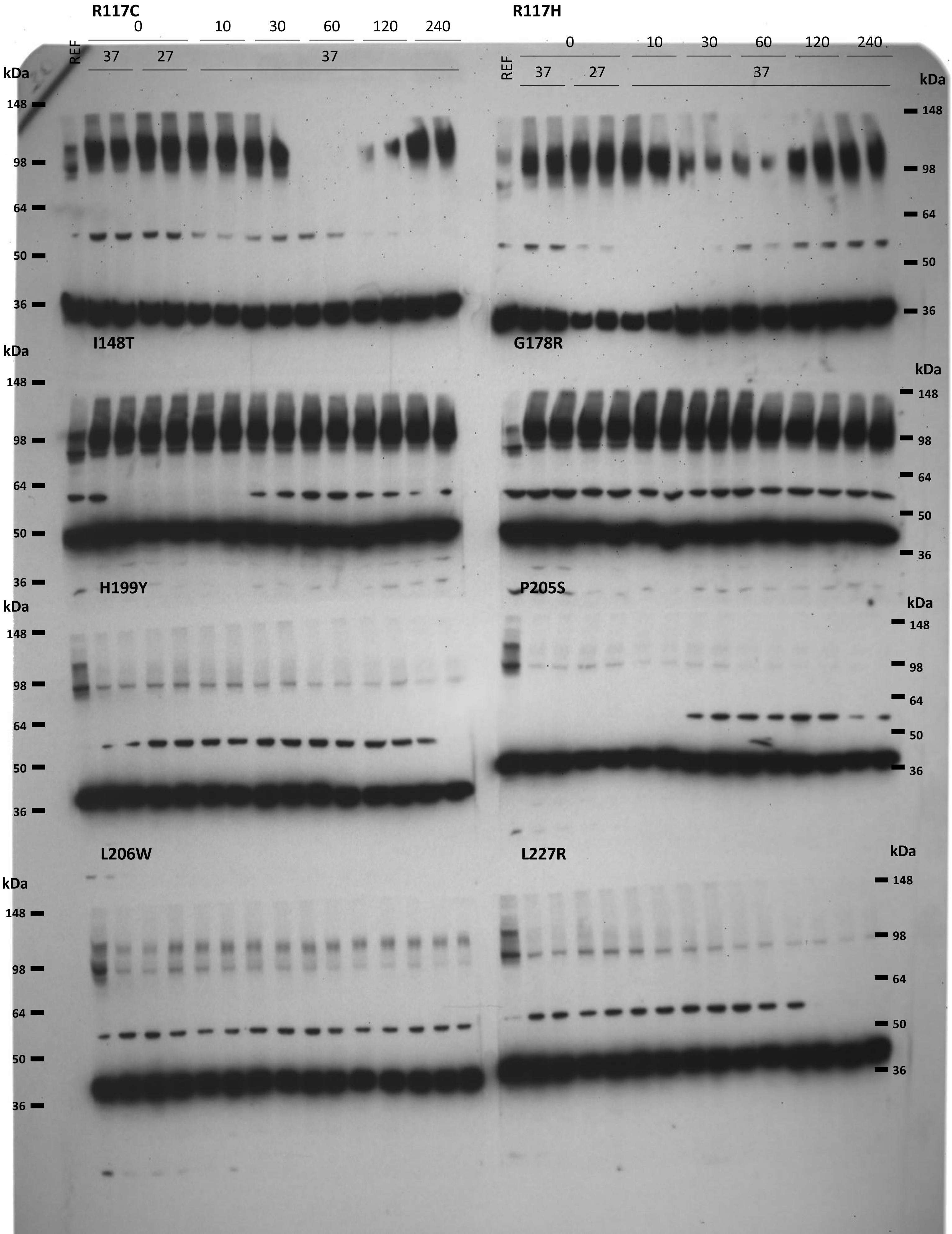

Supplementary Figure 43

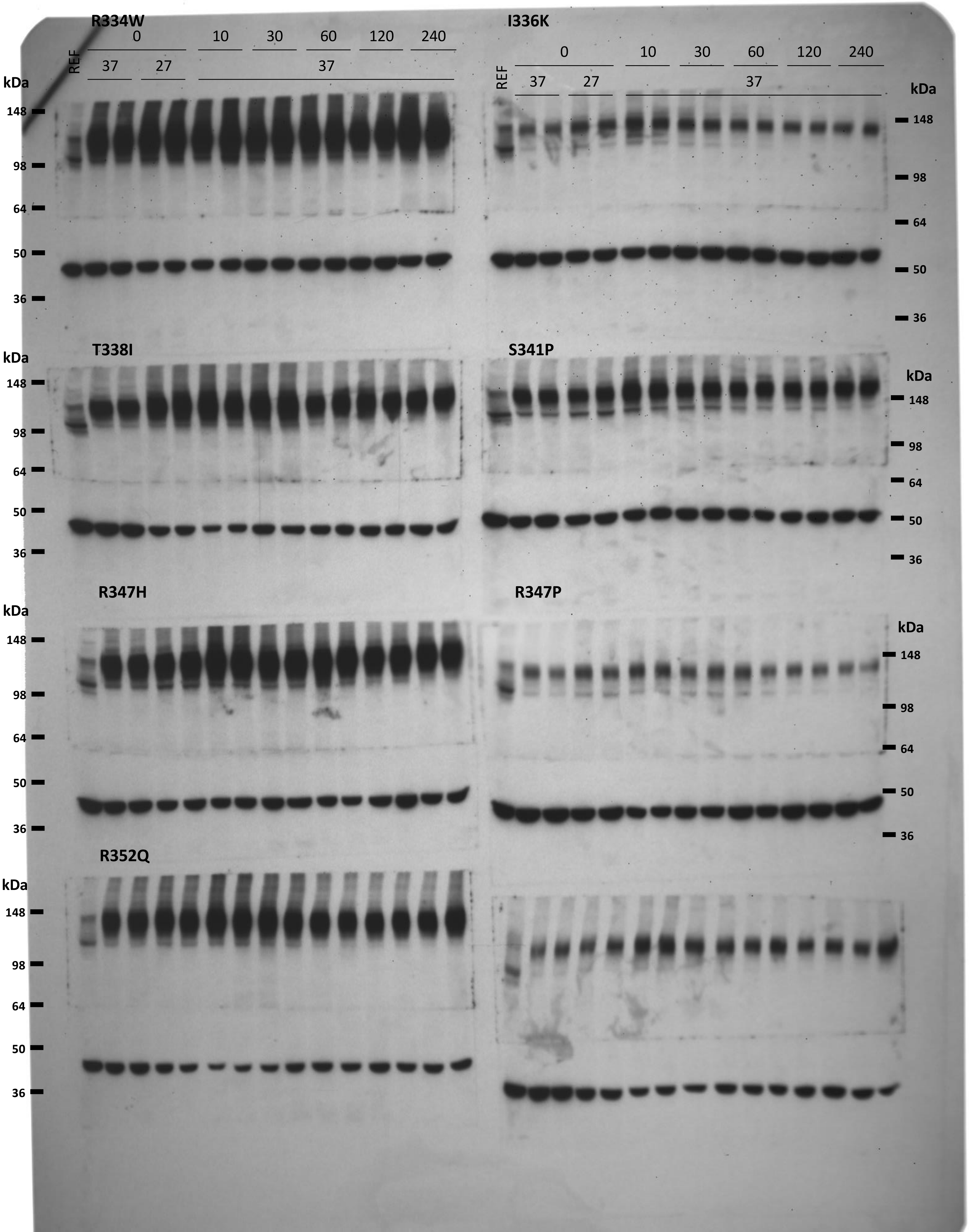

Supplementary Figure 44

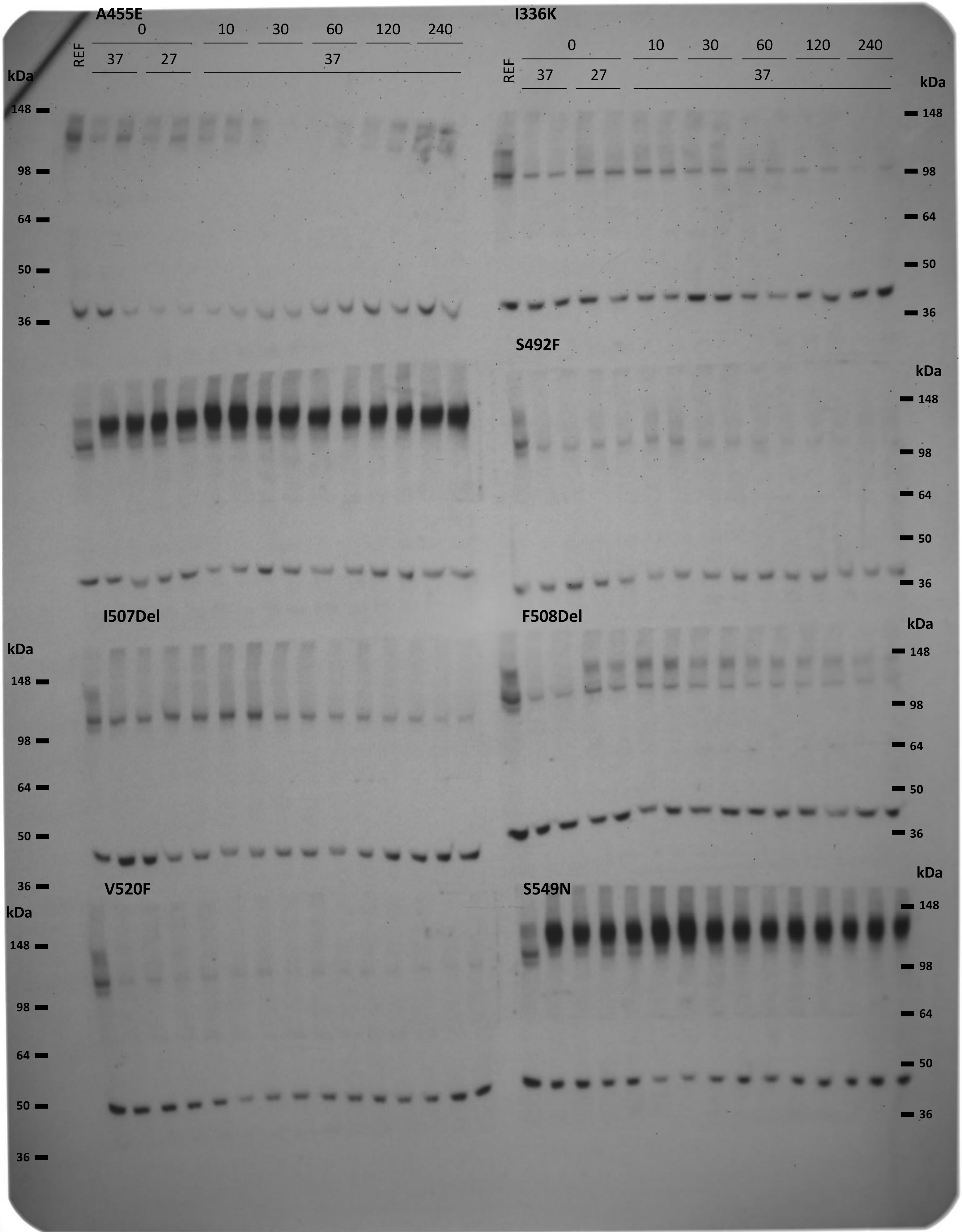

Supplementary Figure 45

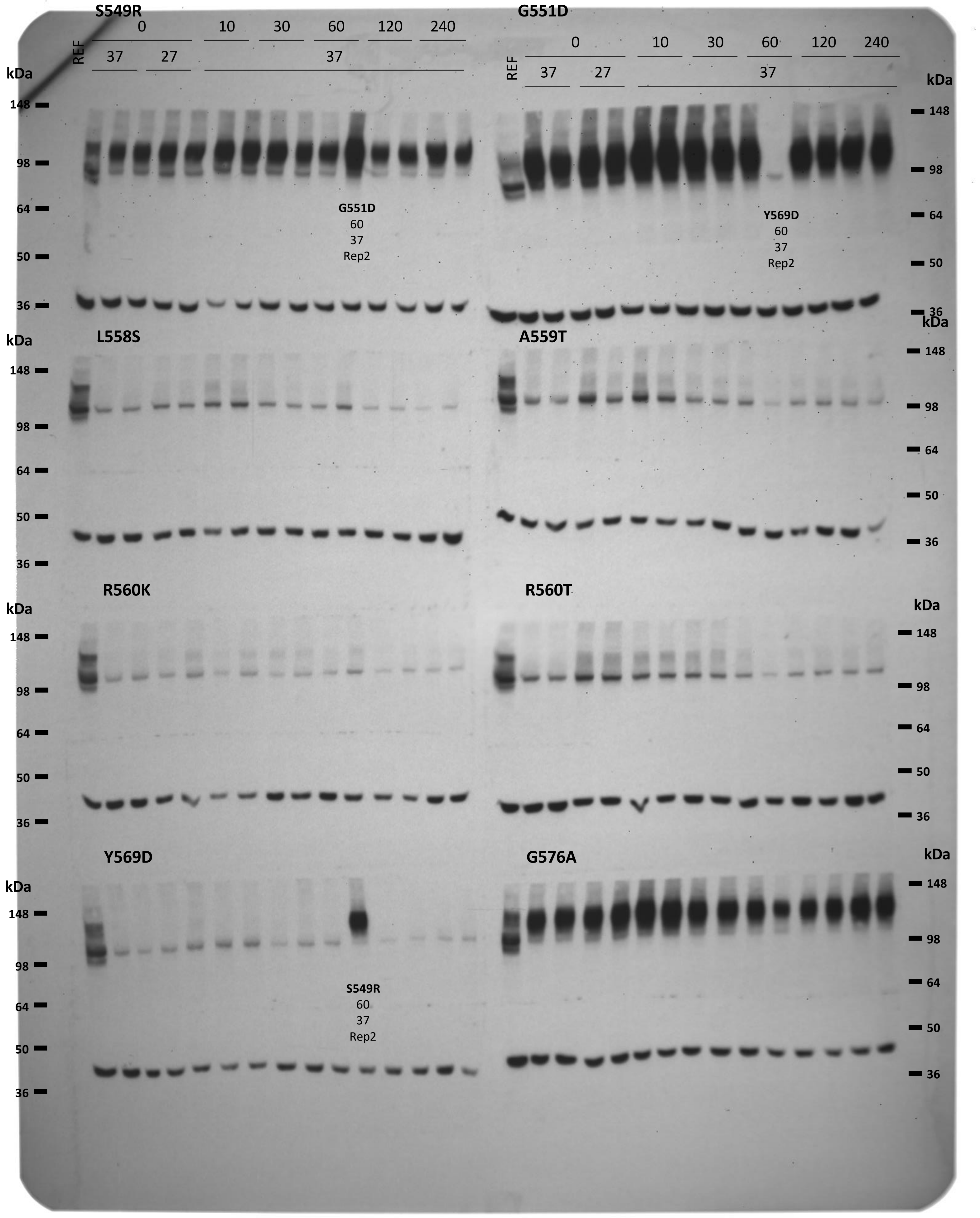

Supplementary Figure 46

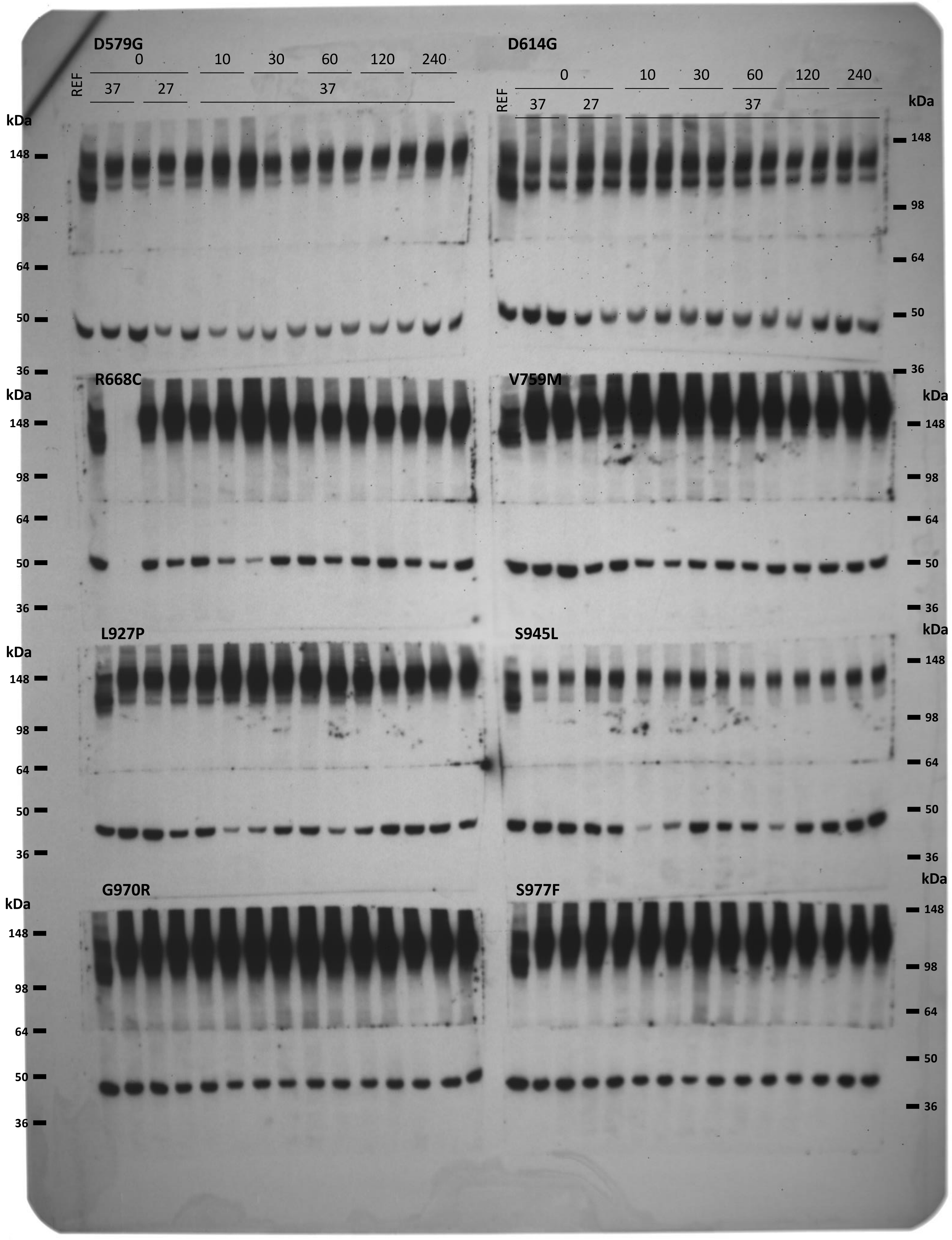

Supplementary Figure 47

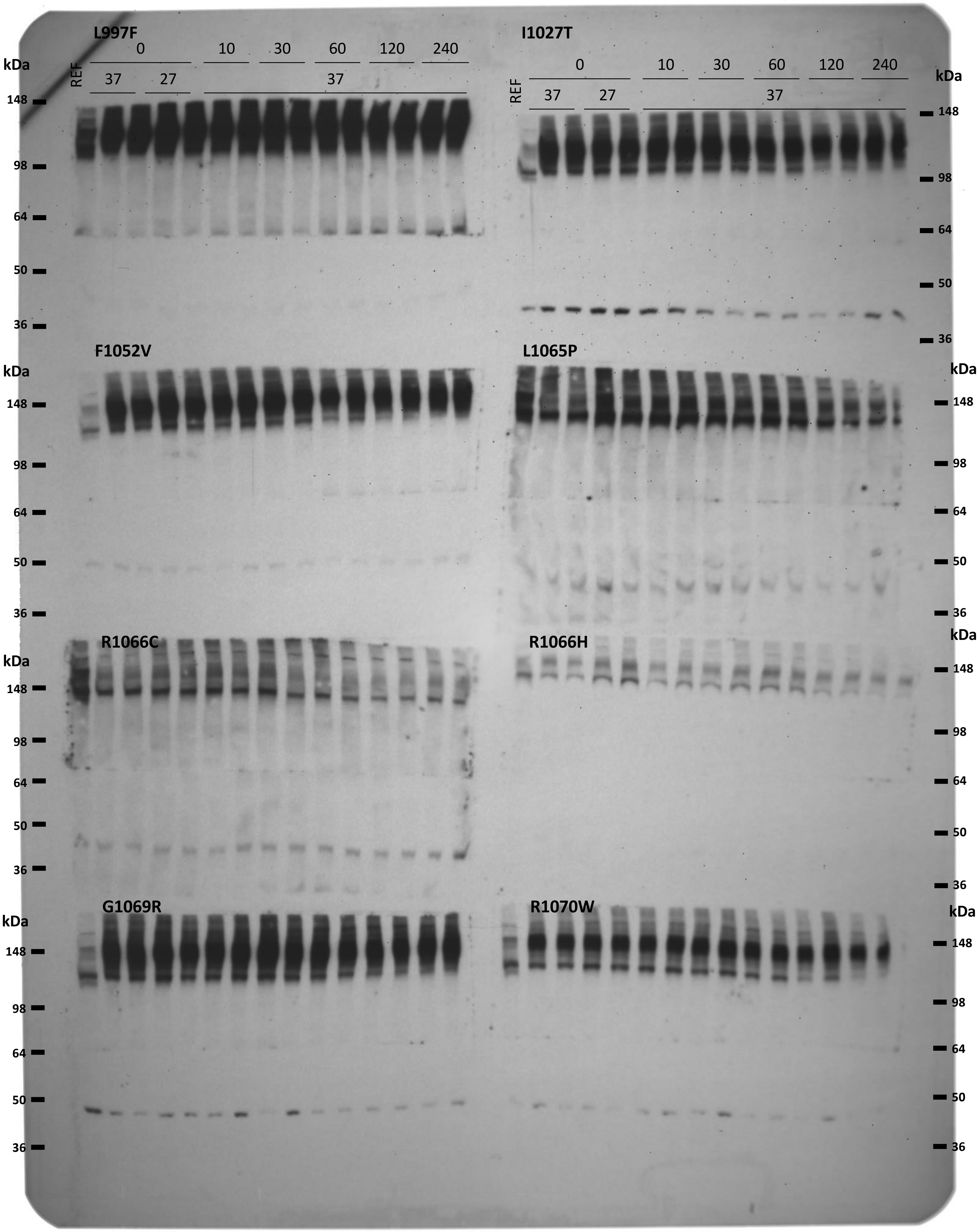

Supplementary Figure 48

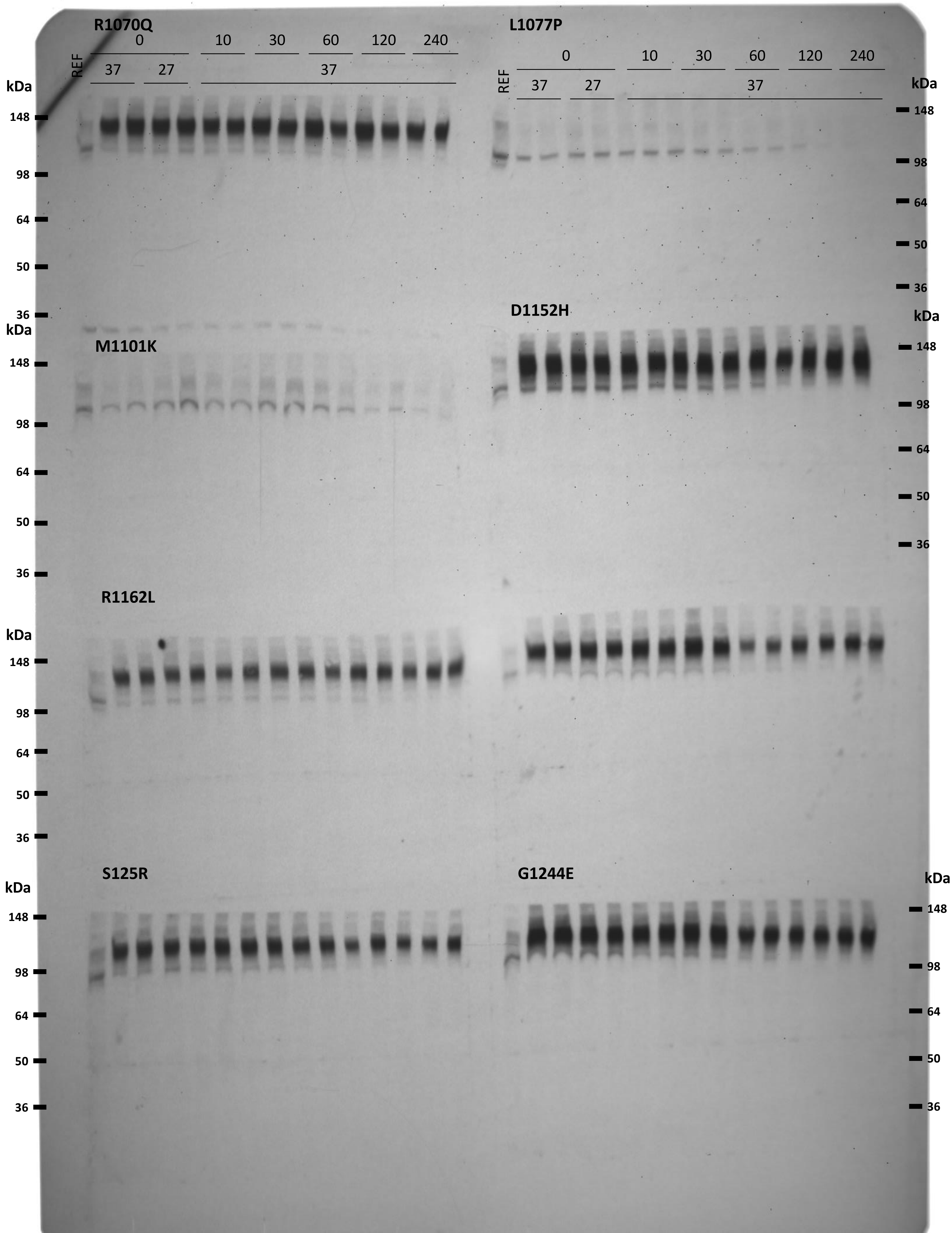

Supplementary Figure 49

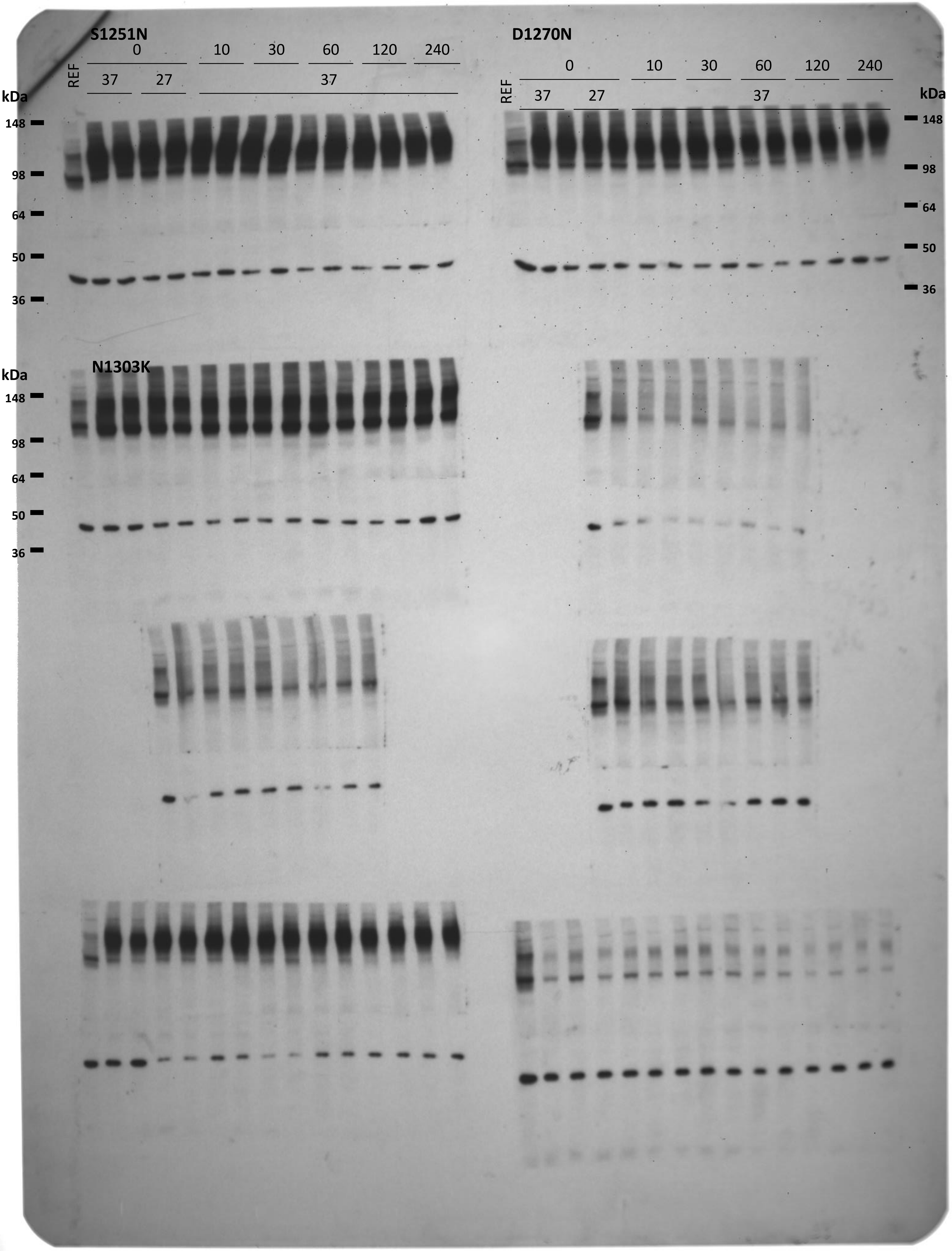

Supplementary Figure 50

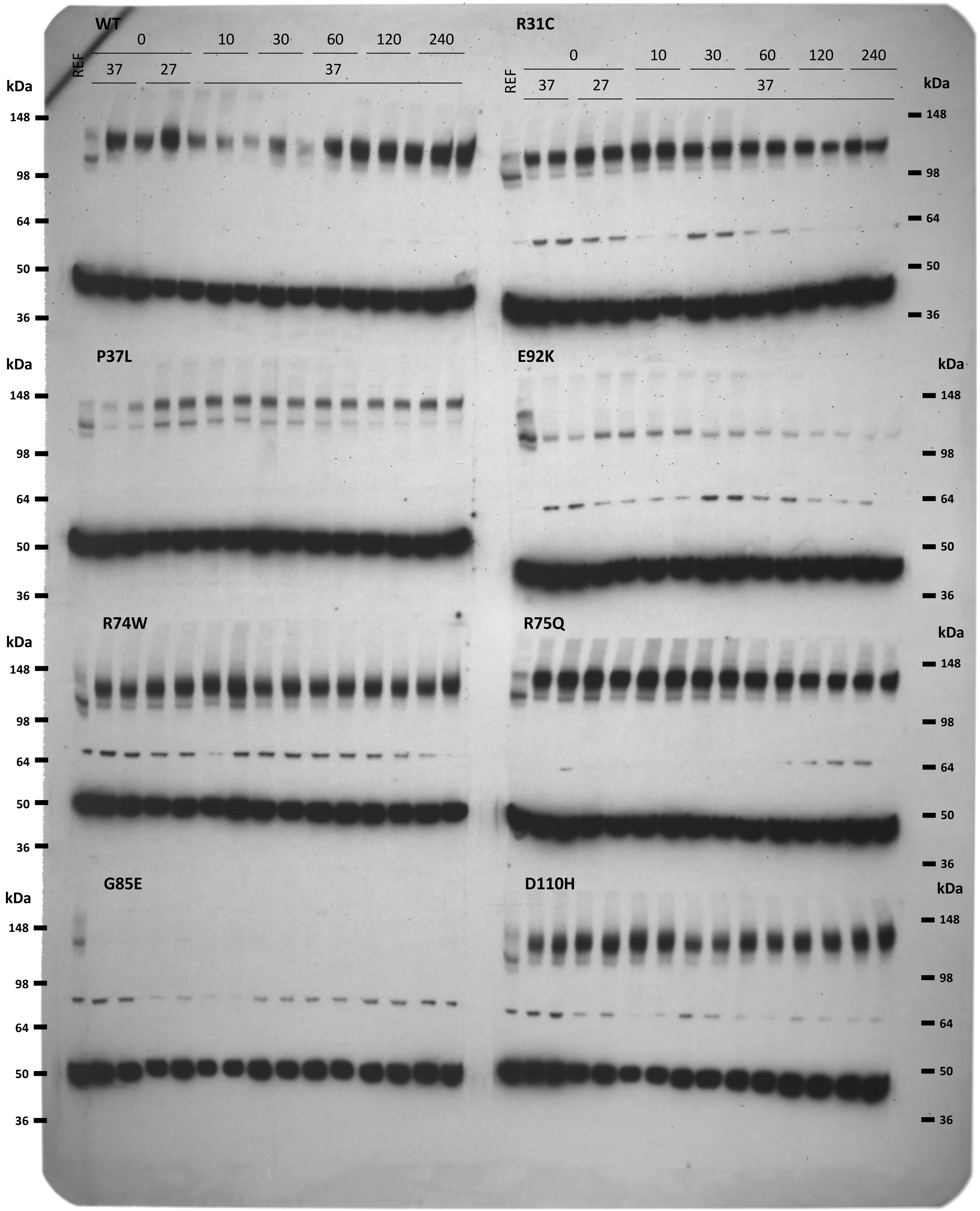

Supplementary Figure 51

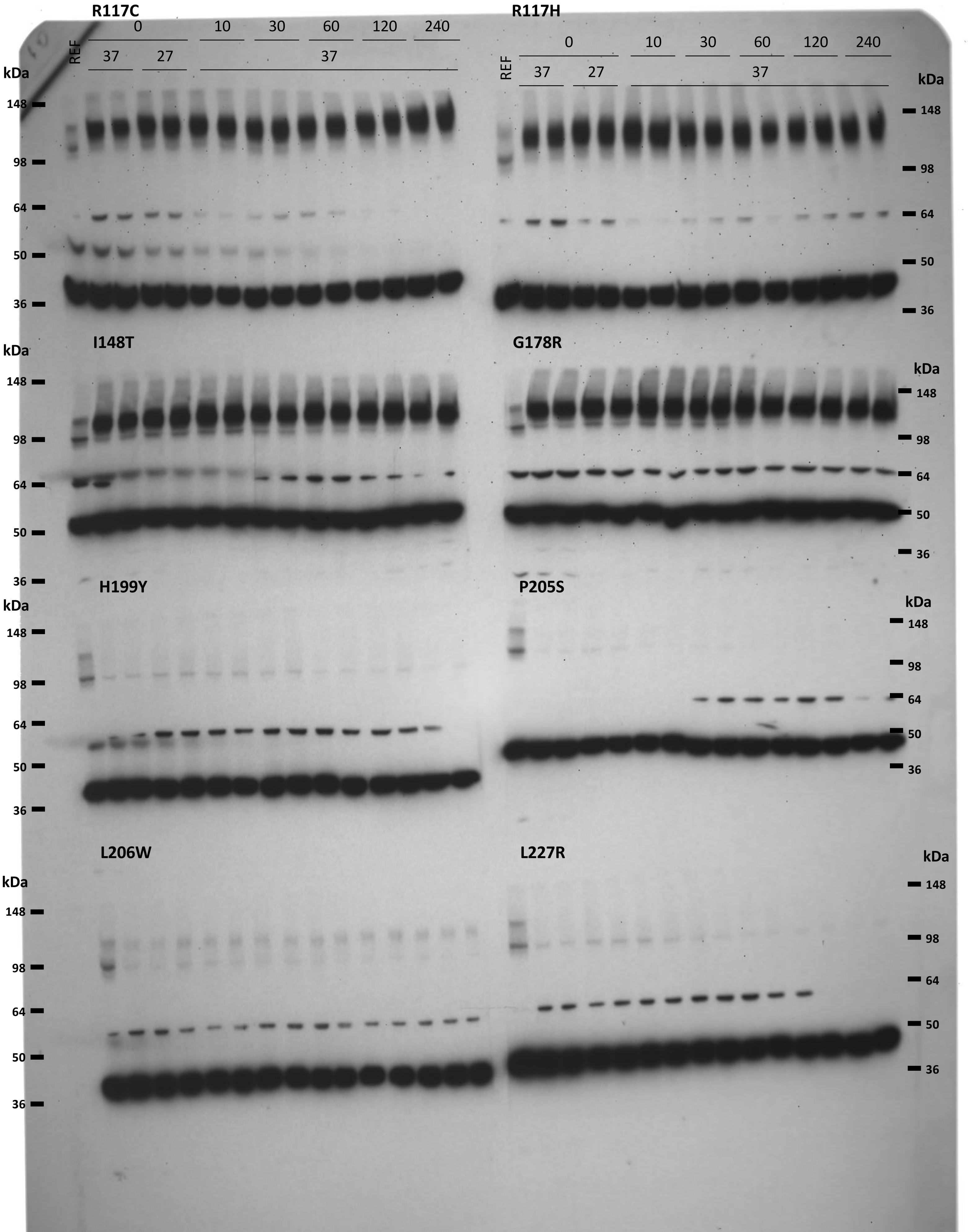

Supplementary Figure 52

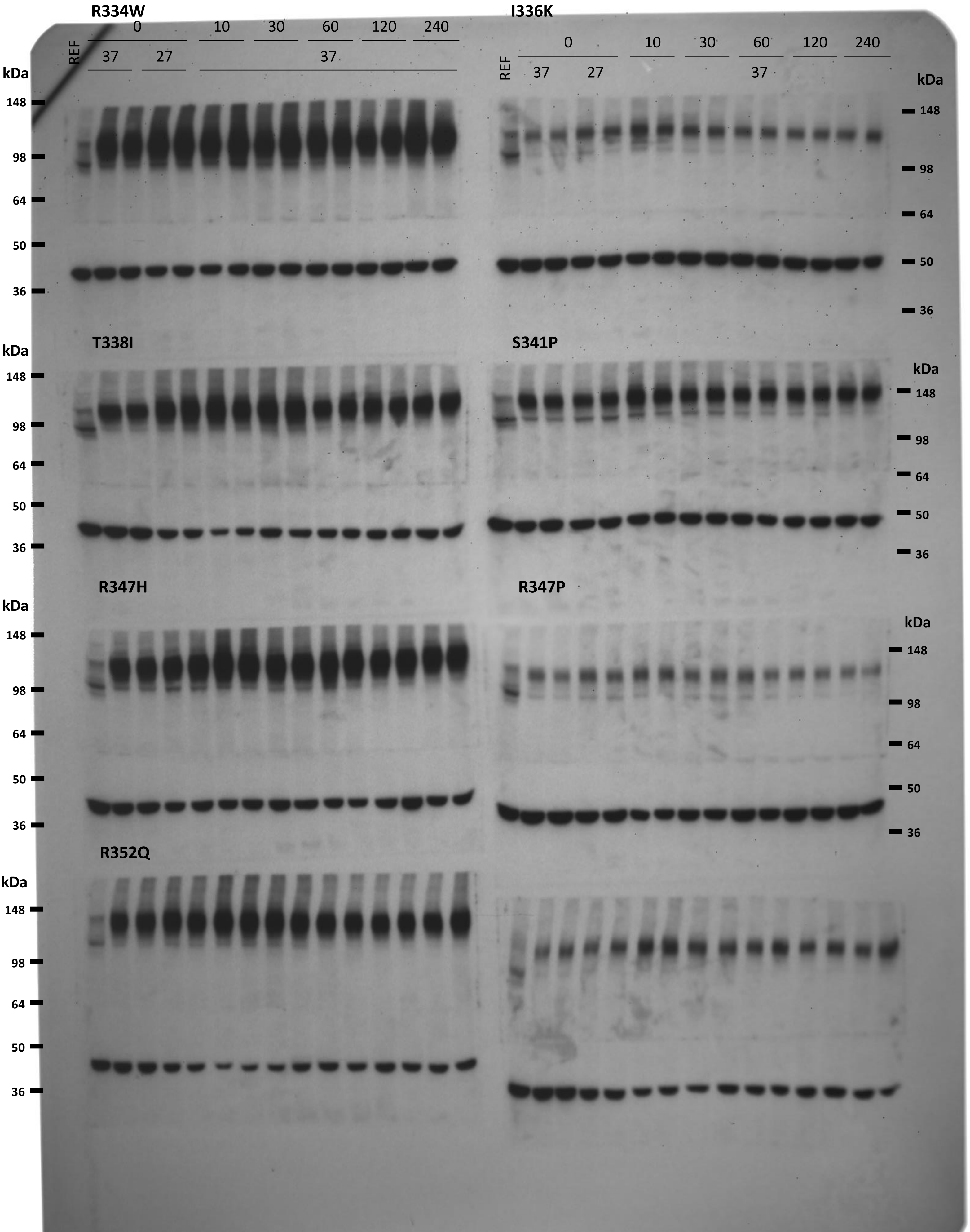

Supplementary Figure 53

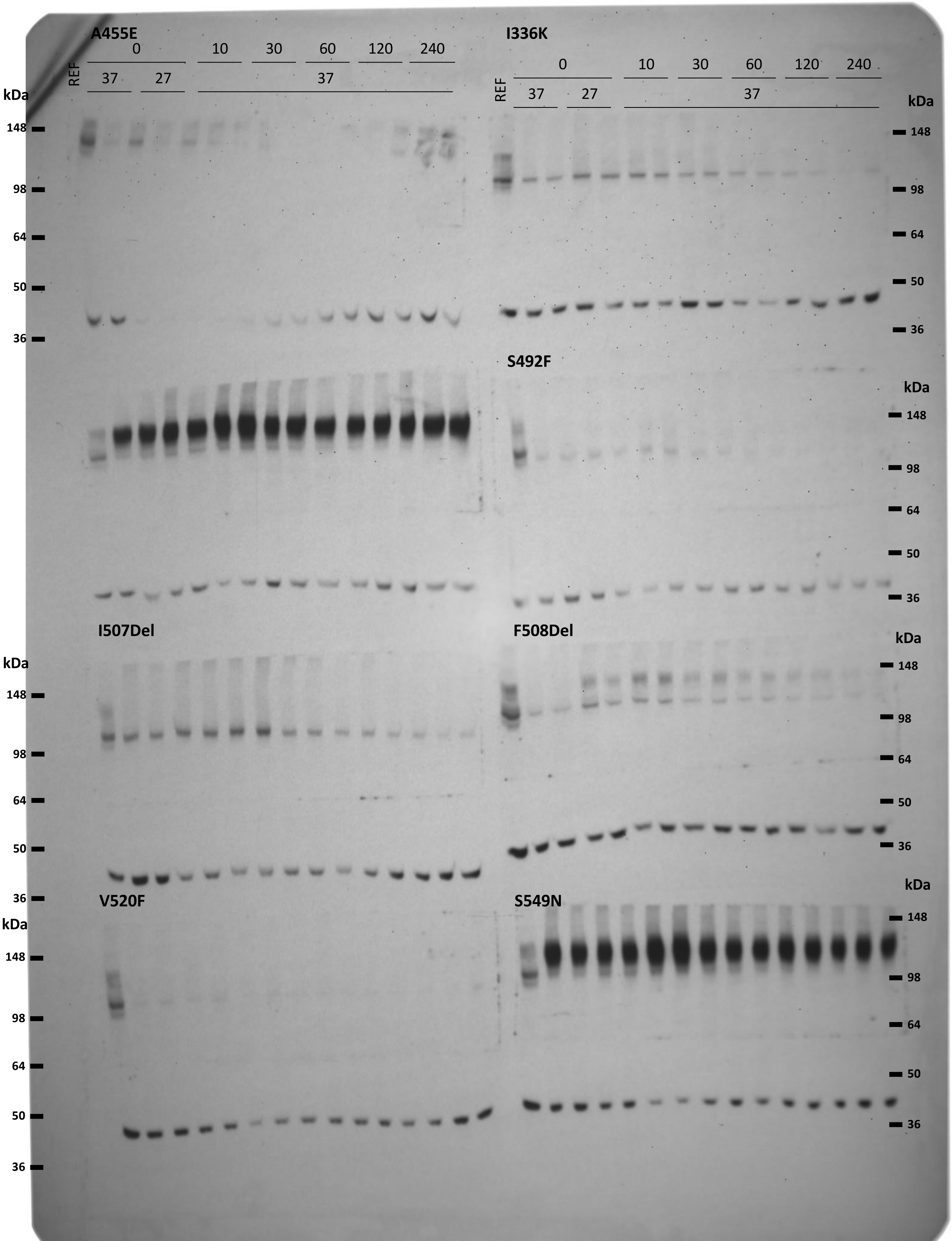

Supplementary Figure 54

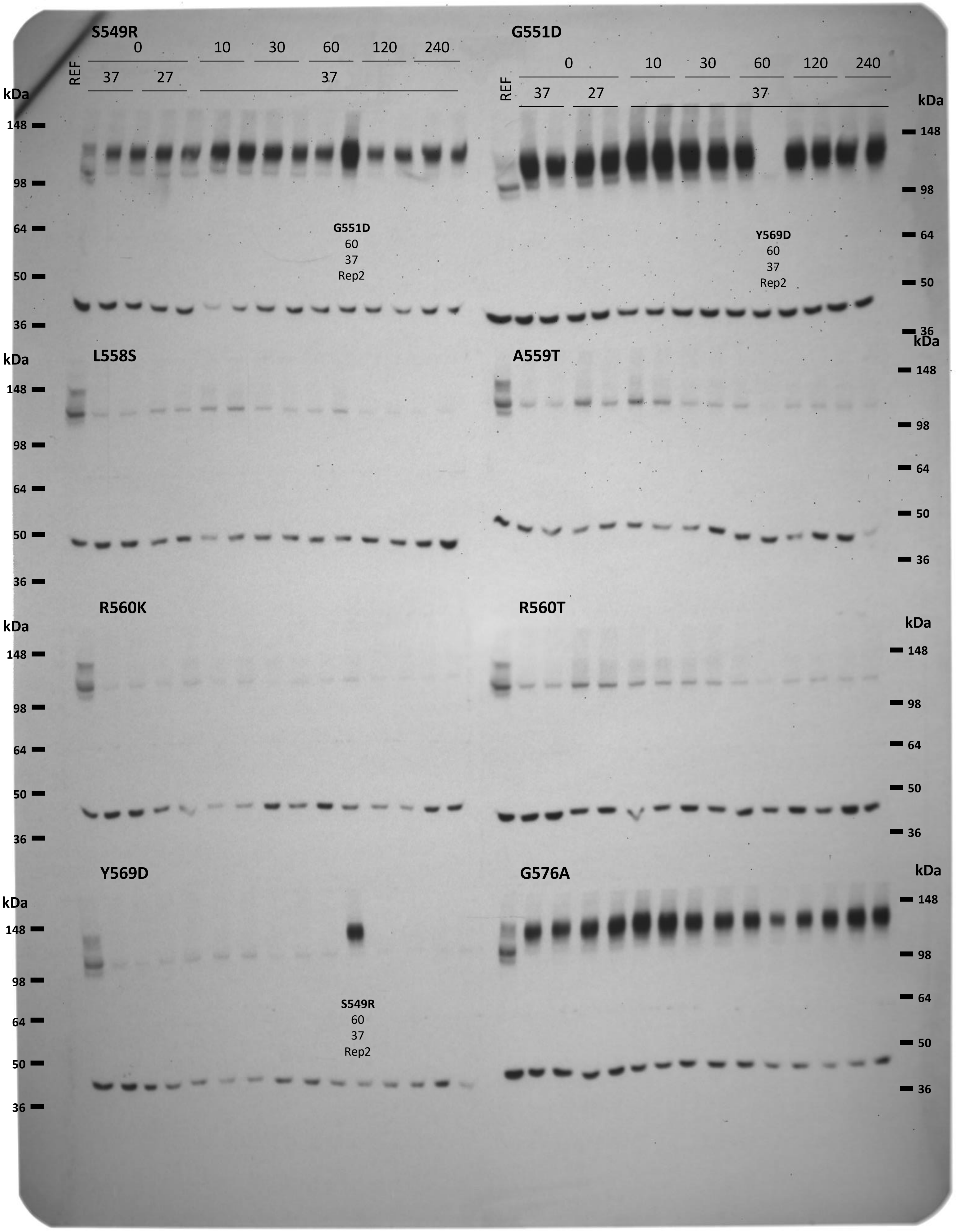

Supplementary Figure 55

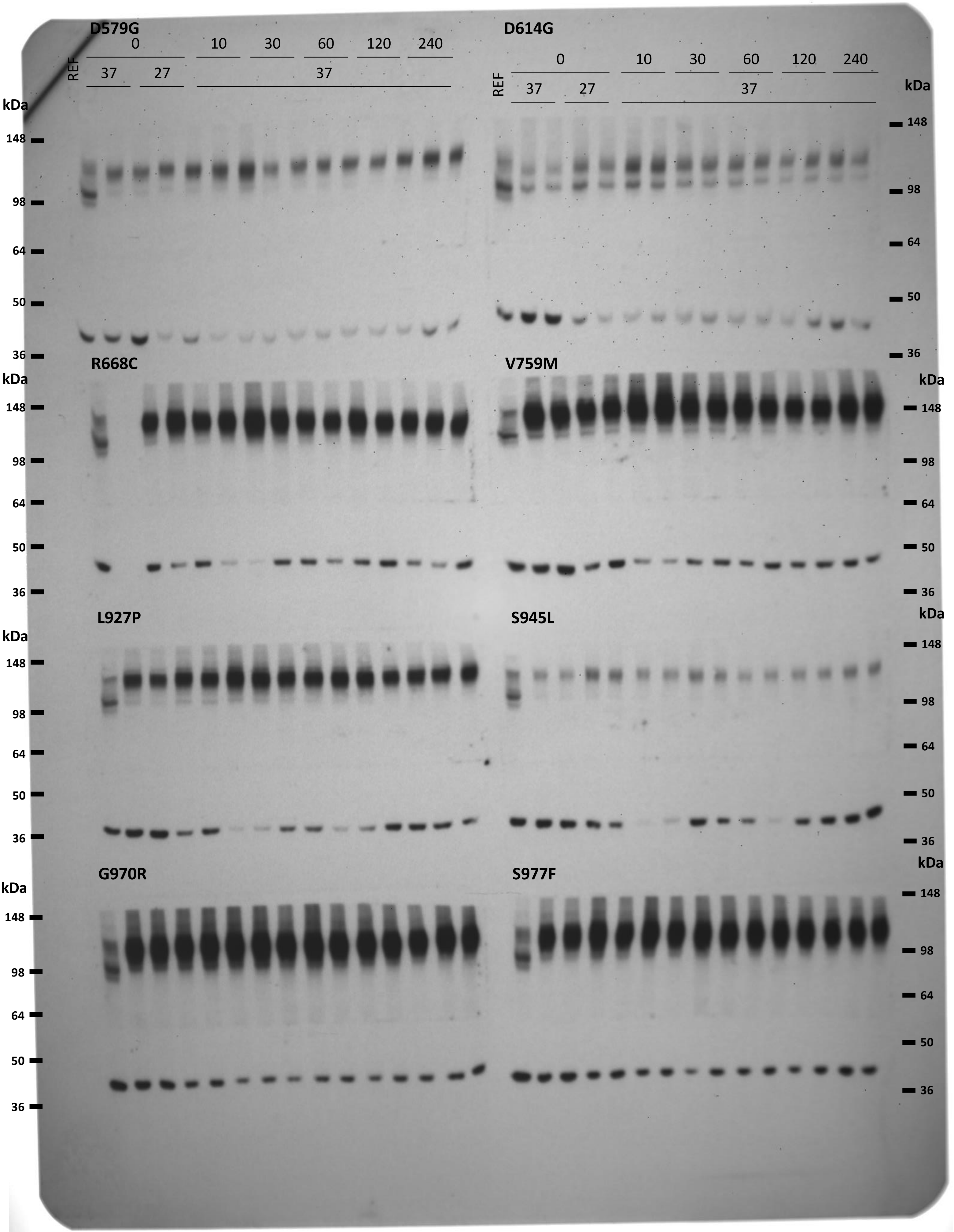

Supplementary Figure 56

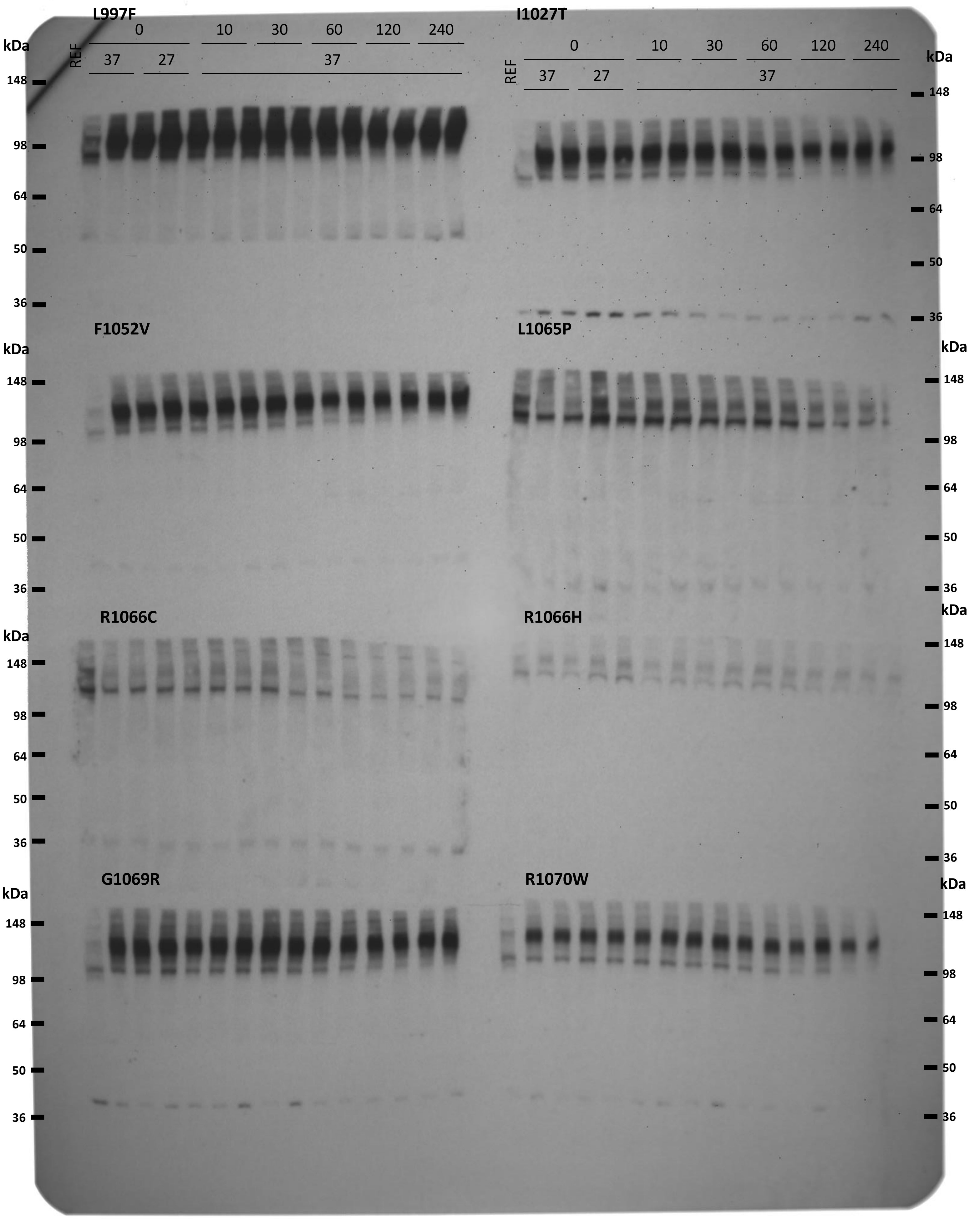

Supplementary Figure 57

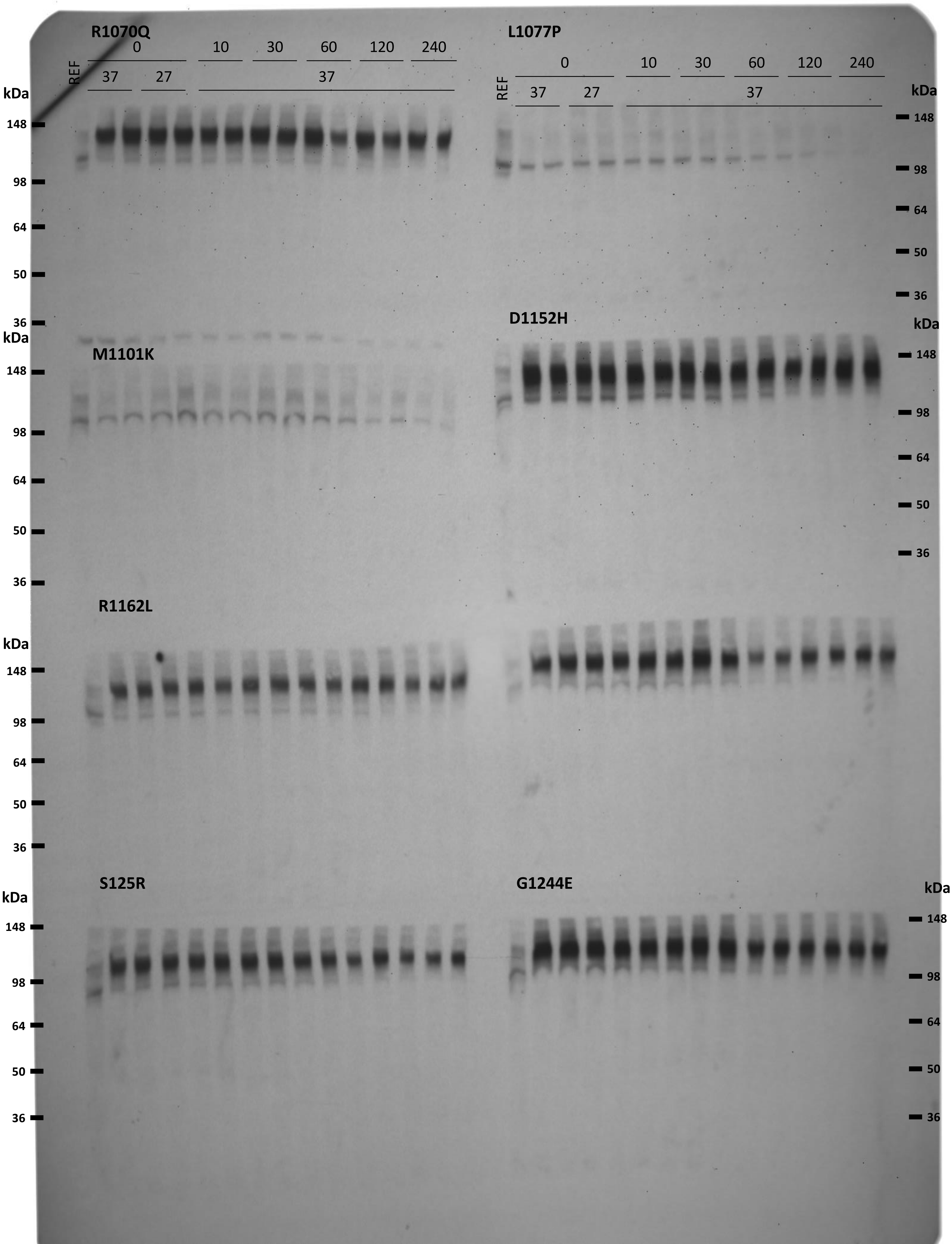

Supplementary Figure 58

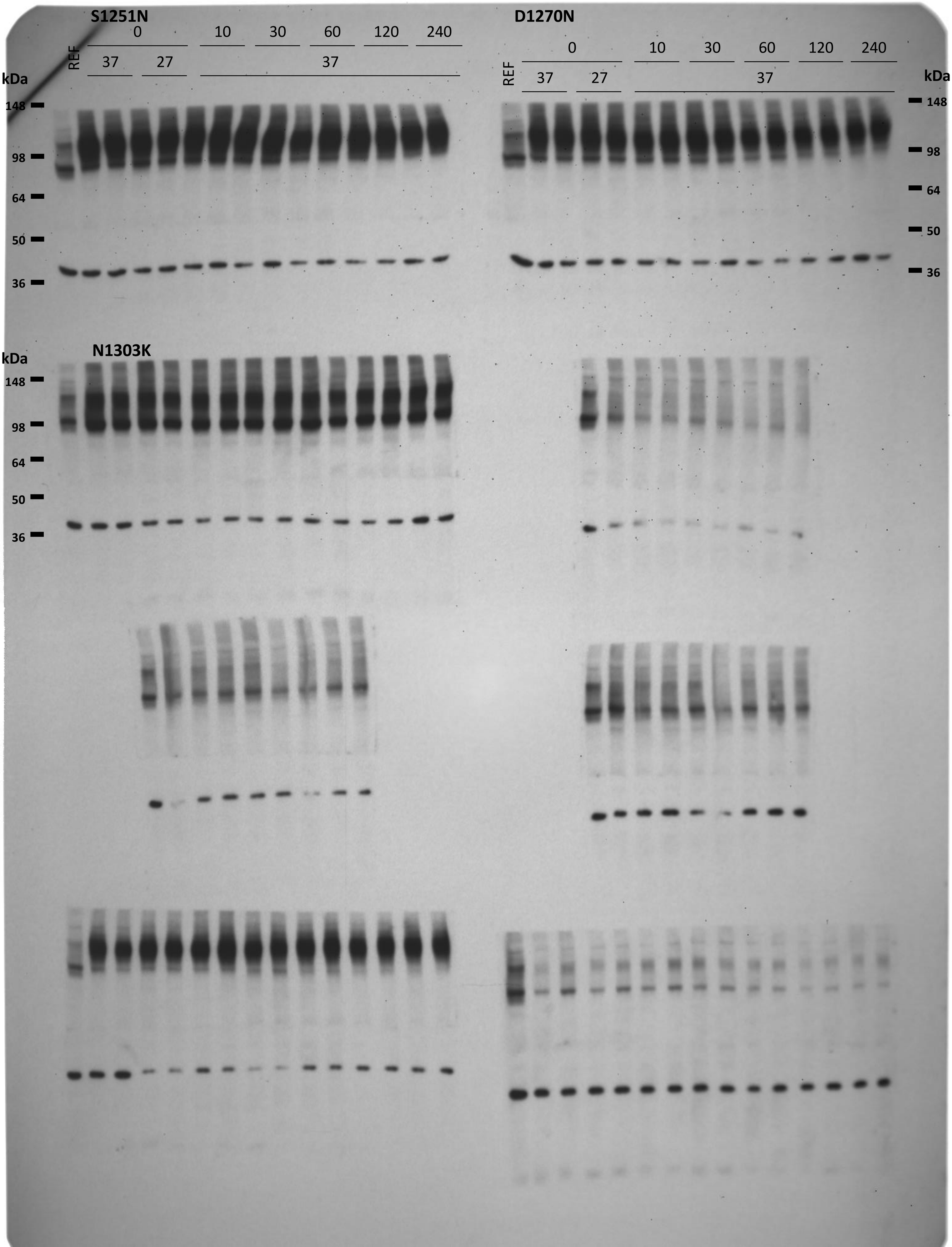

Supplementary Figure 59

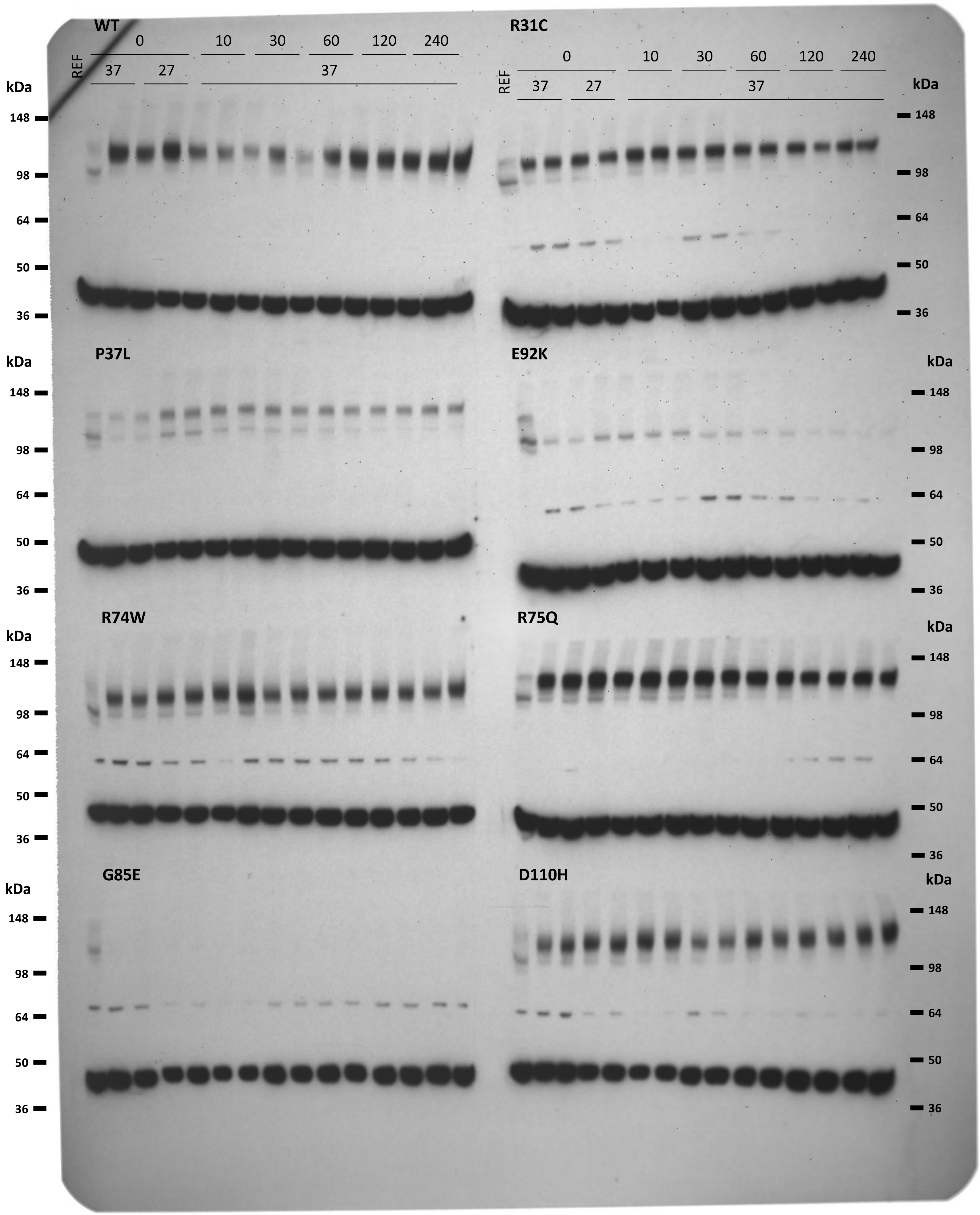

Supplementary Figure 60

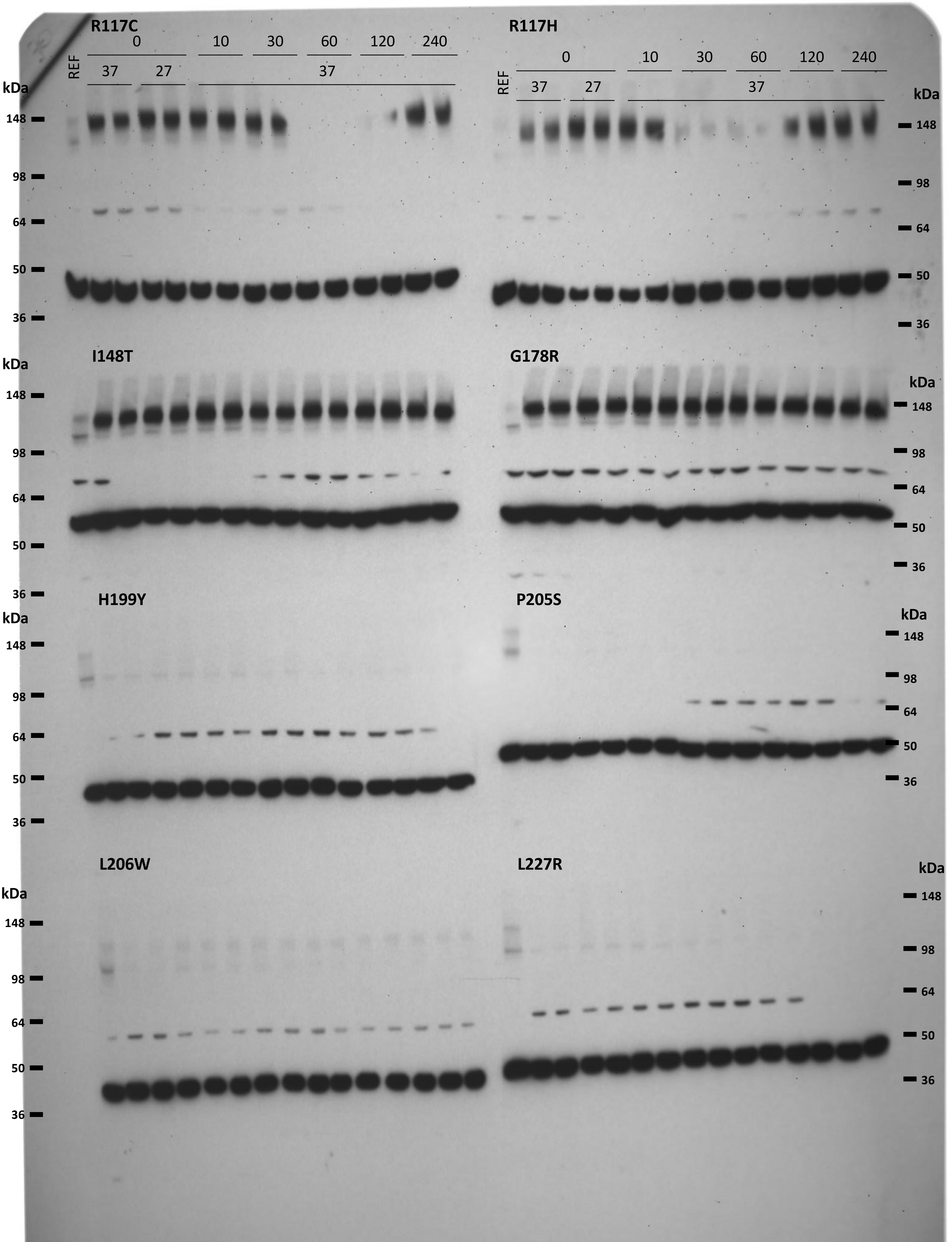

Supplementary Figure 61

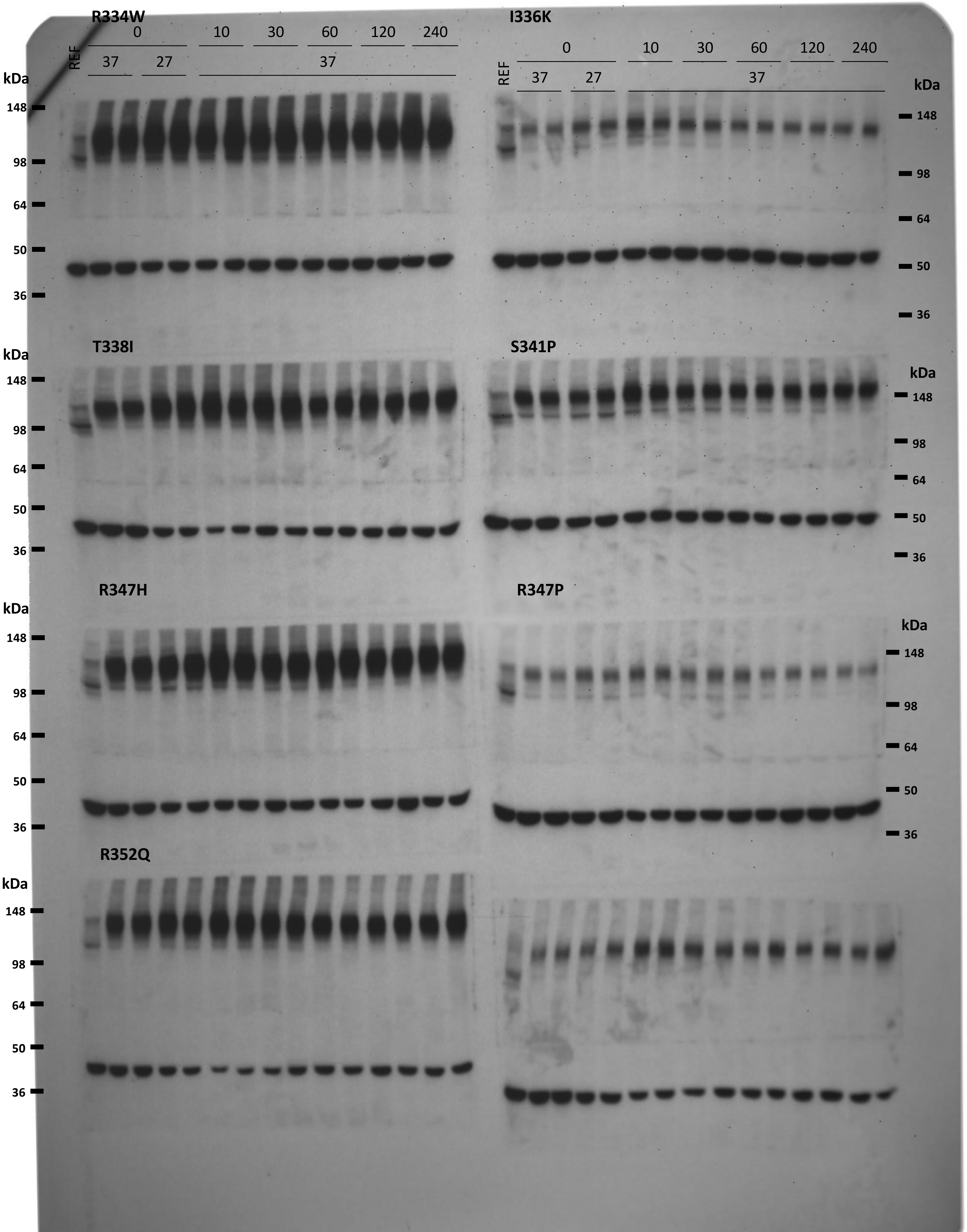

Supplementary Figure 62

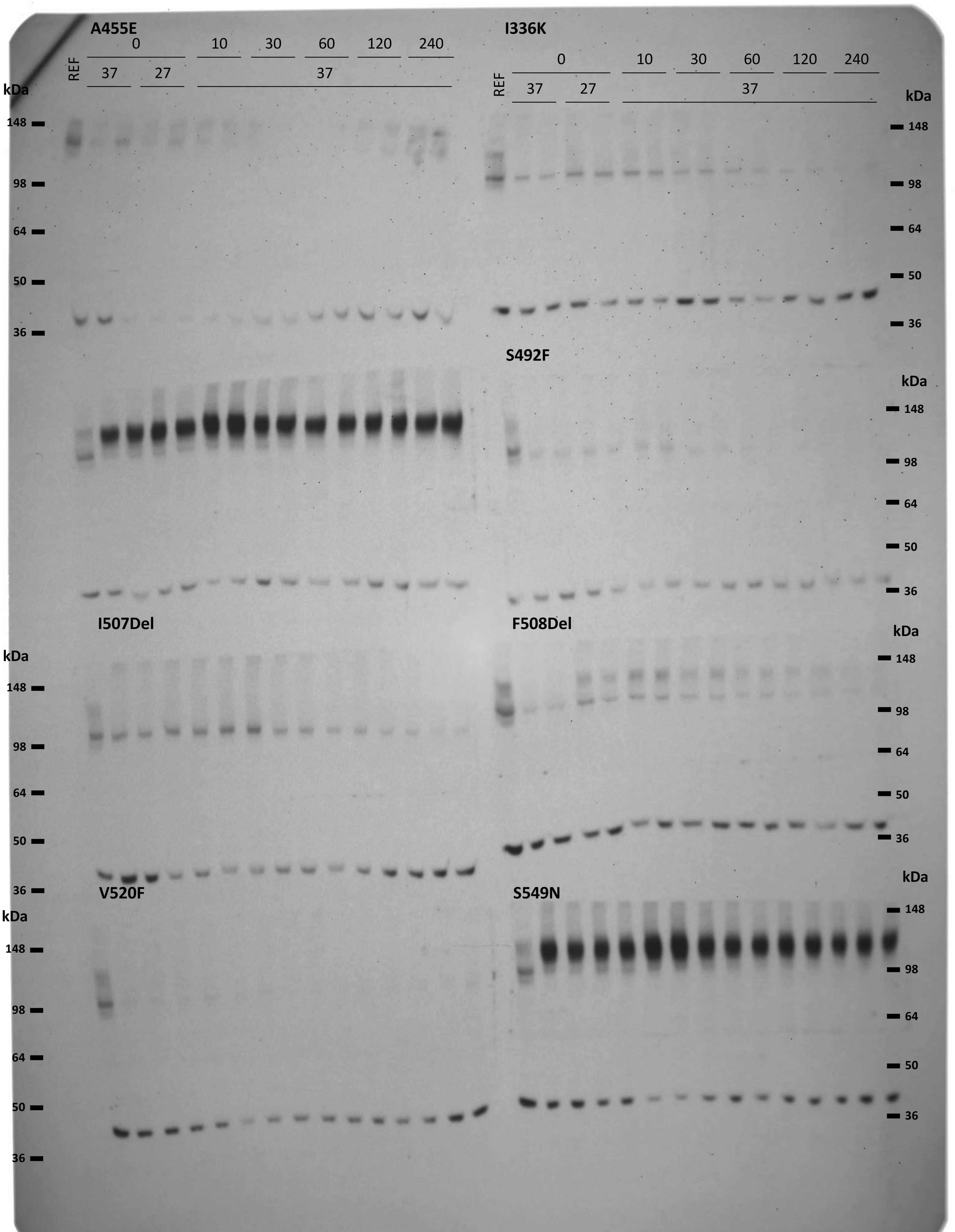

Supplementary Figure 63

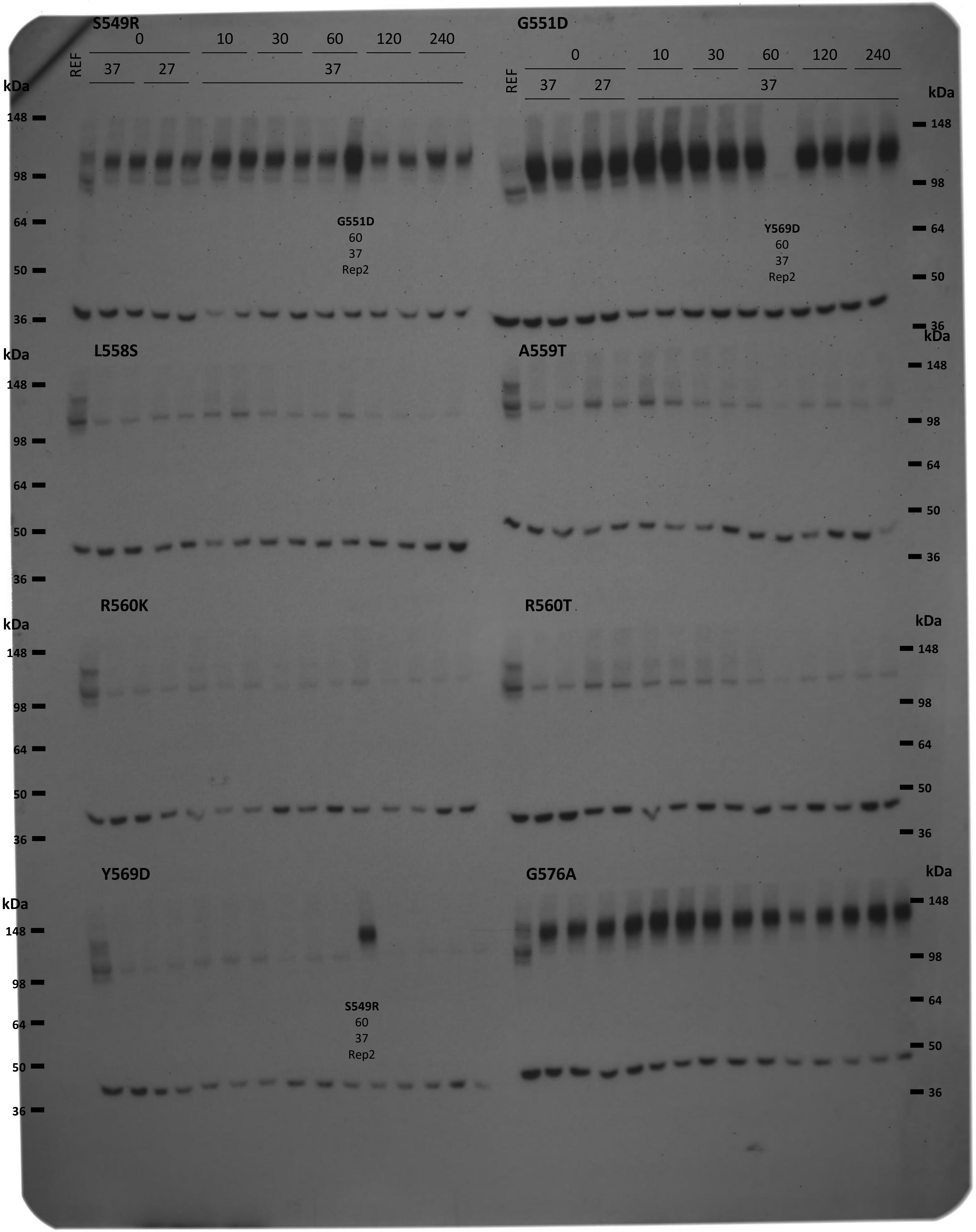

Supplementary Figure 64

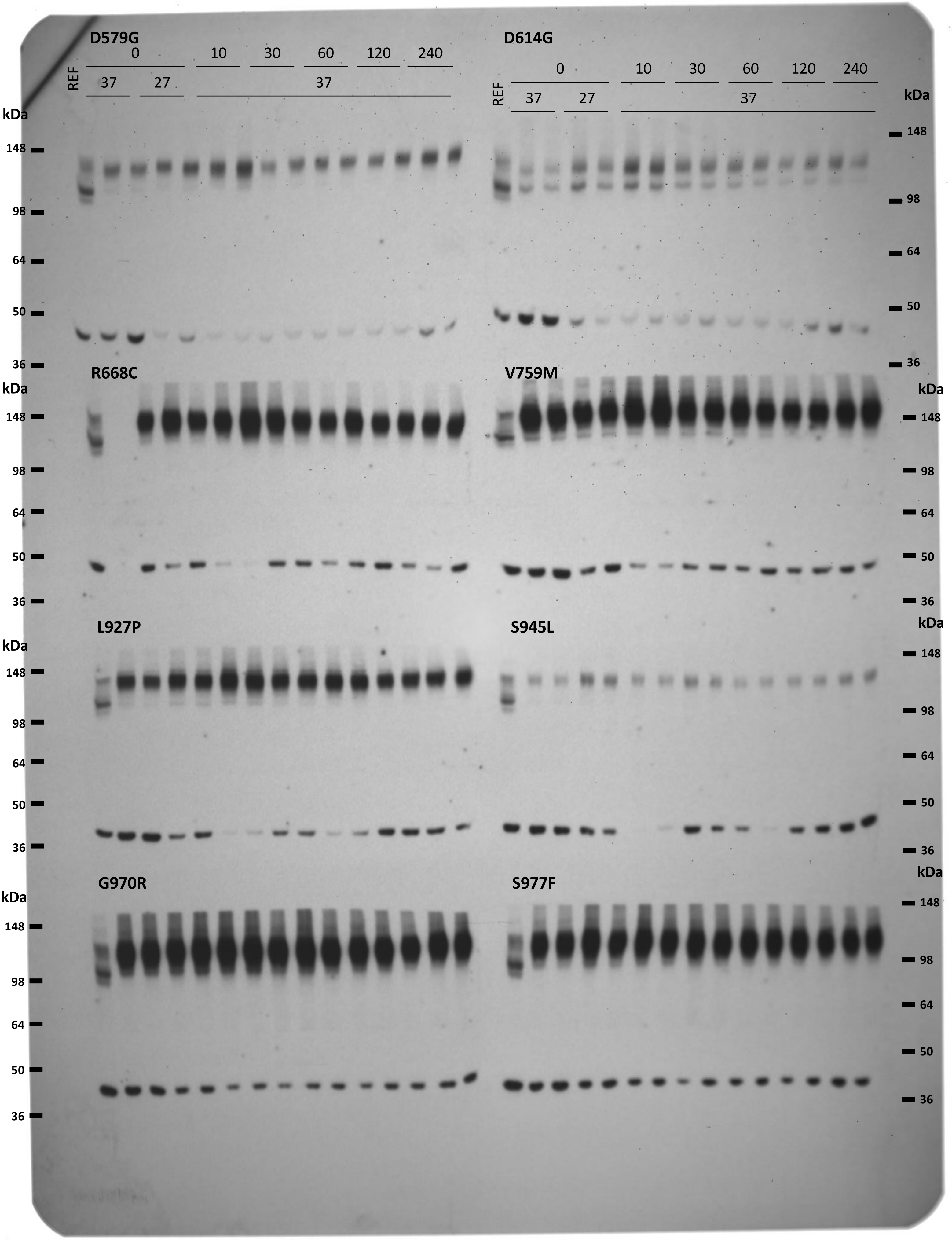

Supplementary Figure 65

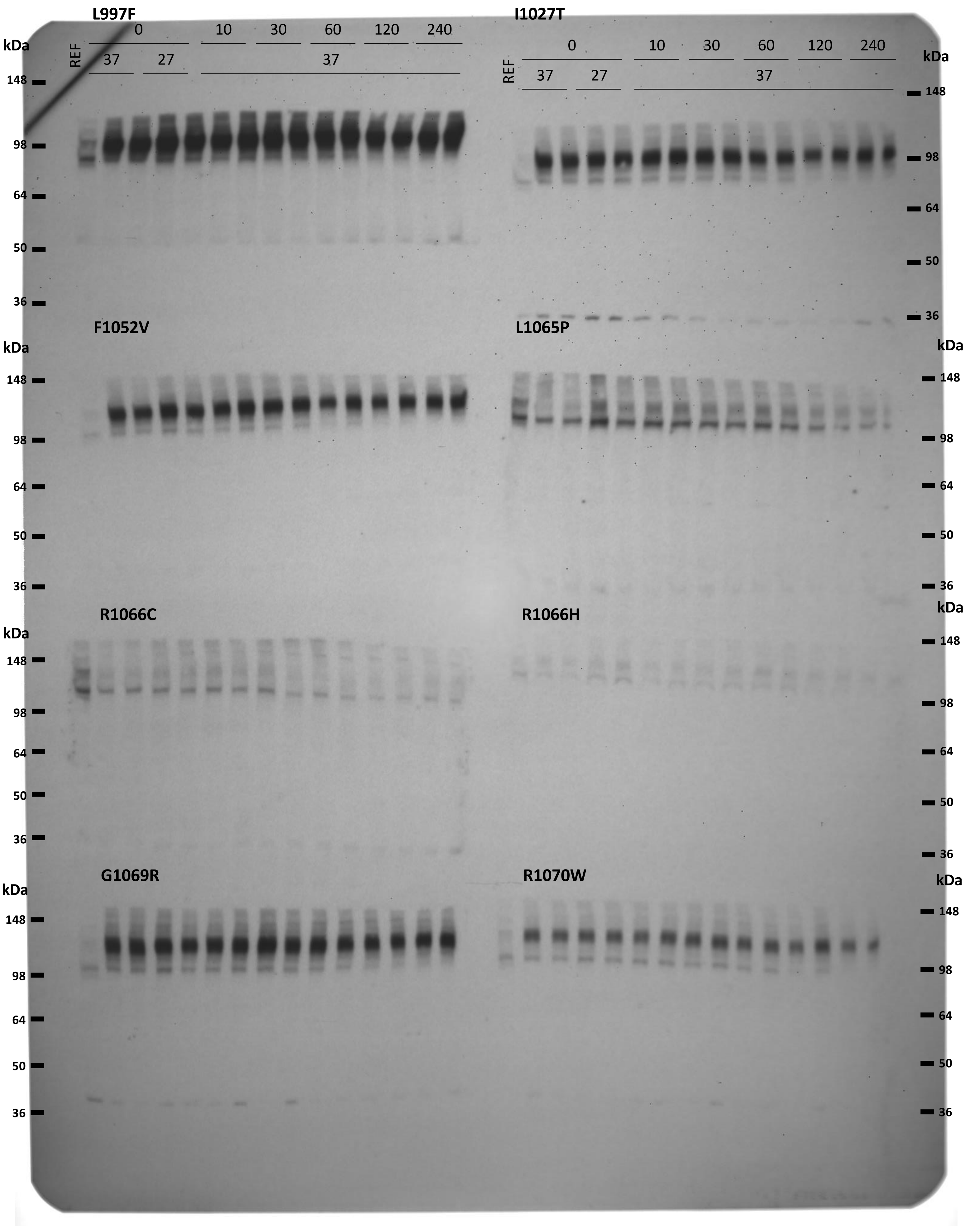

Supplementary Figure 66

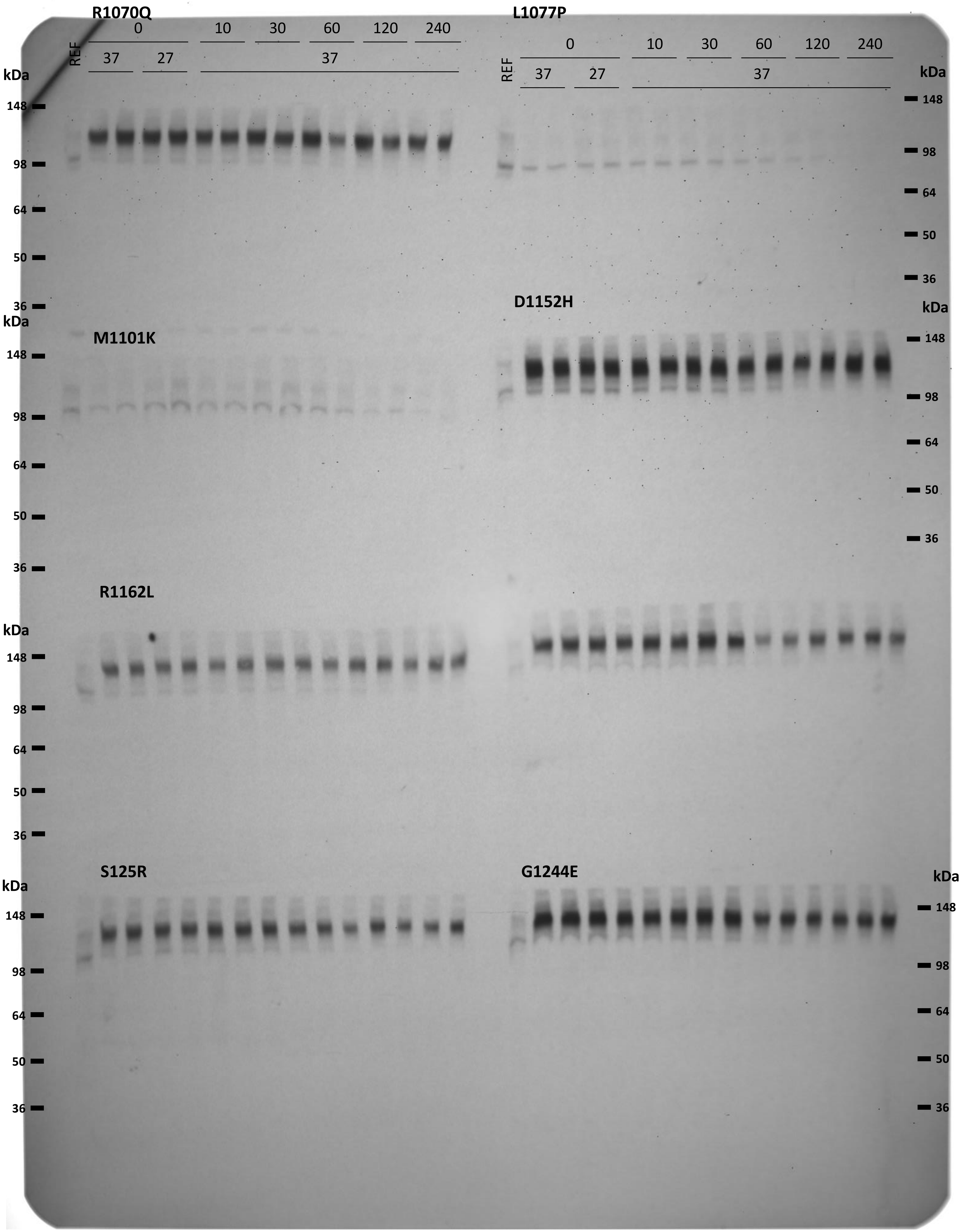

Supplementary Figure 67

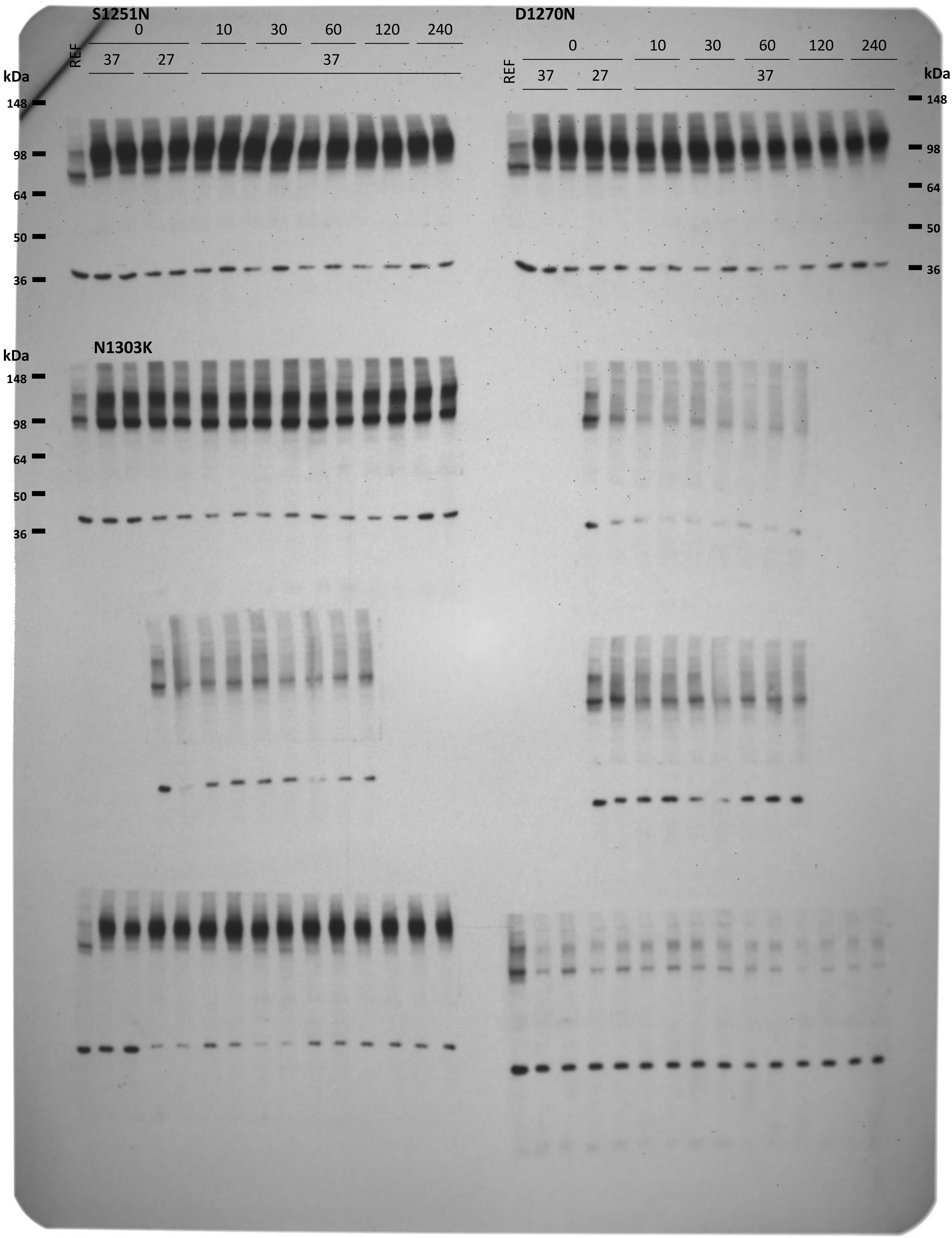

Supplementary Figure 68

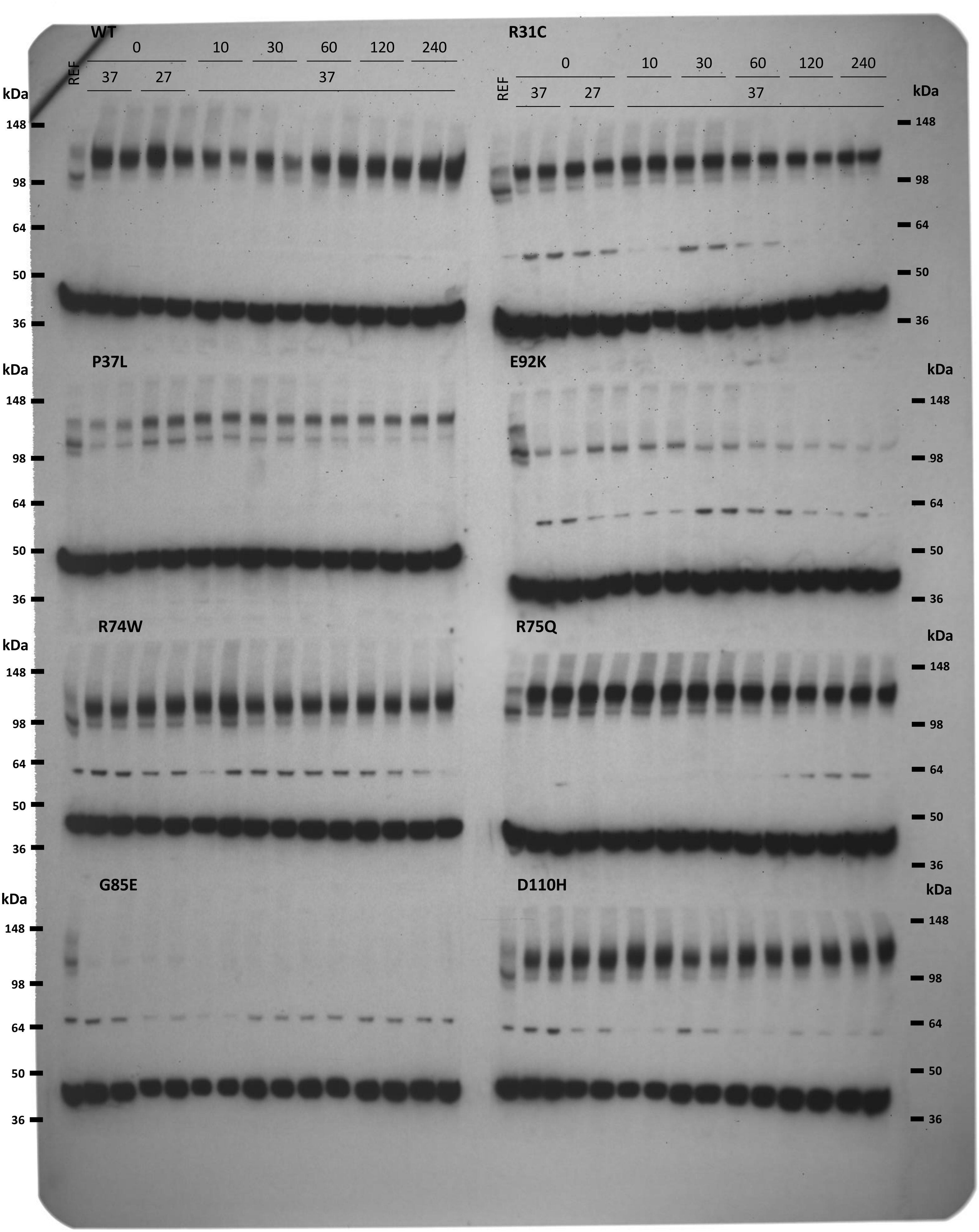

Supplementary Figure 69

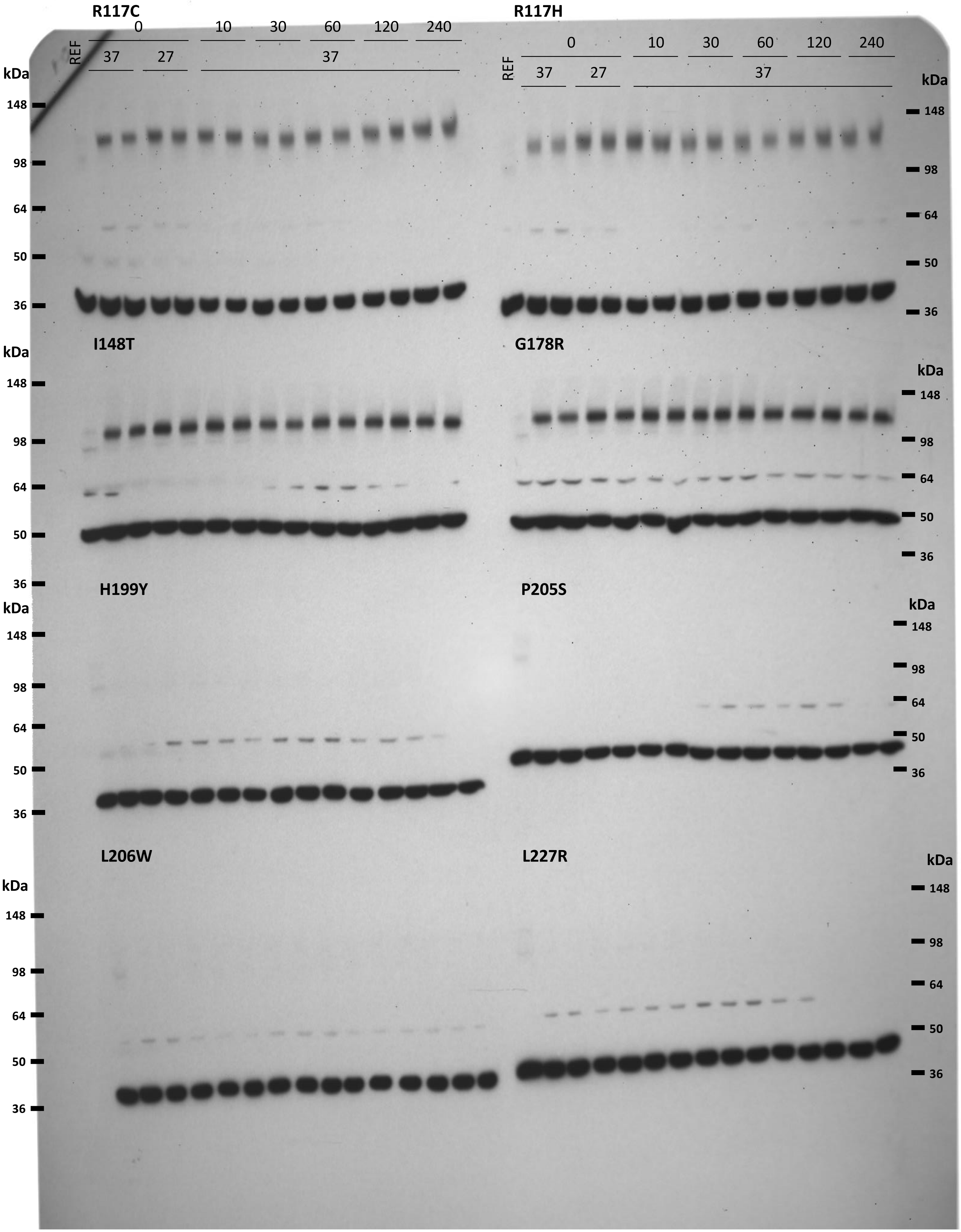

Supplementary Figure 70

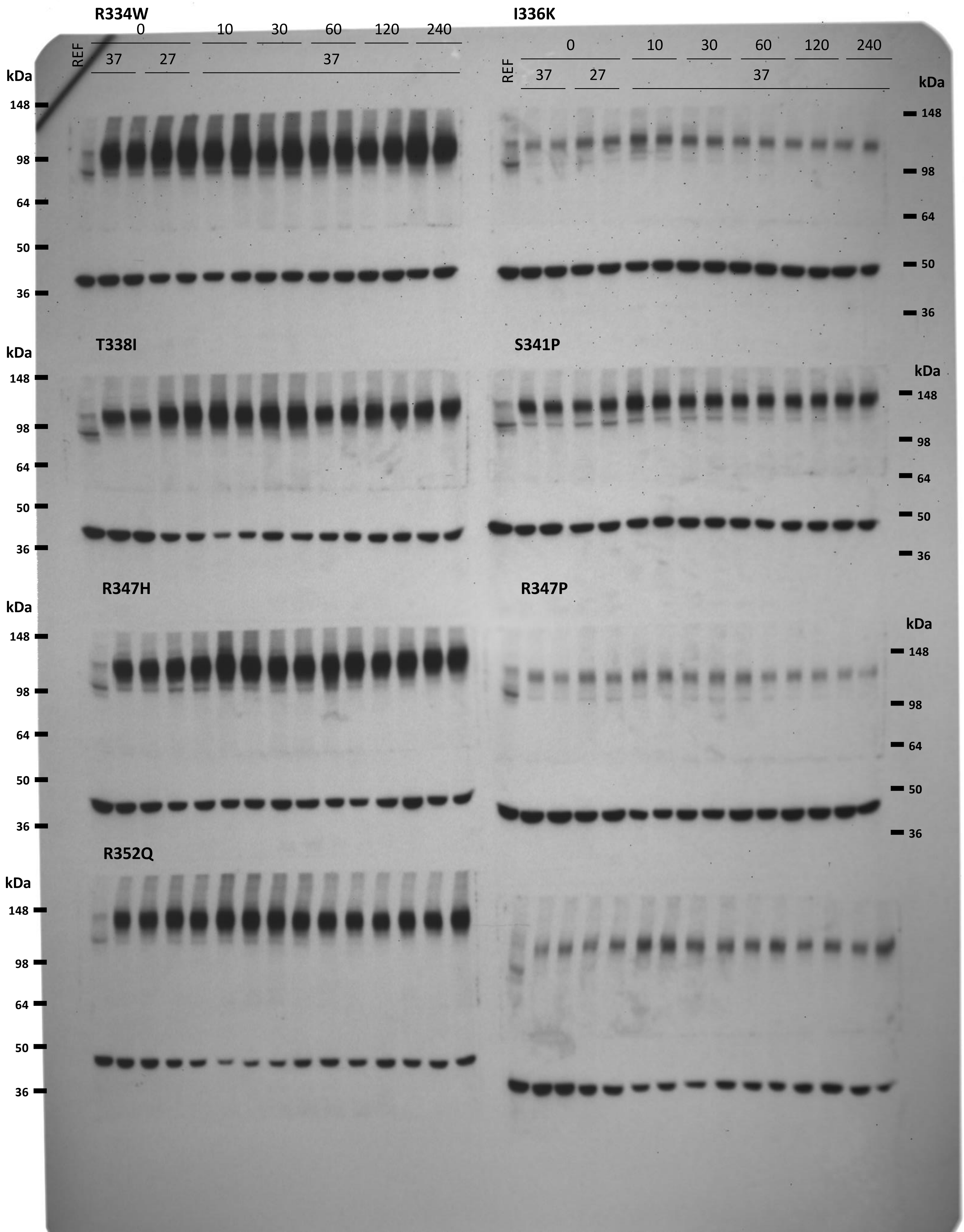

Supplementary Figure 71

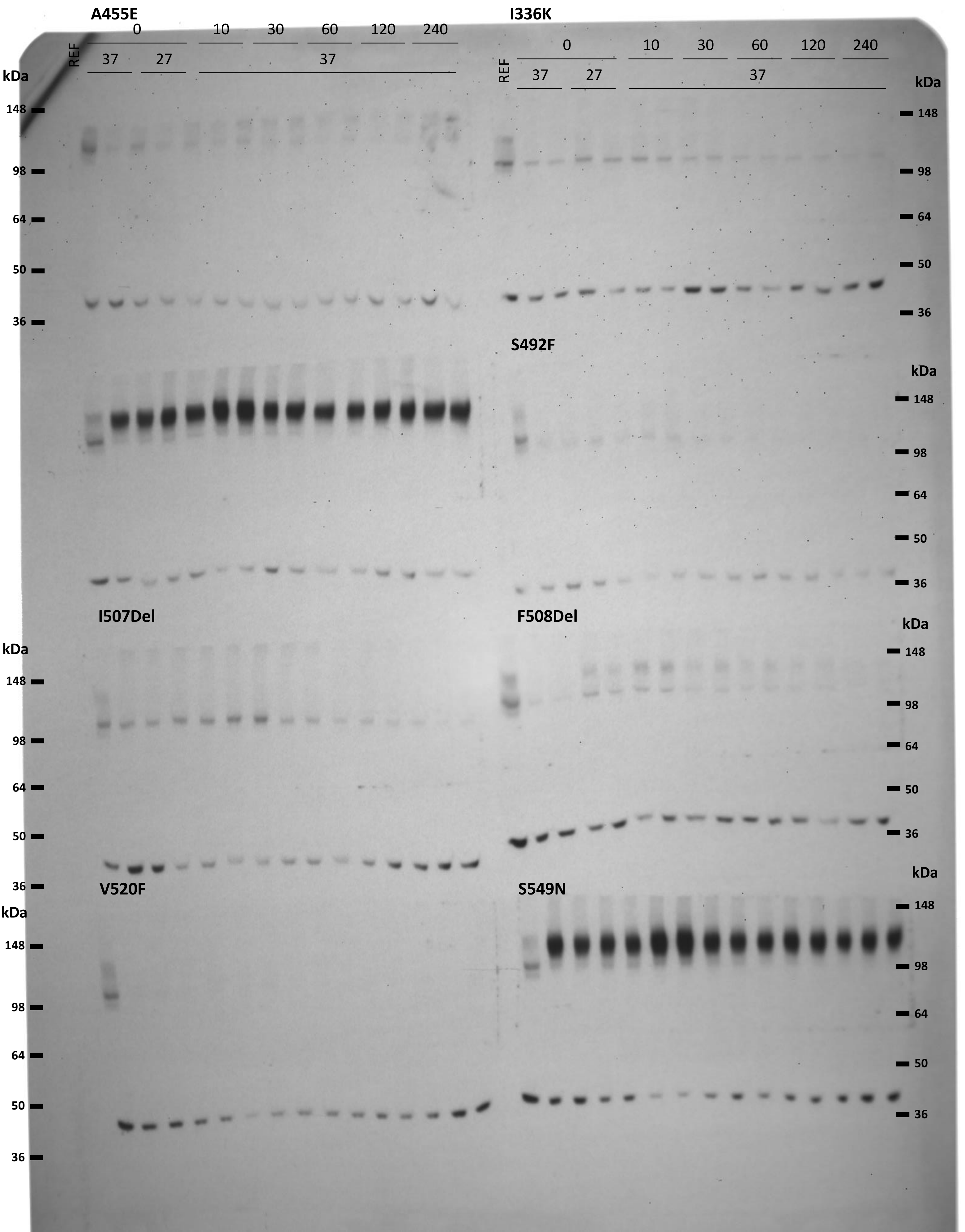

Supplementary Figure 72

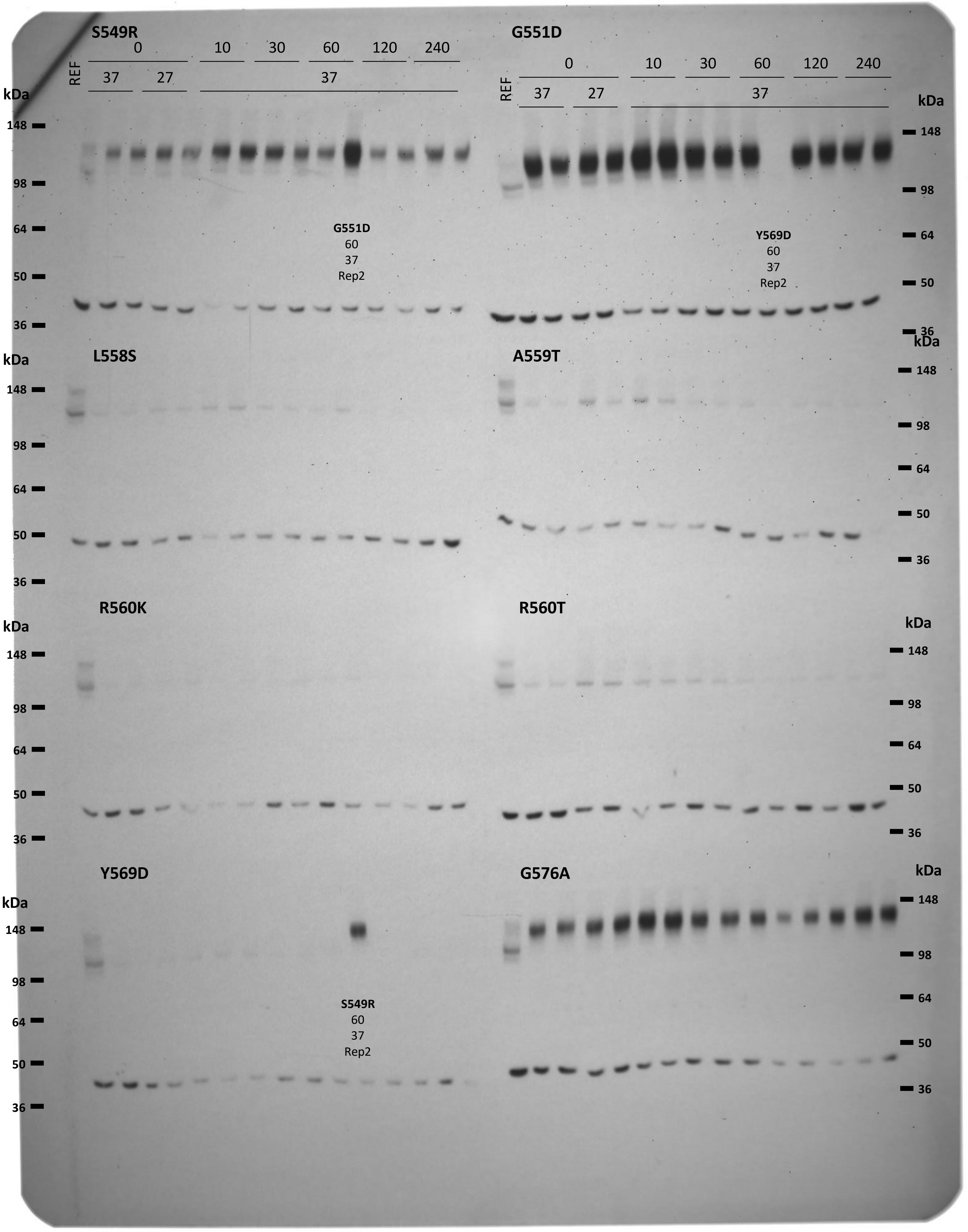

Supplementary Figure 73

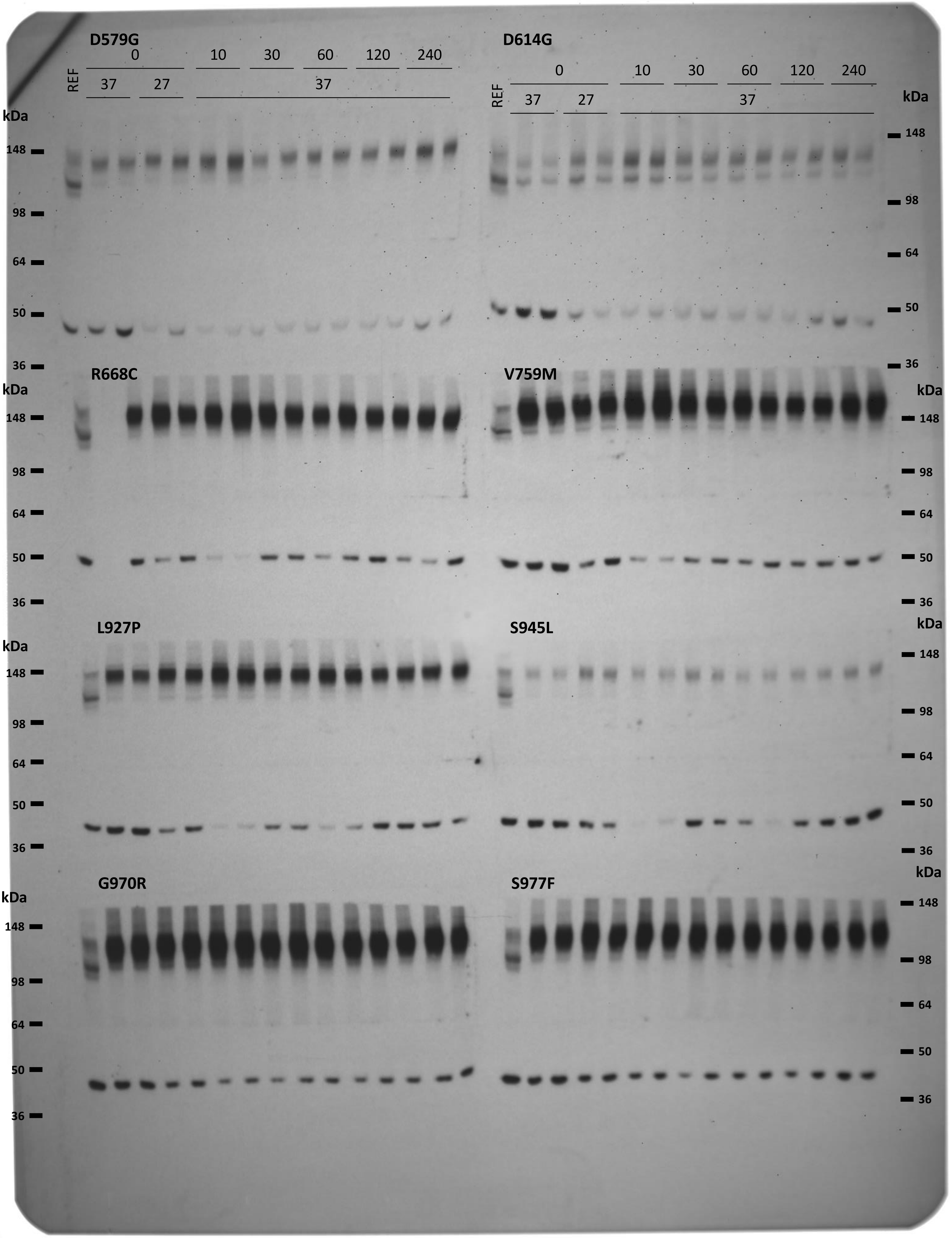

Supplementary Figure 74

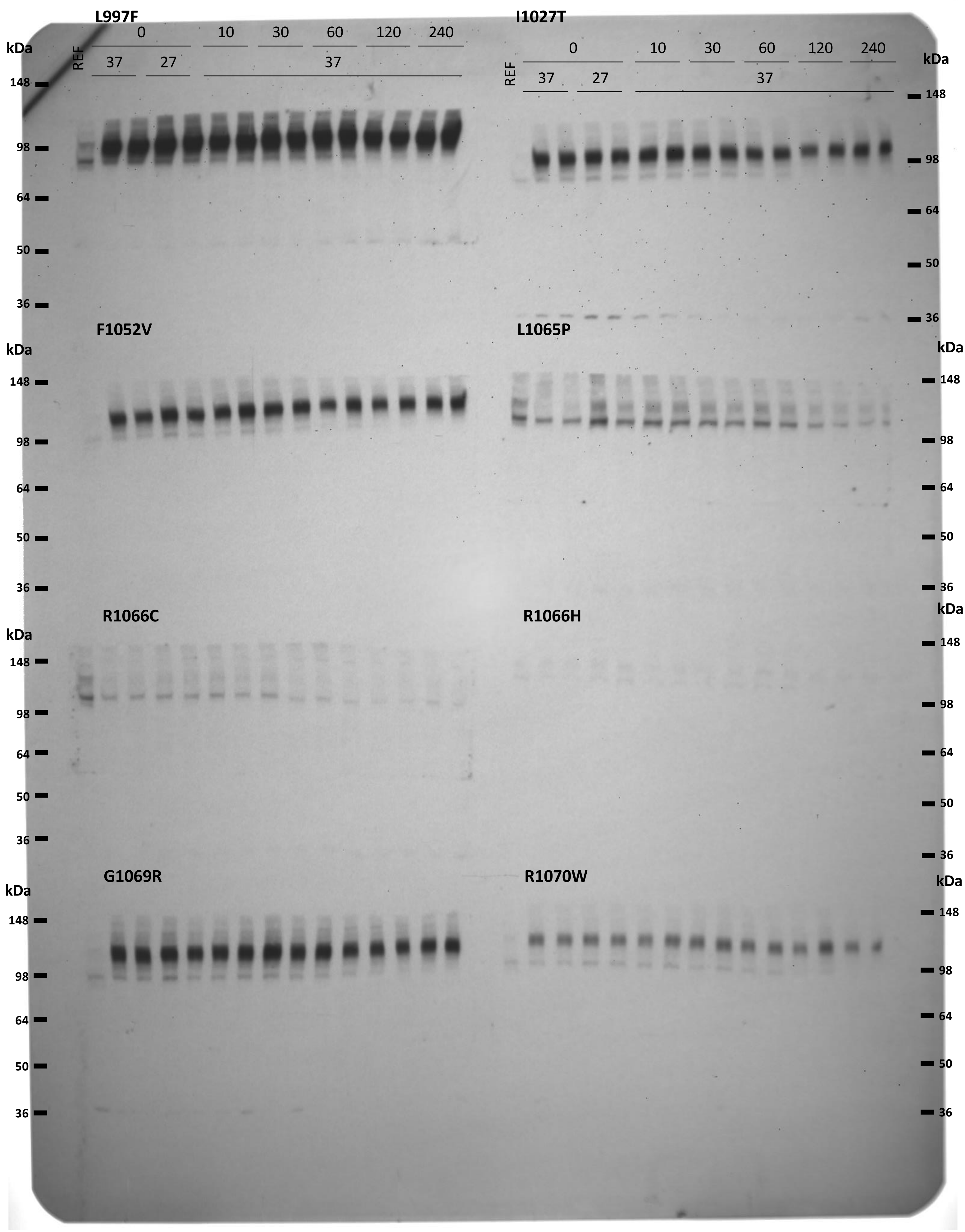

Supplementary Figure 75

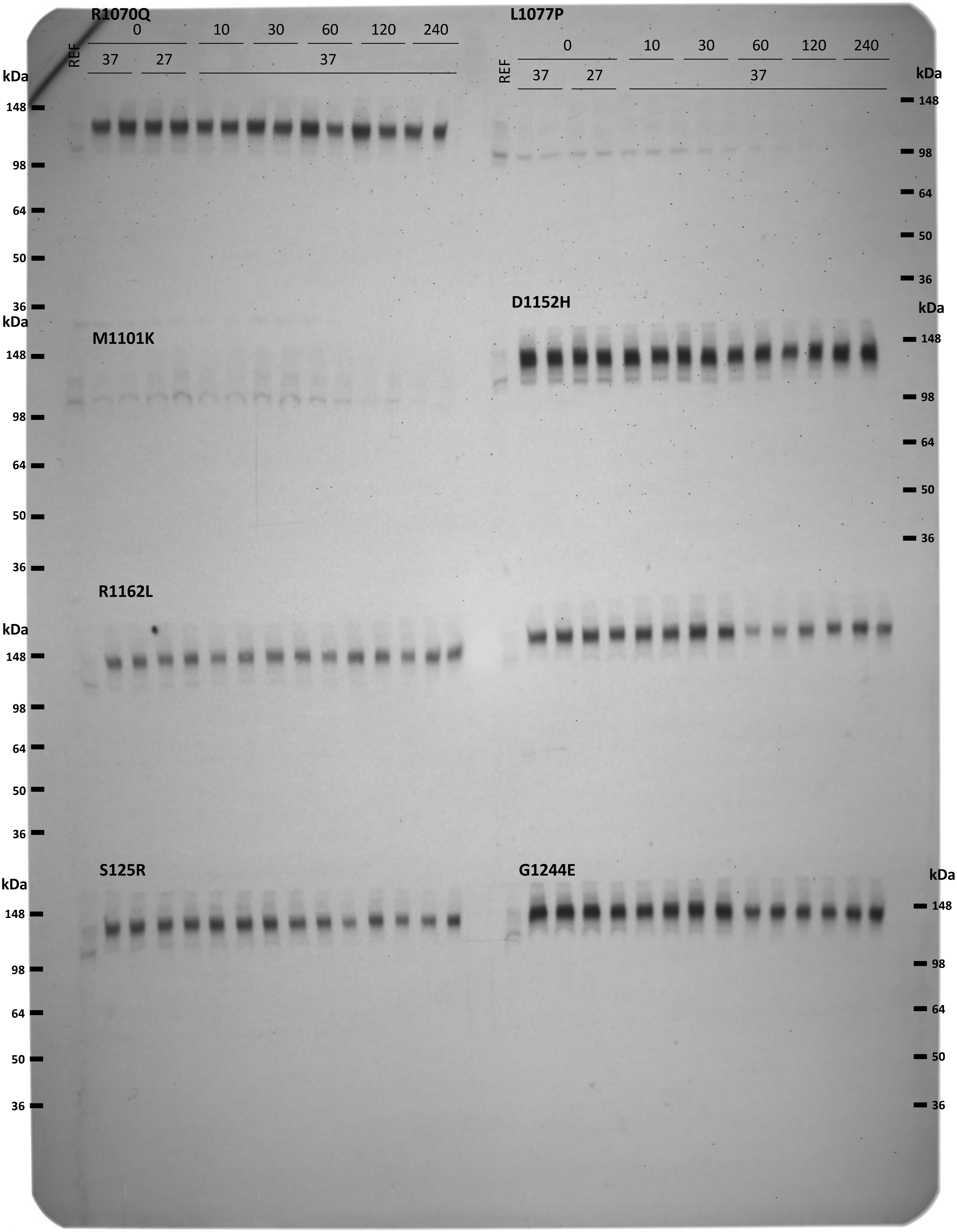

Supplementary Figure 76

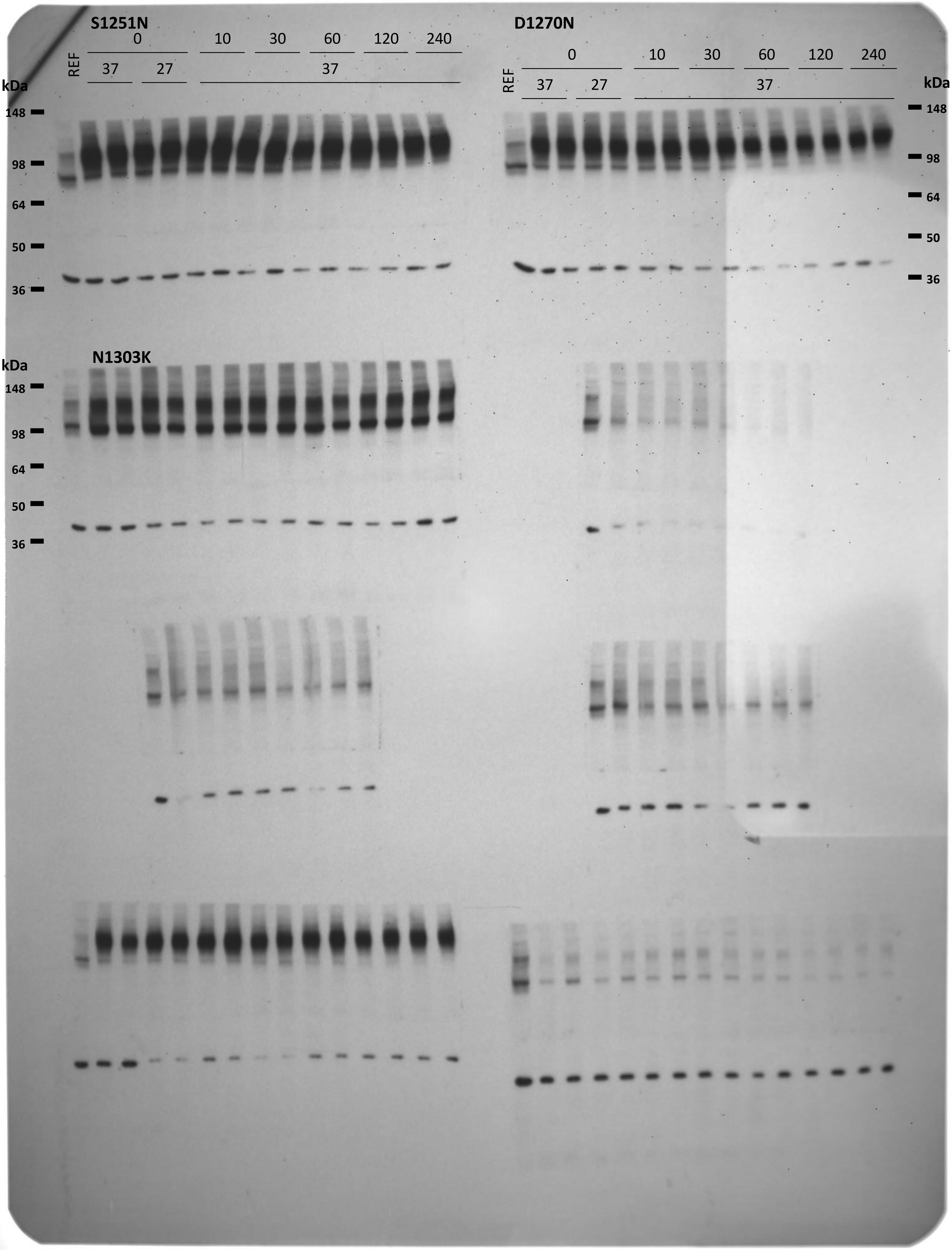

Supplementary Figure 77

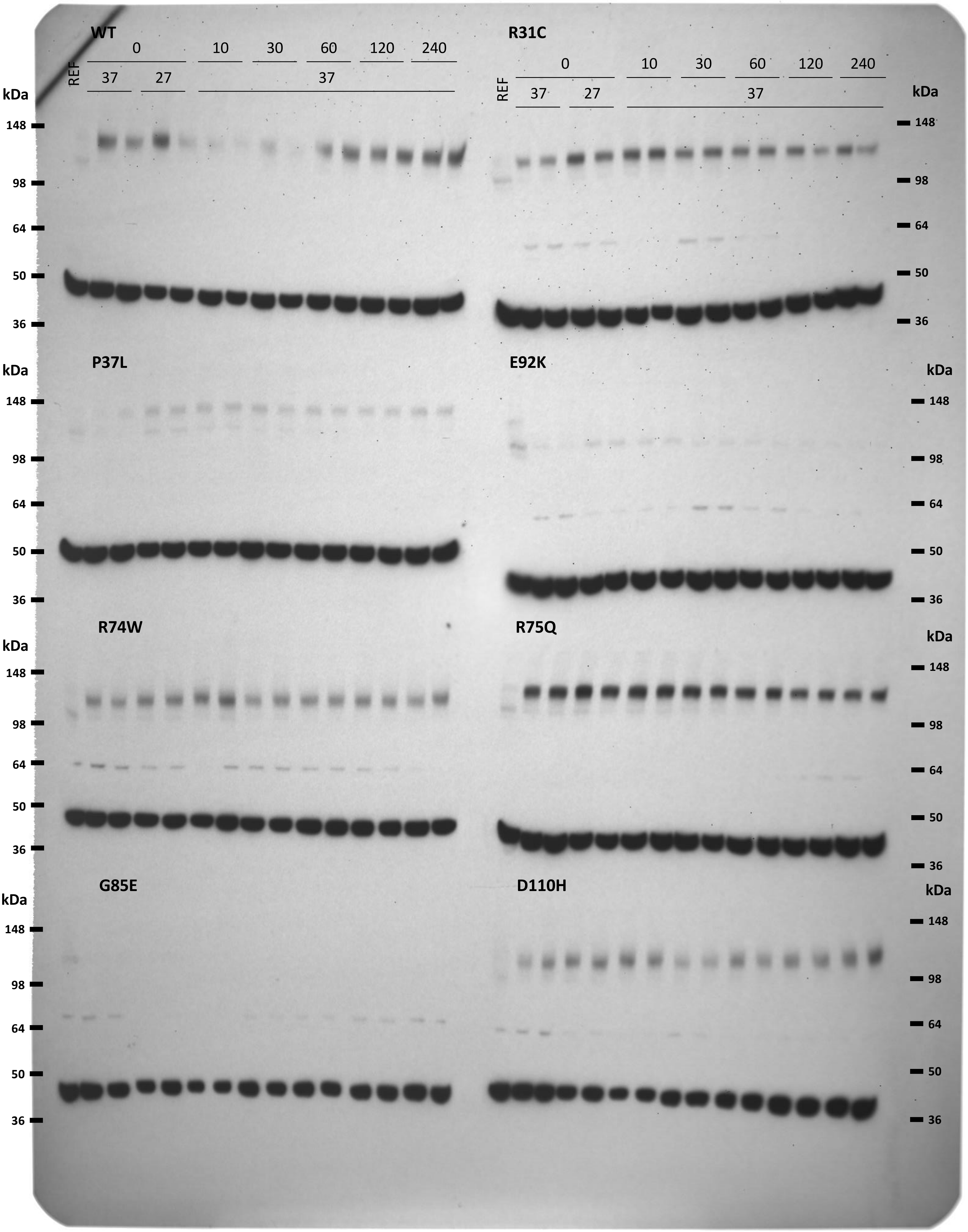

Supplementary Figure 78

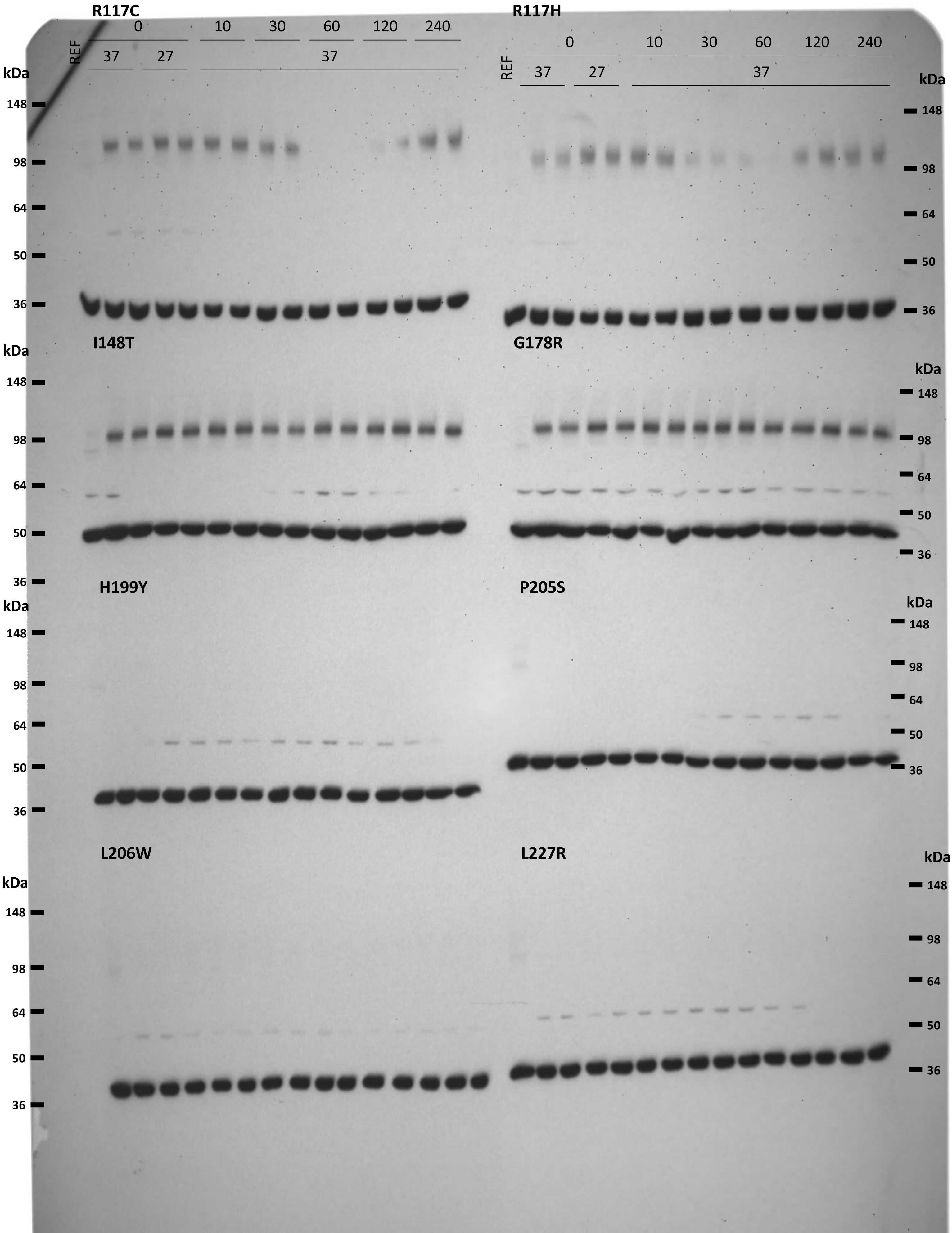

Supplementary Figure 79

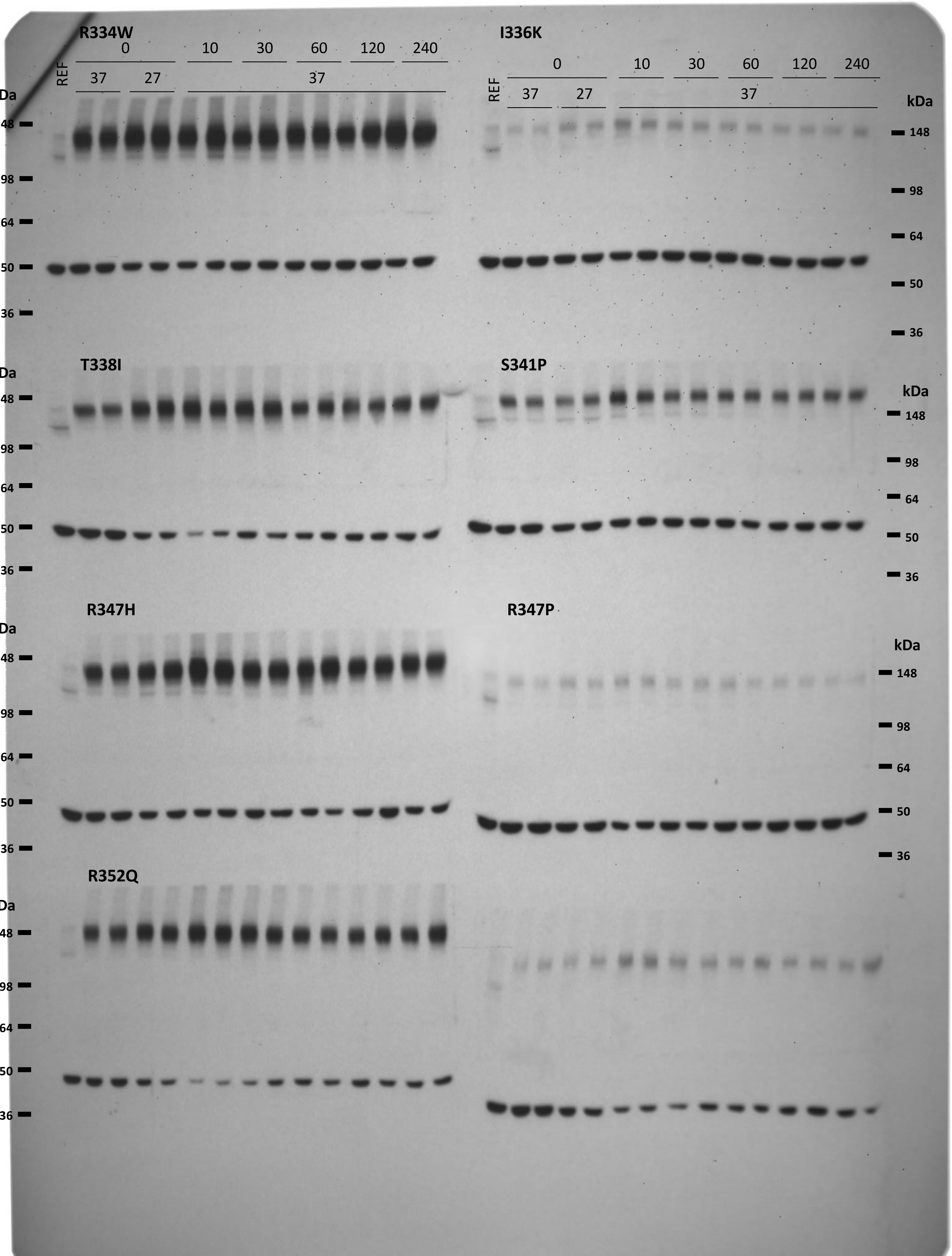

Supplementary Figure 80

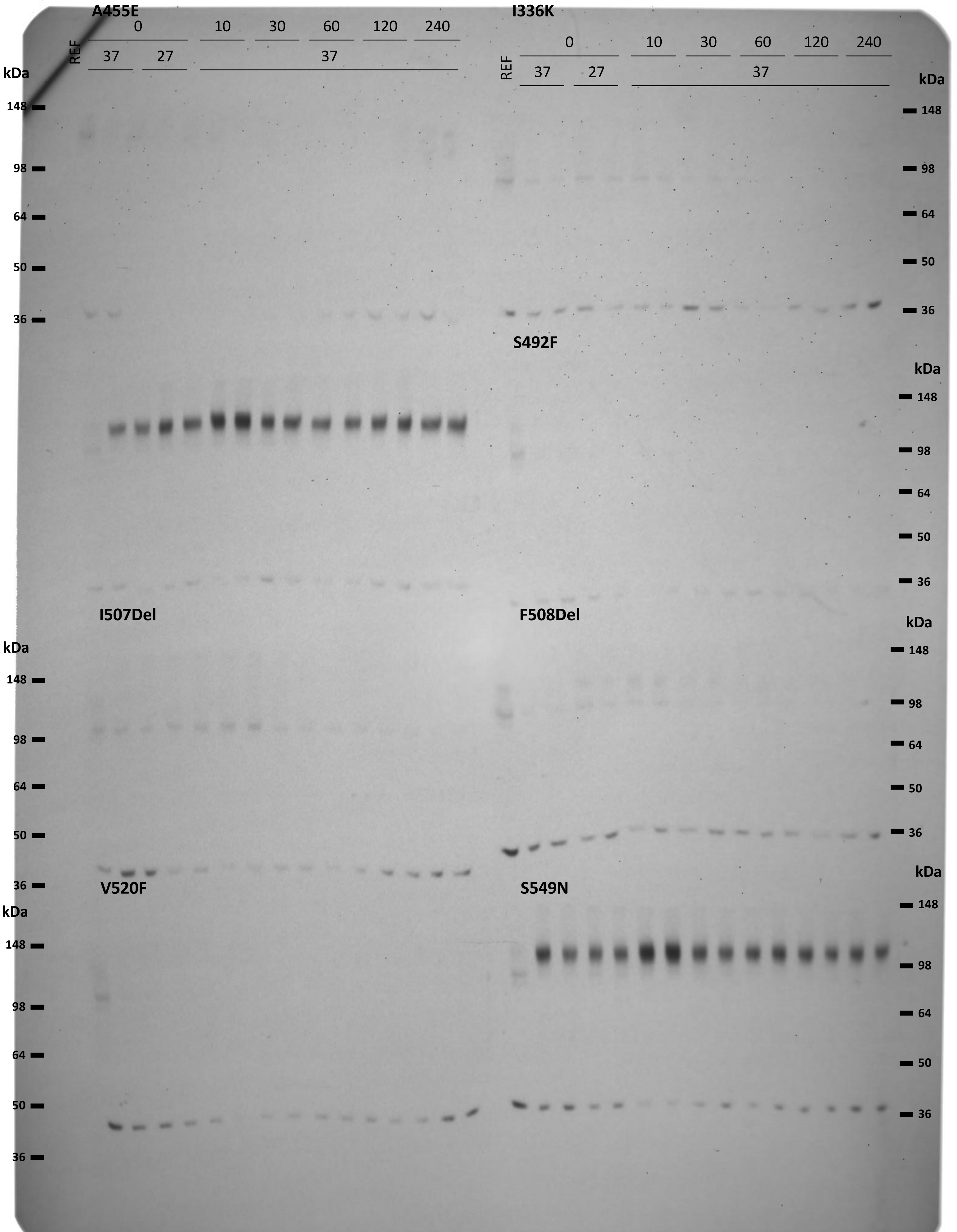

Supplementary Figure 81

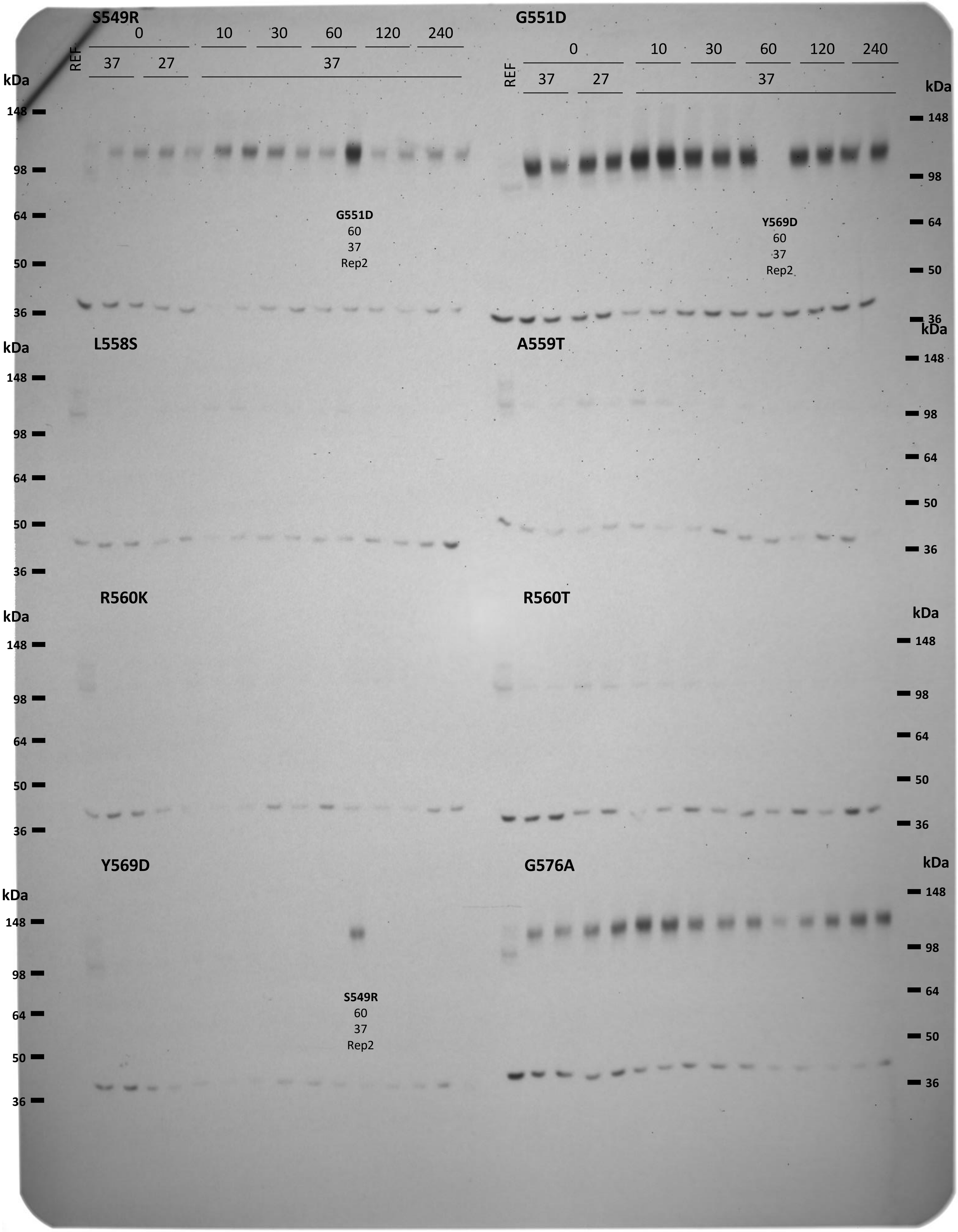

Supplementary Figure 82

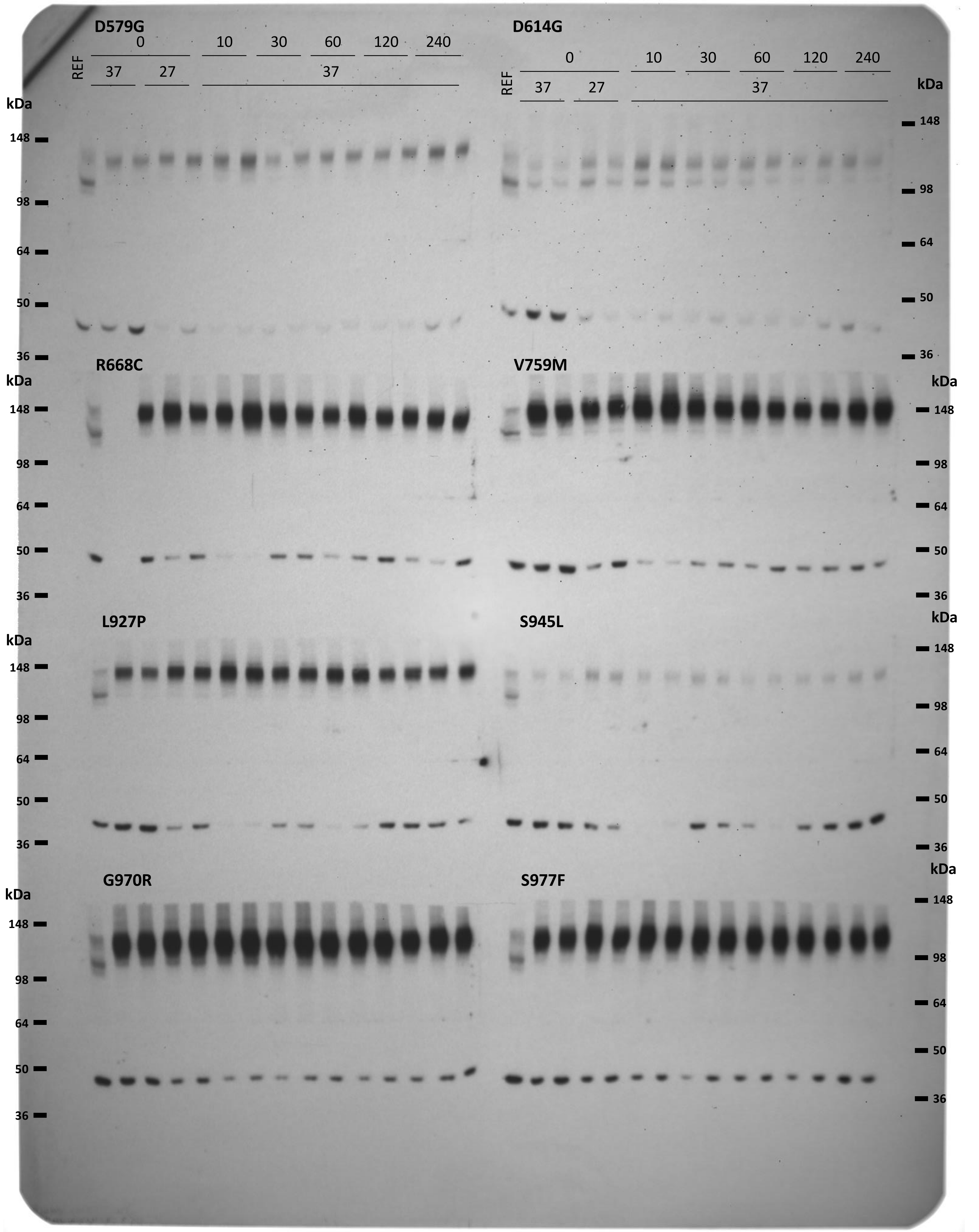

Supplementary Figure 83

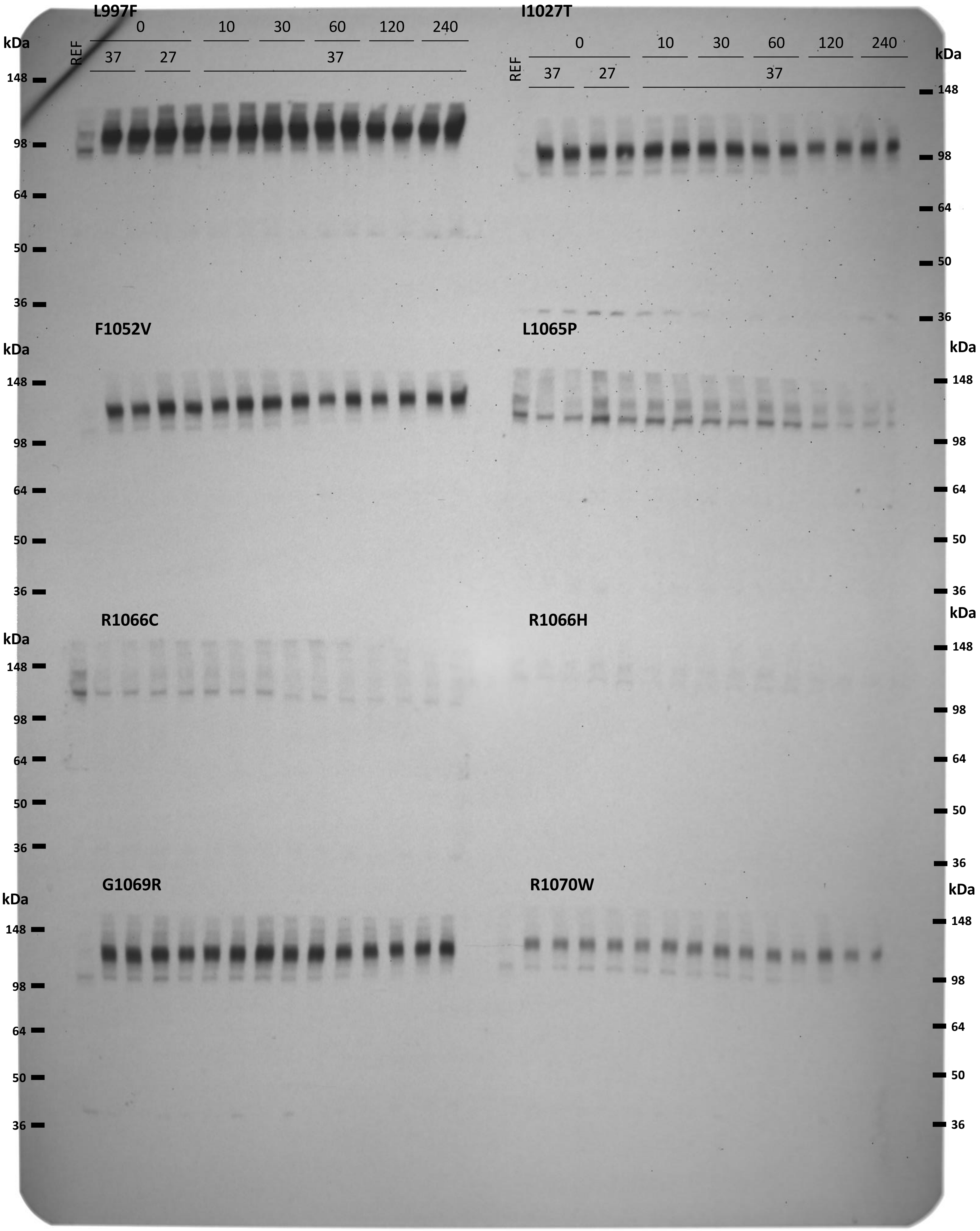

Supplementary Figure 84

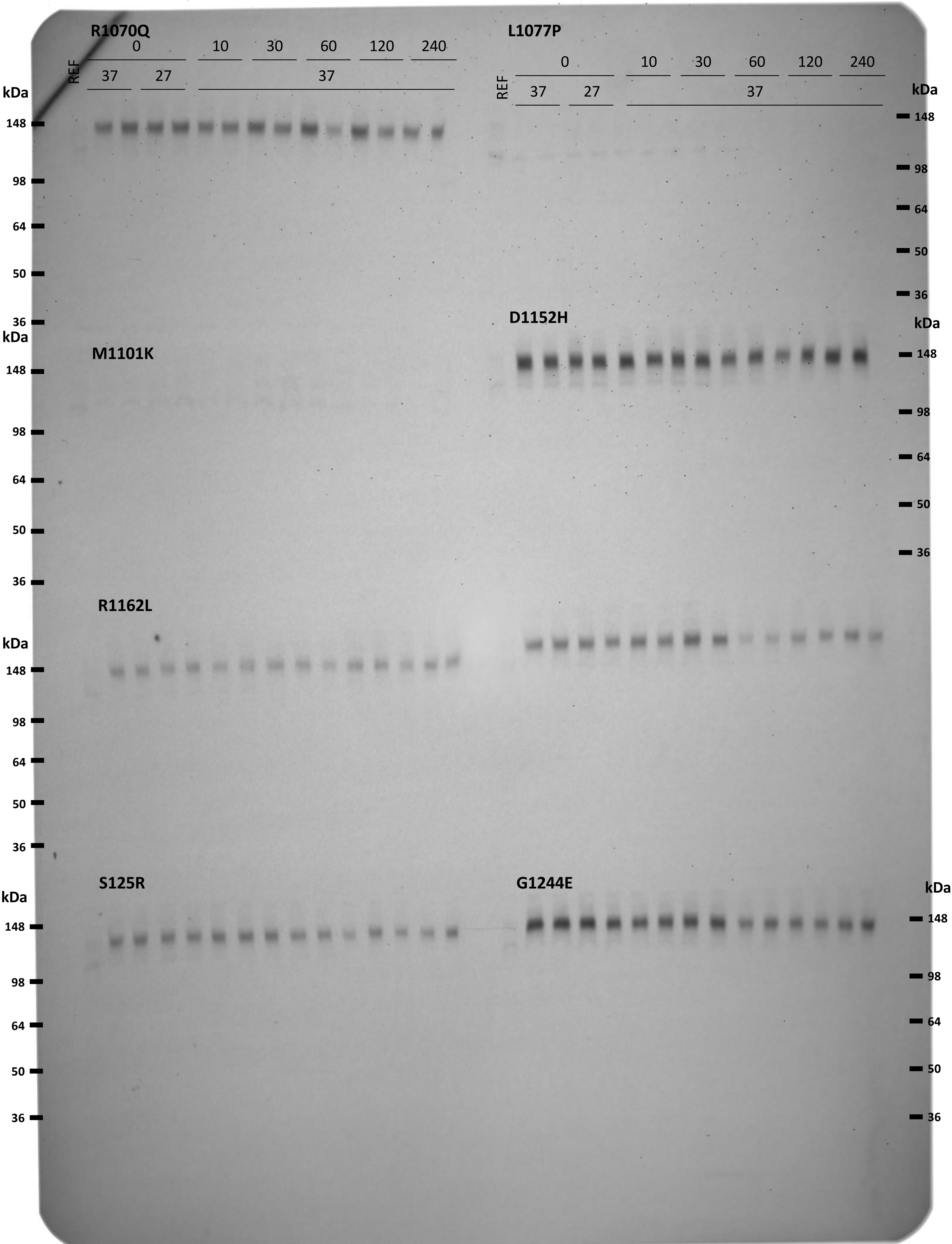

Supplementary Figure 85

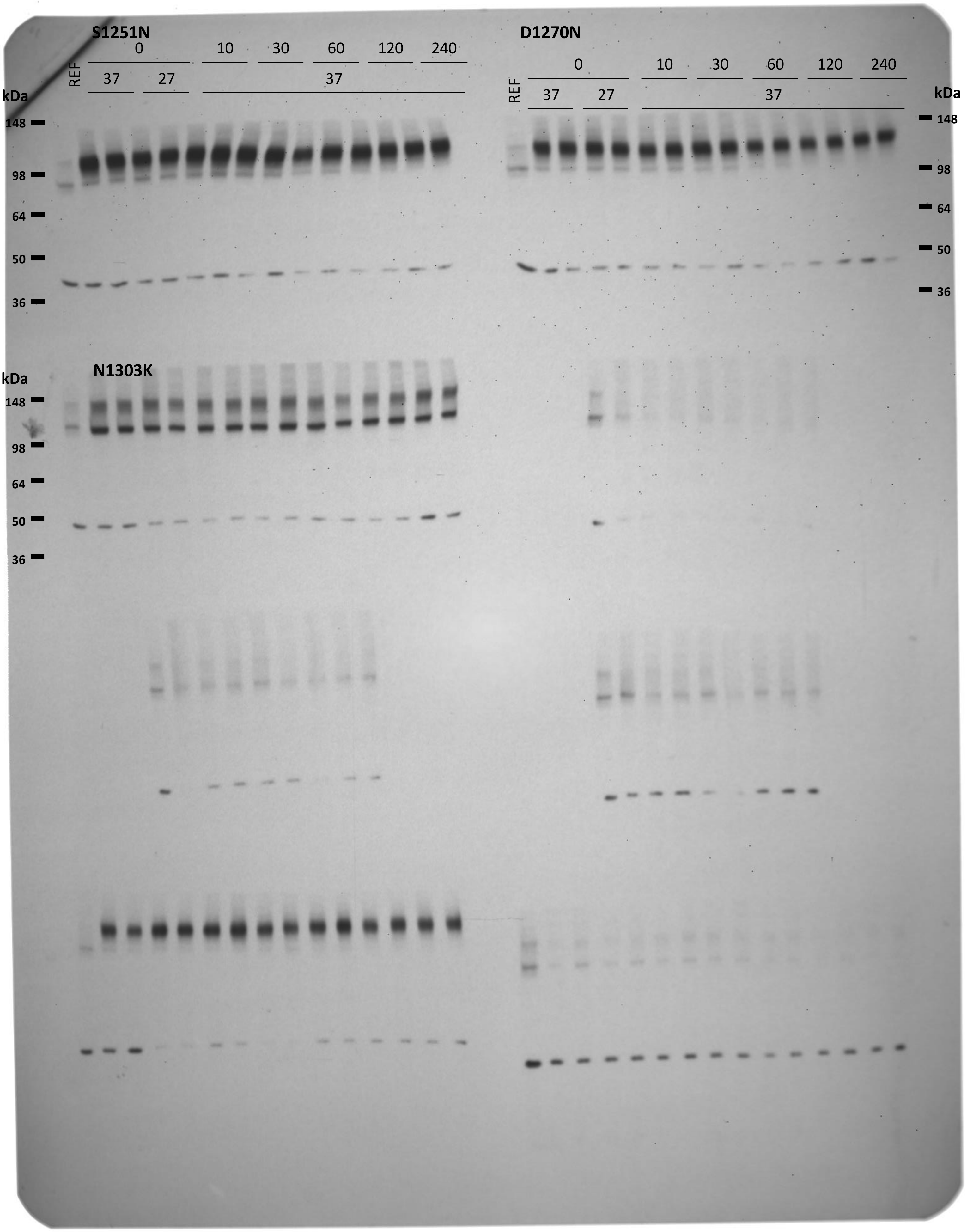

Supplementary Figure 86

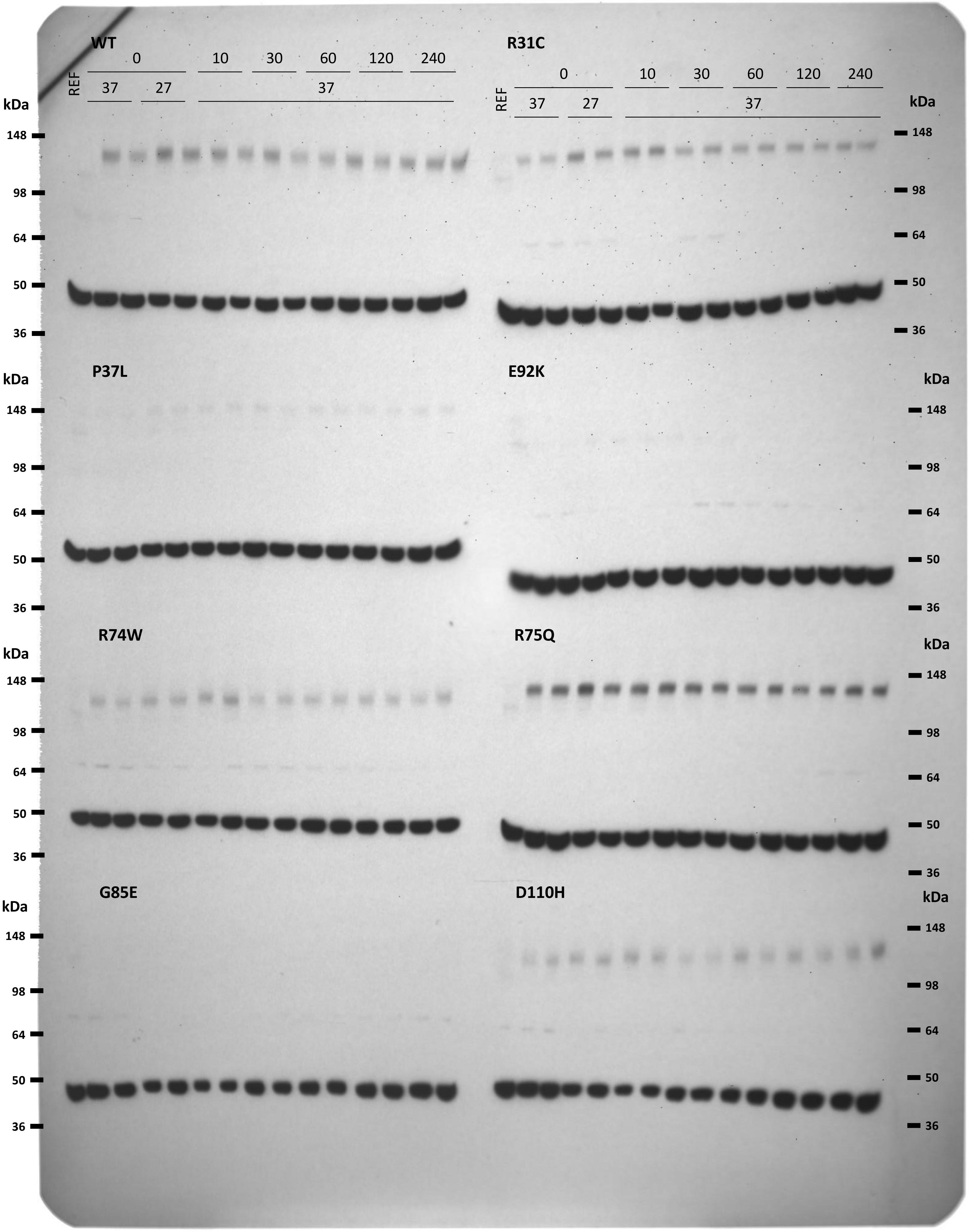

Supplementary Figure 87

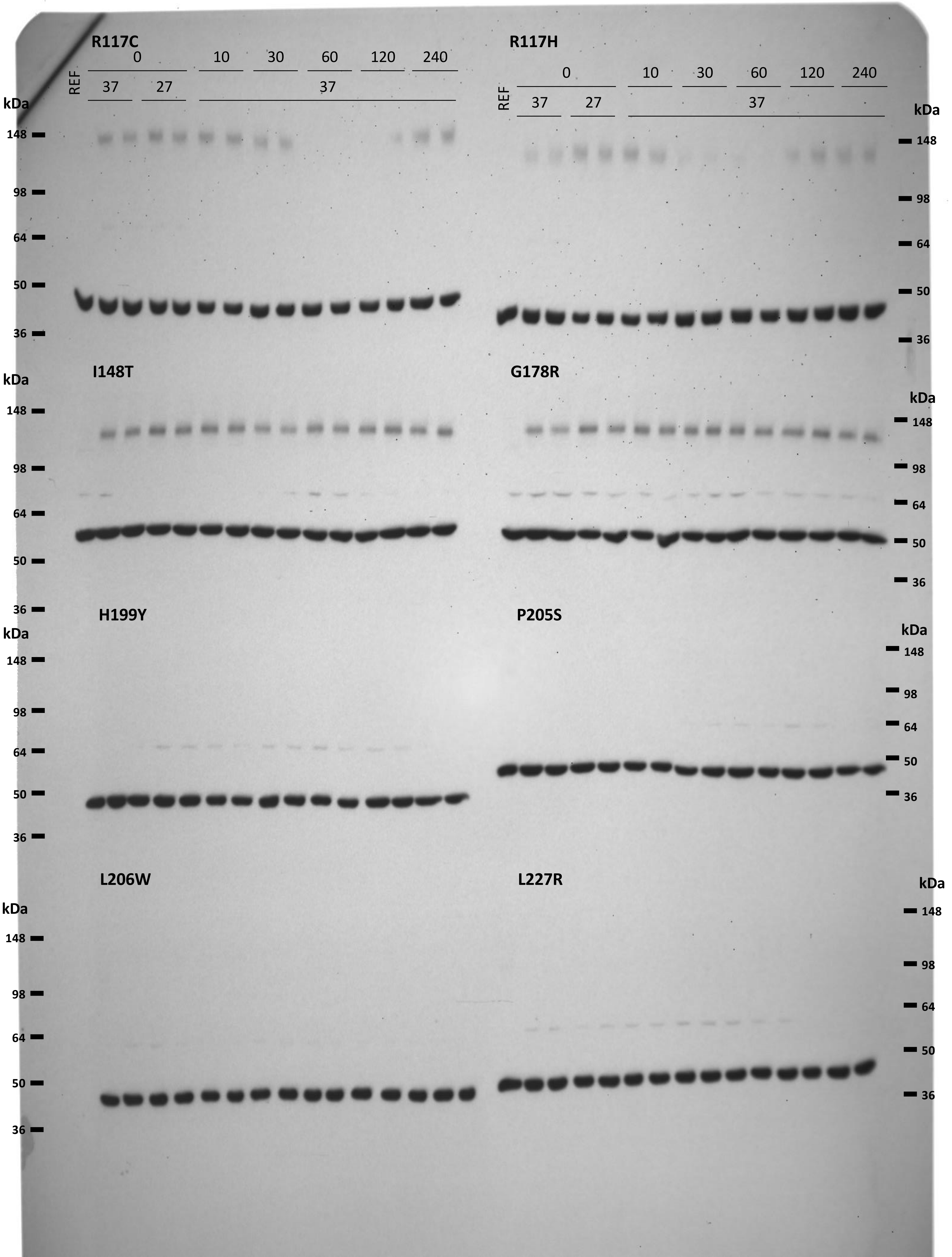

Supplementary Figure 88

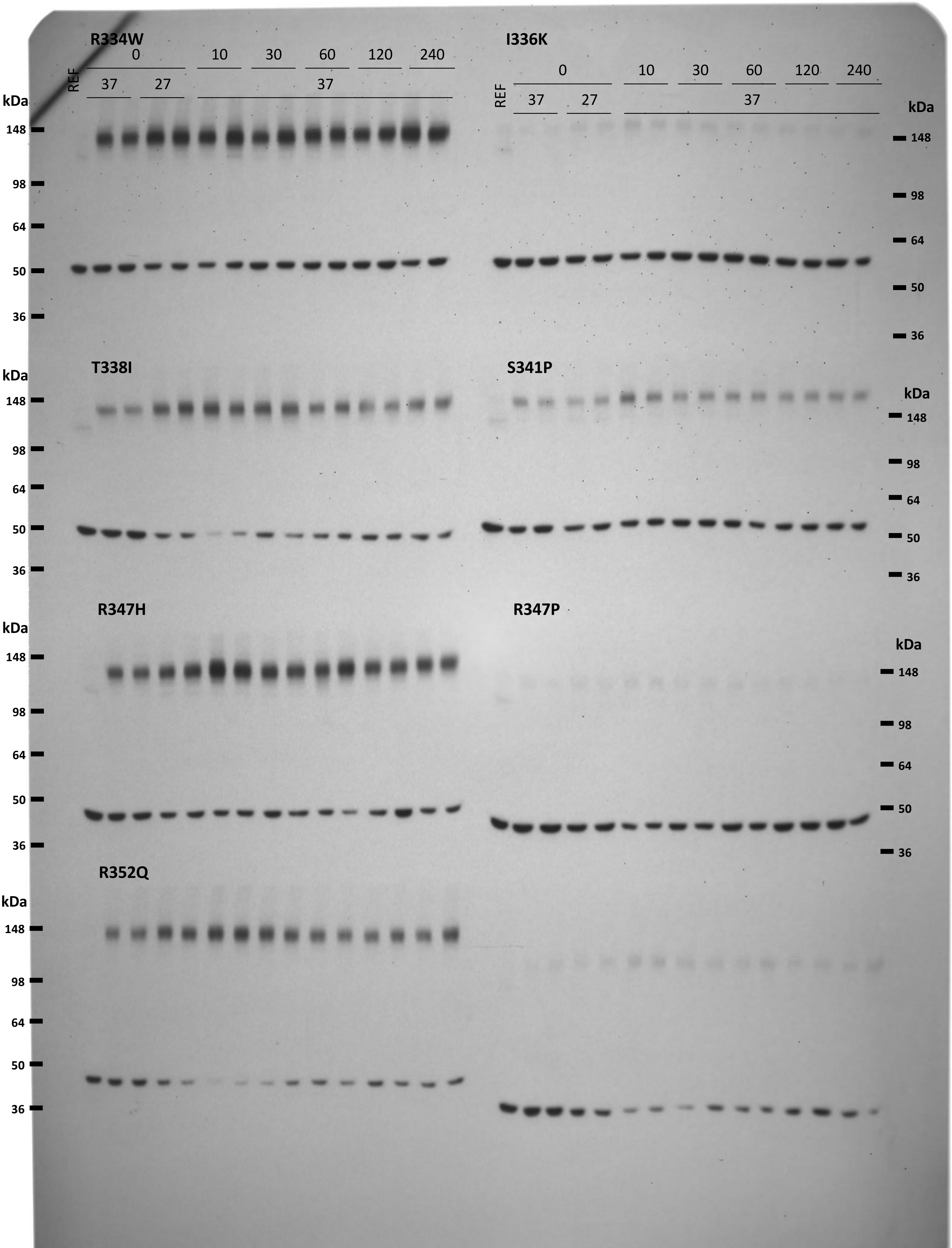

Supplementary Figure 89

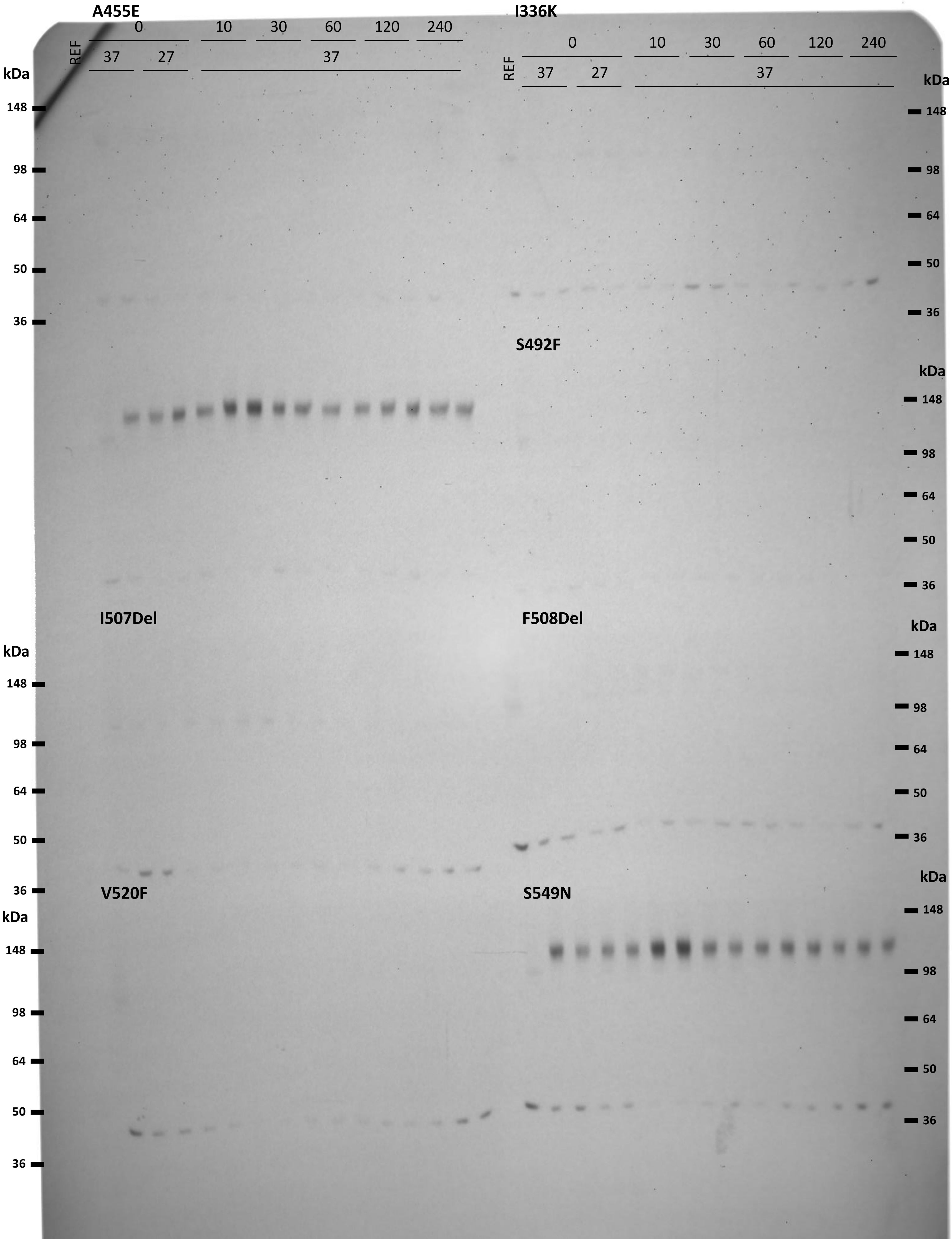

Supplementary Figure 90

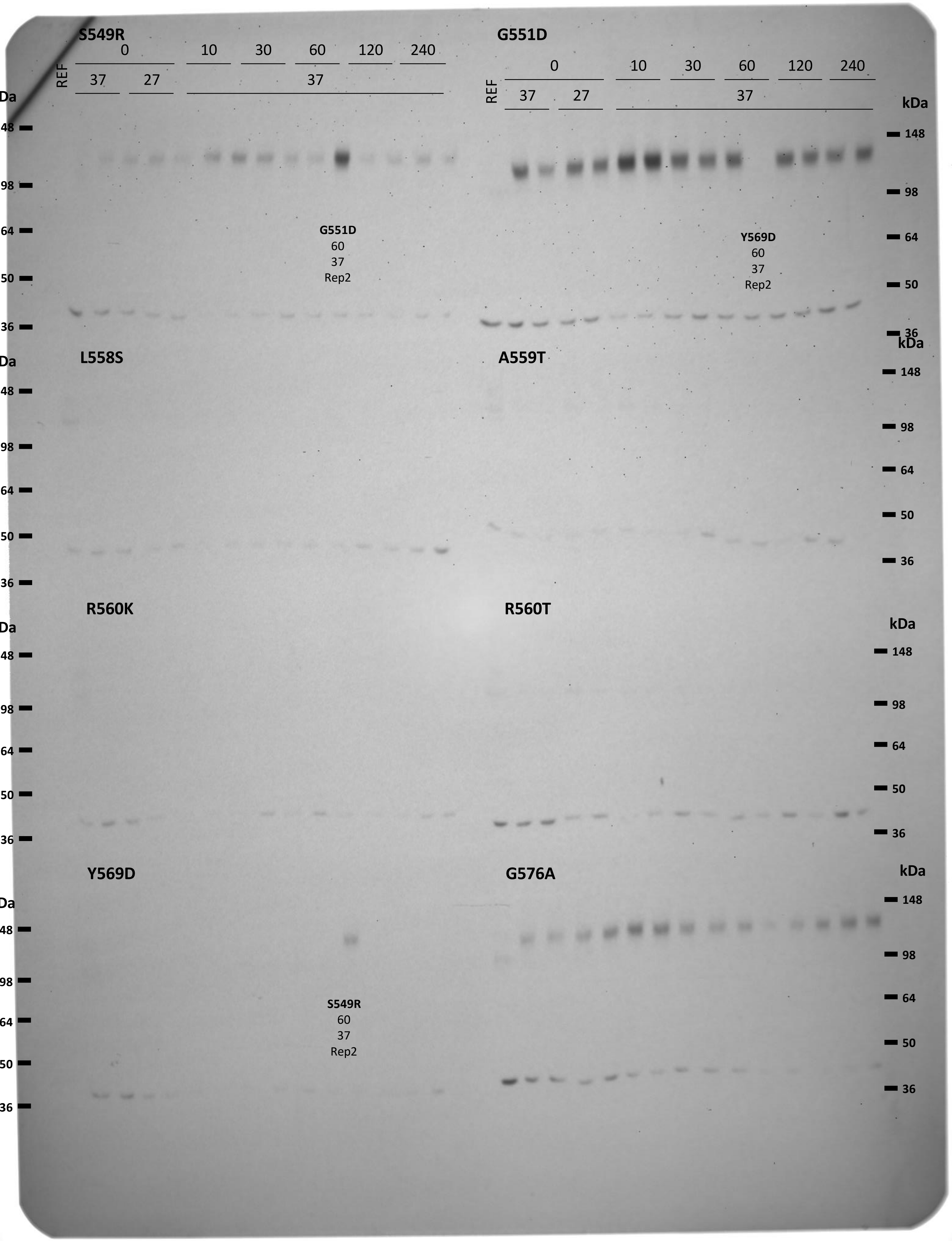

Supplementary Figure 91

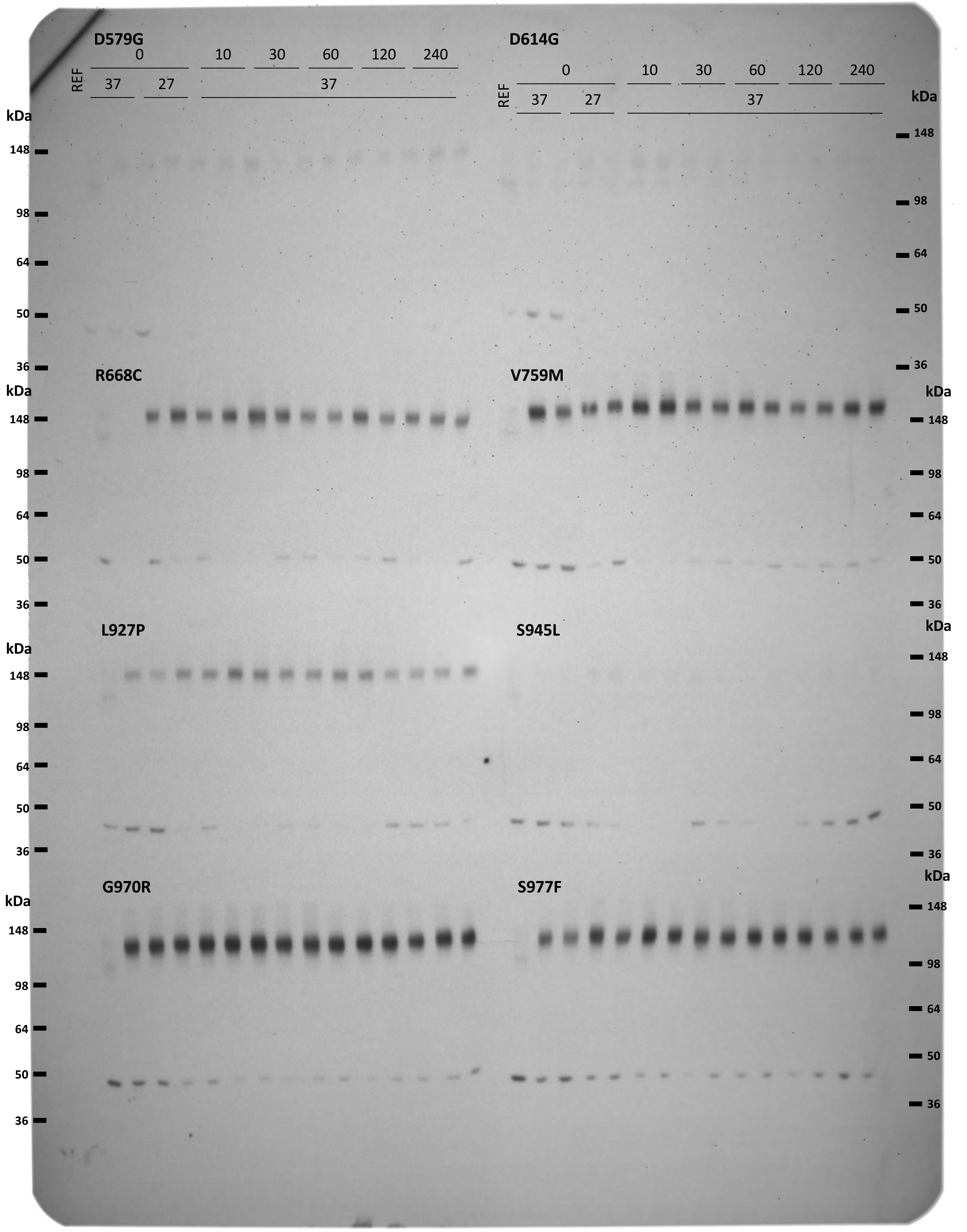

Supplementary Figure 92

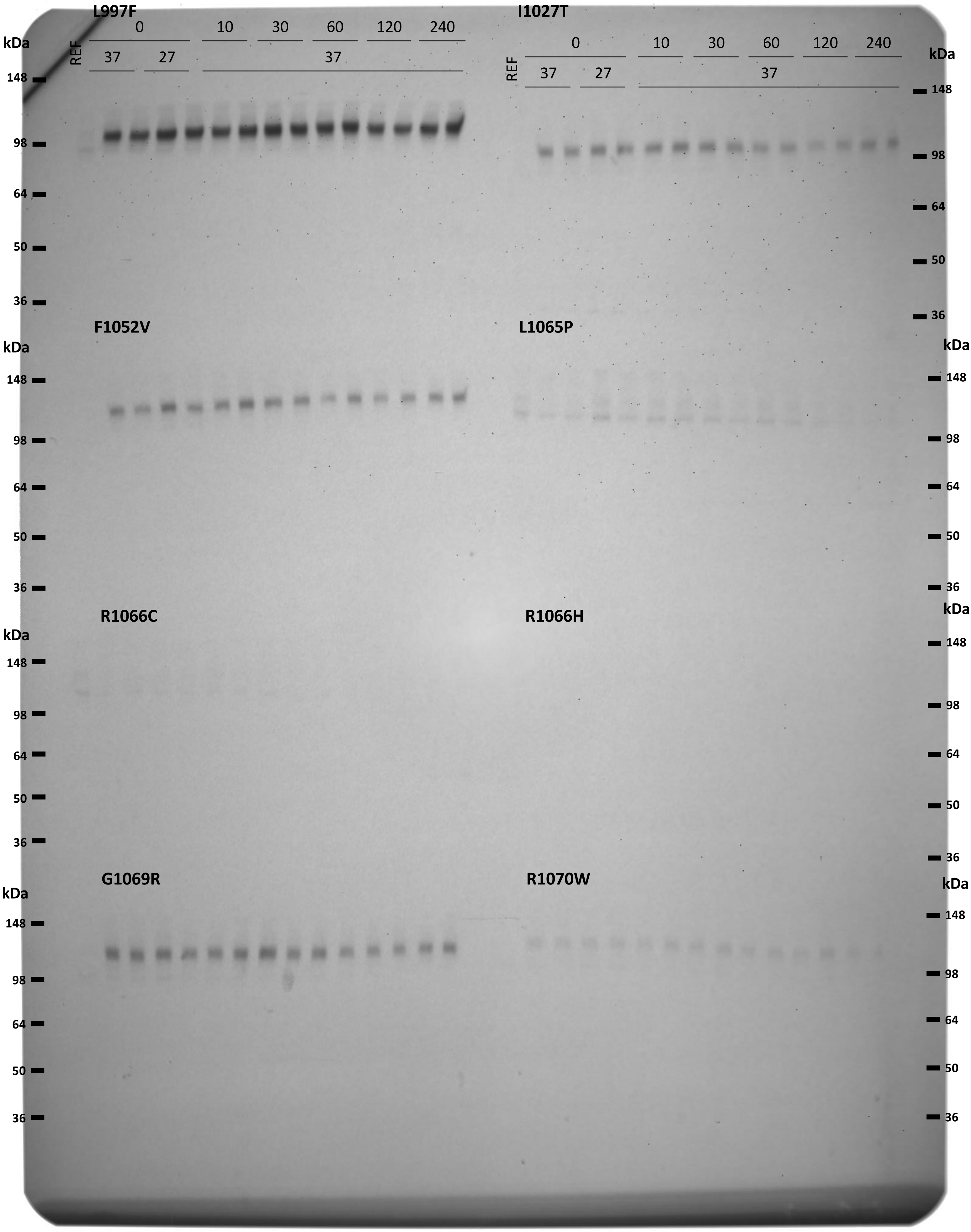

Supplementary Figure 93

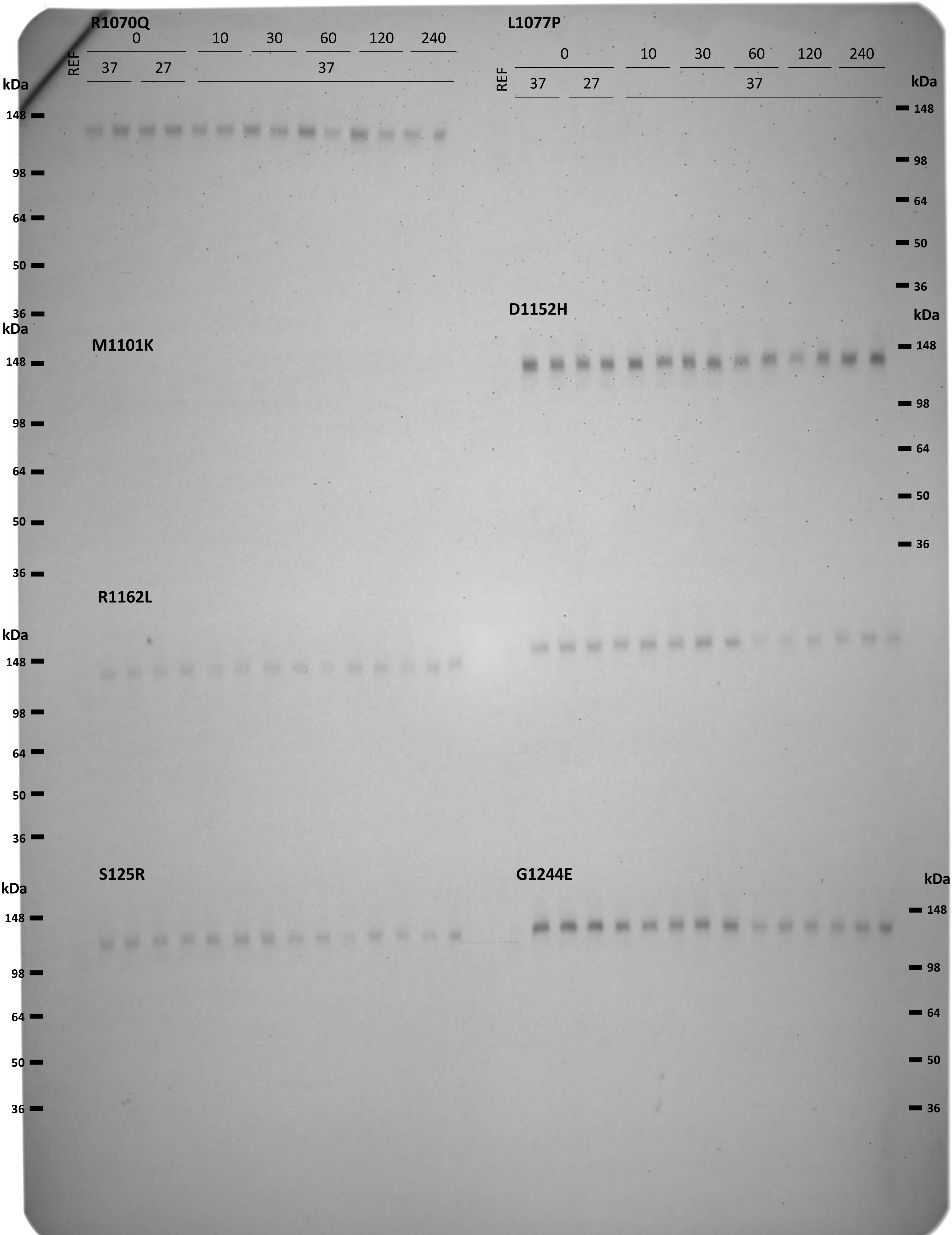

Supplementary Figure 94

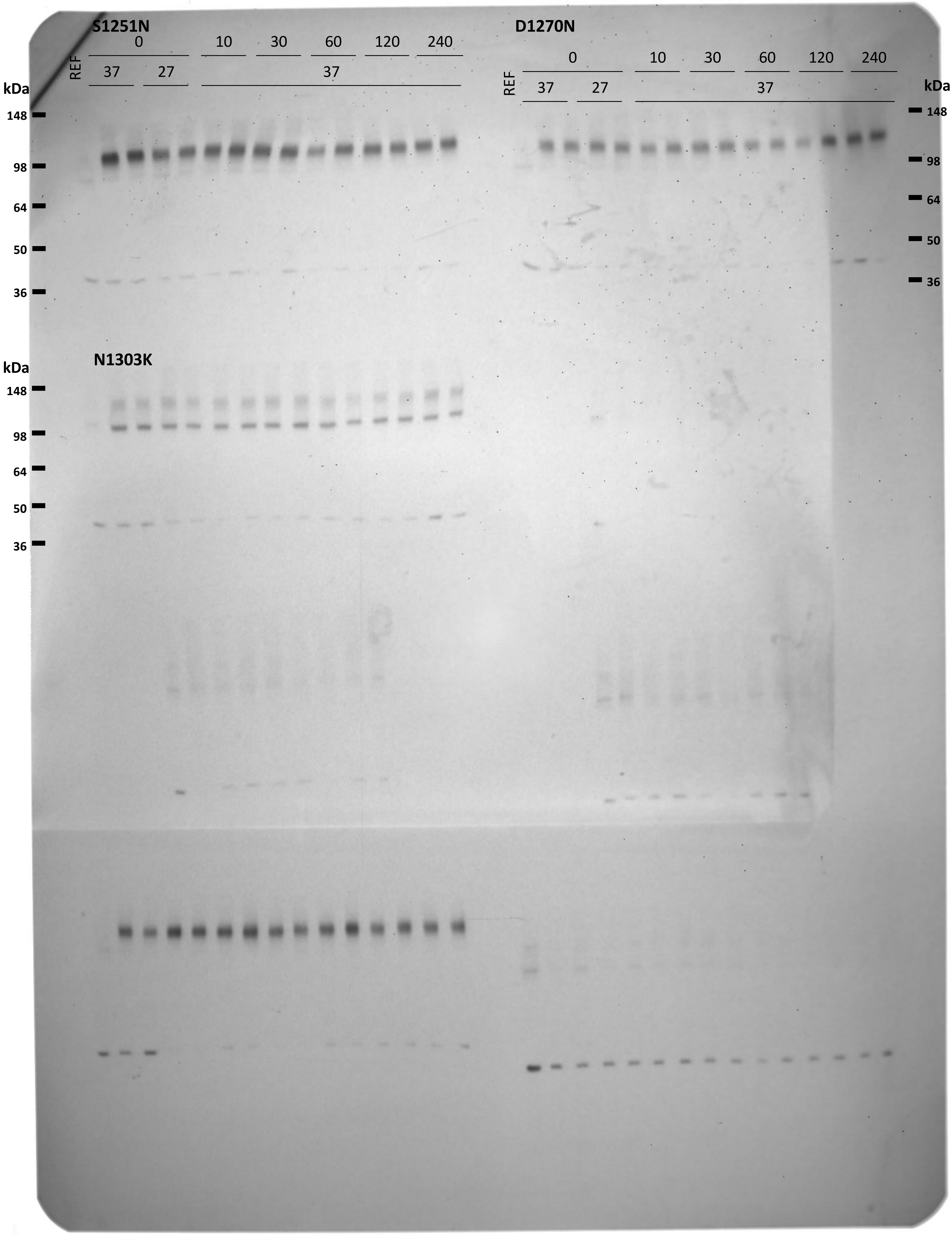

Supplementary Figure Legends

Supplementary Figure 1-7. SDS-PAGE blots of variant response to temperature shift and CHX chase. SDS-PAGE blots showing distribution of variant band B and C glycoforms. HEK293 cells were transfected with pBI-CMV2 empty vector or vectors containing WT-CFTR or any of the 64 CFTR variants tested and incubated for 48 h at 37°C. The cells were subsequently incubated at 27°C for 24 h. After 24 h at 27°C, HEK293 cells transiently expressing CFTR were washed and incubated at 37°C in regular medium supplemented with 50 µM of cycloheximide (CHX) final concentration for 0, 10, 30, 60, 120, 240 min. Raw data used to generate the TrIdx at 37°C and 27°C and the stability of band C (StabC) at 37°C of each variant are shown. The two lanes per condition represent biological replicates.

Supplementary Figure 8. Comparison of TrIdx measured in FRT, HeLa [1-4] and HEK293 cells for each variant at 37°C. (**a**) Shown is the % of TrIdx (band C/total (B + C) ratio) in HEK293 (n=2 biological replicates), FRT and HeLa cells relative to WT for the 64 variants included in the study. (**b**) Comparison of TrIdx for each variant in FRT and HEK293 cells. (**c**) Comparison of TrIdx for each variant in HeLa and HEK293 cells. A comparison of the normalized TrIdx obtained from HEK293 cells to the TrIdx calculated from previously published data [1-4] from FRT and HeLa cells revealed PCCs of 0.94 and 0.92, respectively, suggesting that the TrIdx of CFTR variants does not differ significantly between highly divergent heterologous cell lines.

Supplementary Figure 9-14. FMP measurement of variants function at 37°C and 27°C. Representative FMP traces of the null (-/-), WT and the 64 variants at 37°C (orange) and 27°C (blue). HEK293 cells were transfected with pBI-CMV2 empty vector or vectors containing WT-CFTR or any of the 64 CFTR variants tested and incubated for 48 h at 37°C. The cells were

subsequently incubated at 27°C for 24 h. After 24 h at 27°C, HEK293 cells transiently expressing CFTR were washed twice in phosphate-buffered saline and incubated for 30 min with the blue membrane potential dye dissolved in a modified PBS at 37°C. The plates were read in a fluorescence plate reader at 37°C following a dye equilibration period of 30 min. CFTR was stimulated with an activation cocktail (10 μM forskolin and 50 μM genistein; final concentration) diluted in the same buffer. Fluorescence was monitored every 0.5 second for a total of 30 sec. CFTR-mediated depolarization of the membrane was monitored as an increase in fluorescence and hyperpolarization or repolarization as a decrease. Raw data used to generate the ClCon at 37°C and 27°C of each variant.

Supplementary Figure 15. Comparison of function in FRT[1-4] and HEK293 (HEK) cells for each variant at 37°C. (**a**) Shown is the % of the ClCon for each variant in HEK293 and FRT cells relative to WT. (**b**) Comparison of the ClCon for each variant observed in HEK293 and FRT cells. A comparison of the normalized ClCon between HEK293 cells and FRT cells[5] yielded a PCC score of 0.80, demonstrating that the basic functionality of human variants is conserved between highly divergent heterologous cell lines (**left panel**). To ensure that the good correlation between the normalized ClCon in HEK 293 and FRT cells is not entirely dependent on the variants which exhibit no function, we removed from the comparison all FRT variants previously published with less than 5% function (normalized to WT) leaving 27 variants (**right panel**). A comparison of these 27 variants yielded a PCC score of 0.68.

Supplementary Figure 16. Trafficking and functional response of a library of WT and 10 CFTR variants in HBE cells after temperature shift. (**a**) SDS-PAGE blots showing distribution

of variant band B and C glycoforms. -/- CFBE41o- -YFP cells were transduced with adenovirus carrying WT CFTR or P67L, G85E, E92K, S492F, F508Del, G551D, R560T, L1077P, M1101K and N1303K CFTR variants and incubated for 48 h at 37°C. The cells were subsequently incubated at 27°C or 37°C for 24 h. The two lanes per condition represent biological replicates. (**b**) Effect of temperature shift on CFTR variant activity. Quantification of YFP-quenching following incubation at 27oC or 37oC for 24 h of -/- CFBE41o- -YFP transduced cells with P67L, G85E, E92K, S492F, F508del, G551D, R560T, L1077P, M1101K and N1303K variants (n=2 biological replicates). Data are presented as a YFP fluorescence quenching relative to -/- CFBE410- -YFP cells transduced with WT CFTR incubated at 37oC for 24 h.

Supplementary Figure 17. VSP prediction validation by external datasets. (**a**) Table representing the 21 variants from two separate studies [6,7] used for validation and their measured and VSP predicted phenotype traits (i.e., TrIdx and ClCon). (**b, d**) Position of the 21 external variants on the ClCon and TrIdx landscapes, respectively. These validation variants have diverse relationships between TrIdx (band C/total (B + C) ratio) and ClCon. They include variants with a deficient TrIdx and no ClCon, as well as a wide range of TrIdx values associated with variable ClCon. The variants in the validation dataset are distributed across the entire CFTR sequence. (**c, e**) PCC-value, and the p-value (ANOVA test) with null hypothesis with the coefficient equal to zero between the measured and predicted ClCon and TrIdx, respectively.

Supplementary Figure 18. Correlation between ΔΔG° and CFTR trafficking and function. (**a**) ΔΔG° between WT-CFTR and each CFTR variant included in the study at 37°C. ΔΔG° is calculated using FoldX (n=5 *in silico* replicates) [8-10], the error bars represent the standard deviation

from 5 replicates. (**b**) Correlation between the FoldX calculated ΔΔG° and the *in viv*o measured CFTR TrIdx. (**c**) Correlation between the FoldX calculated ΔΔG° and the *in vivo* CFTR ClCon.

Supplementary Figure 19. Sequence alignment of CFTR from Homo sapiens, Bos taurus, Rattus norvegicus, Mus musculus and Xenopus laevis. The sequence fragments containing F508, DAD di-acidic ER export code and ICL4 are presented. Sequences were obtained from uniport (https://www.uniprot.org/). Sequence alignment was done by Clustal omega (https://www.ebi.ac.uk/Tools/msa/clustalo/). Figure was generated by ESPript 3.0 (https://espript.ibcp.fr/).

Supplementary Figure 20. Correlation between variant trafficking defect from the ER and stability at the cell surface. Representation of variant TrIdx (band C/total (B + C) ratio; x-axis) and stability at the cell surface (y-axis) of the 64 variants included in the study. A comparison of the normalized TrIdx obtained from HEK293 cells (**Fig. 2a**, upper panel) to the StabC (**Fig. 4a**), both calculated from **Supplementary Figure 1-7**, revealed a Pearson correlation coefficients (PCC) of 0.87, suggesting that the TrIdx from the ER and the stability at the cell surface of CFTR variants is highly correlated.

Supplementary Figure 21. Molecular variograms describing the behavior of CFTR variants at 37°C and 27°C. The spatial relationships of all possible variant pairwise combinations representing the spatial variance of ClCon and the distance value defined by VarSeqP and TrIdx (band C/total (B + C) ratio) are plotted (black dots for 37°C and red dots for 27°C). Statistical comparisons of the global spatial variation and global distance are shown as box and whisker plots at the right and top margins respectively (box = 25th and 75th with median line inside the box,

whisker length = outmost data point in the inner fence, square = mean, asterisks = min and max; p-value by Student's two tailed t-test).

Supplementary Figure 22. Optimization of variant expression and production. (**a**) Relative GFP fluorescence monitored after transfection of HEK293 cells with 0, 12, 25, 50 100 or 200 ng of pBI-CMV2-WT-CFTR or pBI-CMV2-F508del-CFTR after 24, 48 or 72 h (n=2 biological replicates). (**b** and **c,** upper panel). SDS-PAGE blots showing distribution of WT and F508del band B and C glycoforms as well as GFP. HEK293 cells were transfected with 0, 12, 25, 50, 100, 200 ng of pBI-CMV2 vectors containing WT-CFTR or F508del-CFTR and incubated for 48 h at 37°C. We observe a similar level of GFP-fluorescence at a given time-point when HEK293 cells were transfected with the same quantity of either pBI-CMV2-WT-CFTR or pBI-CMV2-F508Del-CFTR. In all cases, the level of GFP- fluorescence and the level of CFTR generated by either the pBI-CMV2-WT-CFTR or pBI-CMV2-F508del-CFTR using immunoblotting were directly comparable. These data validate the use of GFP fluorescence as a metric to ensure uniform transfection efficiencies across the range of variants tested.

Supplementary Figure 23-94. Uncropped and unedited blot/gel images related to this study.

Supplementary References

1 McCague, A. F. *et al.* Correlating Cystic Fibrosis Transmembrane Conductance Regulator Function with Clinical Features to Inform Precision Treatment of Cystic Fibrosis. *Am J Respir Crit Care Med* **199**, 1116-1126, doi:10.1164/rccm.201901-0145OC (2019).

2 Sharma, N. *et al.* Capitalizing on the heterogeneous effects of CFTR nonsense and frameshift variants to inform therapeutic strategy for cystic fibrosis. *PLoS Genet* **14**, e1007723, doi:10.1371/journal.pgen.1007723 (2018).

3 Sosnay, P. R. & Cutting, G. R. Interpretation of genetic variants. *Thorax* **69**, 295-297, doi:10.1136/thoraxjnl-2013-204903 (2014).

4 Sosnay, P. R. *et al.* Defining the disease liability of variants in the cystic fibrosis transmembrane conductance regulator gene. *Nat Genet* **45**, 1160-1167, doi:10.1038/ng.2745 (2013).

5 Sosnay, P. R. *et al.* Defining the disease liability of variants in the cystic fibrosis transmembrane conductance regulator gene. *Nature genetics* **45**, 1160 (2013).

6 Van Goor, F., Yu, H., Burton, B. & Hoffman, B. J. Effect of ivacaftor on CFTR forms with missense mutations associated with defects in protein processing or function. *Journal of Cystic Fibrosis* **13**, 29-36 (2014).
7 Yu, H. *et al.* Ivacaftor potentiation of multiple CFTR channels with gating mutations. *J Cyst Fibros* **11**, 237-245, doi:10.1016/j.jcf.2011.12.005 (2012).
8 Buss, O., Rudat, J. & Ochsenreither, K. FoldX as Protein Engineering Tool: Better Than Random Based Approaches? *Comput Struct Biotechnol J* **16**, 25-33, doi:10.1016/j.csbj.2018.01.002 (2018).
9 Buß, O., Rudat, J. & Ochsenreither, K. FoldX as protein engineering tool: better than random based approaches? *Computational and structural biotechnology journal* **16**, 25-33 (2018).
10 Delgado, J., Radusky, L. G., Cianferoni, D. & Serrano, L. FoldX 5.0: working with RNA, small molecules and a new graphical interface. *Bioinformatics* **35**, 4168-4169, doi:10.1093/bioinformatics/btz184 (2019).
