# Supplementary material for: Spatial covariance analysis reveals the residue-by-residue thermodynamic contribution of variation to the CFTR fold
Source: Commun Biol. 2022 Apr 13;5:356. doi: 10.1038/s42003-022-03302-2 (PMC9008016; doi:10.1038/s42003-022-03302-2)
Supplement: Supplementary file 3 — Description of Additional Supplementary Files [file 42003_2022_3302_MOESM3_ESM.pdf]

Description of Additional Supplementary Files

File name: Supplementary Data 1
Description: Variogram constructions and cross-validations.

File name: Supplementary Data 2
Description: Differential Energetics based Correction.

File name: Supplementary Data 3
Description: Raw Data Quantification.
